# ﻿Revisiting the genus *Tulipa* (Liliaceae) in Kazakhstan, the country with the richest tulip diversity worldwide

**DOI:** 10.3897/phytokeys.250.136736

**Published:** 2024-12-23

**Authors:** Serik A. Kubentayev, Shukherdorj Baasanmunkh, Daniyar T. Alibekov, Komiljon Sh. Tojibaev, Nudkhuu Nyamgerel, Anna A. Ivashchenko, Zagarjav Tsegmed, Vladimir G. Epiktetov, Gulnara T. Sitpayeva, Klara S. Izbastina, Zhansaya T. Idrissova, Saule K. Mukhtubayeva, Nurganym B. Abubakirova, Hee-Young Gil, Hyeok Jae Choi

**Affiliations:** 1 Astana Botanical Garden, Astana 010016, Kazakhstan Astana Botanical Garden Astana Kazakhstan; 2 Department of Biology and Chemistry, Changwon National University, Changwon 51140, Republic of Korea Changwon National University Changwon Republic of Korea; 3 Institute of Botany, Academy of Sciences of Uzbekistan, Tashkent 100125, Uzbekistan Institute of Botany, Academy of Sciences of Uzbekistan Tashkent Uzbekistan; 4 Institute of Zoology, Almaty 050000, Kazakhstan Institute of Zoology Almaty Kazakhstan; 5 Institute of Botany and Phytointroduction, Almaty 050000, Kazakhstan Institute of Botany and Phytointroduction Almaty Kazakhstan; 6 Department of Forest Biodiversity and Herbarium, Korea National Arboretum, Pocheon, Republic of Korea Korea National Arboretum Pocheon Republic of Korea

**Keywords:** Conservation, endemism, species pattern, threatened species, *Tulipa*

## Abstract

The genus *Tulipa* L., belonging to the Liliaceae family, has significant economic, horticultural, and ecological importance and is culturally revered in various regions worldwide. The total number of *Tulipa* species, including 90–120 taxa, varies based on different sources. Globally, Kazakhstan has one of the highest diversities of *Tulipa* species, most of which are threatened. In this study, we update and revise the *Tulipa* species in Kazakhstan based on field observations and an extensive herbarium specimens’ survey. A total of 41 taxa were identified, including 13 species endemic to Kazakhstan, among which seven species have been assessed as threatened globally. Furthermore, we gathered 1,942 occurrence records of 41 *Tulipa* taxa to analyze spatial arrangement of *Tulipa* species richness in Kazakhstan. Based on these results, we identified 22 grid cells with a high diversity of tulip species in southern Kazakhstan. We also present taxonomic key for all *Tulipa* species occurring in Kazakhstan, along with comments on their general distribution, habitat, phenology, distribution map, and conservation status. In addition, a color plate for each species is provided. Overall, our study provides valuable insights into the conservation status, distribution patterns, and biodiversity of *Tulipa* species in Kazakhstan, laying a foundation for targeted conservation efforts and further research in the region.

## ﻿Introduction

*Tulipa* L. species, belonging to the Liliaceae family, have significant economic, horticultural, ecological, and aesthetic importance, are culturally revered in various regions worldwide (Pavord 1999). However, the total number of *Tulipa* species varies among sources; for example, 104, and 95 taxa have been accepted in the World Checklist of Selected Plant Families (WCSP 2024) and Plants of the World Online (POWO 2024), respectively. Although Christenhusz et al. (2013) revised the *Tulipa* genus to include 76 accepted species, several new *Tulipa* species have since been discovered (de Groot and Zonneveld 2020; Rukšāns and Zubov 2022; de Groot and Zonneveld 2024).

The bulbs of *Tulipa* spp. are covered with a thin tunic that is glabrous or hairy. Typically, large flowers are trimerous, comprising two whorls of three brightly colored tepals, with the two whorls sometimes differing in color or having differently colored blotches at the base of each tepal (Hall 1940; Botschantzeva 1962). In addition, *Tulipa* species are identified based on various characteristics including capsule length, diameter, and shape; carpophore size, shape, and color; narrowed sterile upper portion of the capsule; and stigma shape, color and position (Botschantzeva 1962). More recently, Dekhkonov et al (2022) studied the morphological characteristics of 48 species from Central Asia, which are well supported at the section level.

Species of this genus occur naturally in temperate habitats, ranging from the Middle East, Central Asia, and North China to southern Europe and North Africa (Botschantzeva 1962; Zonneveld 2009; Christenhusz et al. 2013; Everett et al. 2013). *Tulipa* originated in Central Asia (Vvedensky and Kovalevskaya 1971); specifically, areas bounded by the Pamir-Alay and Tian Shan mountain ranges (Hoog 1973) are considered the primary centers of the genus diversity (Botschantzeva 1962; Dekhkonov et al. 2022). In particular, Kazakhstan has a high diversity of *Tulipa* species, with over 40 taxa (Ivashchenko and Belyalov 2019), followed by 34 taxa in Uzbekistan (Dekhkonov et al. 2021, 2022; Tojibaev et al. 2022) and 20 taxa in Turkey (Eker et al. 2014). Numerous researchers have studied the genus *Tulipa* based on conservation, prediction modeling, morphology, and molecular evidence. For example, Eker et al. (2014) revised the *Tulipa* species in Turkey based on many herbarium records and field observations. Similarly, Tojibaev et al. (2022) updated Uzbekistan *Tulipa* species using a taxonomic key and distribution. Meanwhile, Wilson et al. (2021) investigated the prediction modeling of several *Tulipa* species in Central Asia. Several studies have investigated the phylogenetic relationships among *Tulipa* species based on their plastomes and nuclear DNA sequences (Hajdari et al. 2021; Eker et al. 2024; Samartza et al. 2024; Sutula et al. 2024). In addition, complete plastome and comparative analyses have been conducted on several *Tulipa* species (Li et al. 2021; Yuan et al. 2022; Xing et al. 2023; Almerekova et al. 2024a, b; Tussipkan et al. 2024)

### ﻿A brief history of *Tulipa* in Kazakhstan

In the late 18^th^ century, P.S. Pallas surveyed Western Siberia and the adjacent territories and was the first to describe herbarium specimens of tulips from Kazakhstan (Ivashchenko and Belyalov 2019). A more detailed study of the diversity of *Tulipa* species in Kazakhstan began in the 19^th^ century by A. Lehman, I. Kirilov, I. Borshchow, L. Shrenk, and S. Karelin and E.A. von Regel, whose herbarium specimens have since been preserved, serving as invaluable materials for studying tulips. Accordingly, many plant species, including tulips, have been named after these scientists, including *Tulipaschrenkii* Regel, *T.borszczowii* Regel, and *T.lemmersii* Zonn., Peterse & J.de Groot (Ivashchenko and Belyalov 2019).

Specifically, in the second half of the 19^th^ century, E.L. Regel made an invaluable contribution studying Kazakhstani tulips by describing 17 tulip species (Ivashchenko and Belyalov 2019). One of the most remarkable species of tulips in terms of leaf shape, *Tuliparegelii* Krasn, is named in his honor. He distinguished the presence or absence of pubescence on the inner leaflets of the perianth and the base of the stamens as the main characteristics for species identification (Botschantzeva 1962). Aleksey Ivanovich Vvedensky also contributed greatly to the study of wild tulips in Central Asia, describing 17 new species of tulips in the 20^th^ century, of which six species were described in Kazakhstan. He provided the account of tulips in “Flora of USSR” (Vvedensky 1935), with changes in the tulip classification system including distinguishing two monotypic sections *Spiranthera* Vved. and *Lophophylon* Vved. He also processed the genus *Tulipa* in “Flora of Uzbekistan” (Vvedensky 1941), “Flora of Tajikistan” (Vvedensky 1963), and “Conspectus Florae Asiae Mediae” (Vvedensky and Kovalevskaya 1971).

Zinaida Petrovna Botschantzeva devoted her life to studying tulips in Central Asia, describing five new species and publishing a monograph, Tulips: morphology, cytology and biology” (Botschantzeva 1962). Her study provided a detailed description of 61 species of Central Asian wild tulips, including data on the geography, morphology, biology, and karyosystematics of tulips. Accordingly, *Tulipazenaidae* Vved. and *T.botschantzevae* S.N.Abramova & Zakal were named in honor of Z.P. Botschantzeva. Meanwhile, A.A. Ivashchenko has studied wild bulbous plants, including tulips, in Kazakhstan since 1963 (Ivashchenko 1987, 2005, 2007; Ivashchenko and Belyalov 2019). In addition to numerous publications on floristic studies in South Kazakhstan, she has published two monographs devoted to Tulips and other bulbous plants of Kazakhstan” (Ivashchenko 2005) and “Kazakhstan is the birthplace of Tulips” (Ivashchenko and Belyalov 2019). In Tulips and other bulbous plants of Kazakhstan” Ivashchenko described the cultivation, morphology and ecology peculiarities, geographical distribution, practical importance, and existing protection measures of 50 bulbous plants, including 34 species of tulips in Kazakhstan (Ivashchenko 2005). More recently, “Kazakhstan is the birthplace of Tulips” included 42 species of tulips, including colorful illustrations and maps of species distribution ranges in Kazakhstan (Ivashchenko and Belyalov 2019).

According to various sources, the number of tulips in Kazakhstan ranges from 32 to 42. For example, Polyakov (1958) revised the classification of wild tulips in “Flora of Kazakhstan” to include 32 species. Similarly, Abdulina (1999) updated the list of flora in Kazakhstan to include 34 tulip species. Later, Baitenov (2001), in treating the genus synopsis of the flora of Kazakhstan, reported 33 species of tulips. Recently, Ivashchenko and Belyalov (2019) updated and revised the tulips of Kazakhstan to include 42 species. Over the past 20 years, the new species of tulips have been described in Kazakhstan: *T.kolbintsevii* Zonn. (Zonneveld and de Groot 2012), *T.lemmersii* Zonn., Peterse & J.de Groot (Veldkamp and Zonneveld 2012), *T.ivasczenkoae* Epiktetov & Belyalov (Epiktetov and Belyalov 2013), *T.auliekolica* Perezhogin, *T.turgaica* Perezhogin (Perezhogin 2013), *T.annae* J.de Groot & Zonn, *T.dianaeverettiae* J.de Groot & Zonn. (de Groot and Zonneveld 2020), *T.salsola* Rukšāns & Zubov (Rukšāns and Zubov 2022), *T.jansii* J.J. de Groot & Zonn., and *T.kujukense* J. de Groot & Zonn. (de Groot and Zonneveld 2024). According to the latest checklist of endemic vascular plants, 13 species are endemic to Kazakhstan (Kubentayev et al. 2024). However, our understanding of overall distribution, species diversity, and conservation issues on Kazakhstan *Tulipa* is far from complete.

In the present study, we revisited the genus *Tulipa* in Kazakhstan based on field surveys (2020–2024) and extensive herbarium specimens. The primary aims of this study are to (i) provide an updated synopsis with the taxonomic keys and taxonomic notes on all species along with photographic documentation of wild plants, (ii) determine species diversity and distribution using all known occurrence records from various sources across Kazakhstan, and (iii) discuss future conservation actions for tulips.

## ﻿Materials and methods

### ﻿Plant distribution data

Species occurrences data were gathered from four main sources: (i) field survey observations between 2020 and 2024; (ii) literature (i.e. Ivashchenko and Belyalov 2019; de Groot and Zonneveld 2024) survey; (iii) herbarium collections from AA, ALTB, BRNU, СО, E, GB, KFTA, KG, KNU, KSPI, KUZ, L, LE, LECB, MHA, MW, NUR, O, TALL, TASH, TK, US (Thiers 2023) and (iv) the iNaturalist and Plantarium platforms. We collected herbarium specimens, which were deposited in the NUR, during the field surveys. Photographs of wild *Tulipa* species populations were captured by the authors during field surveys. Moreover, additional tulip photographs from the iNaturalist (https://www.inaturalist.org/) and Plantarium (https://www.plantarium.ru/) platforms were used.

A total of 2,496 occurrence records were gathered, including 821 herbarium specimens and 1,675 observations from iNaturalist (accessed on 01 Apr 2024). The coordinates and misidentification of each observation were critically examined on iNaturalist. After removing duplicates and doubtful records, 1,942 occurrence records were retained for species pattern analysis.

**Figure 1.**
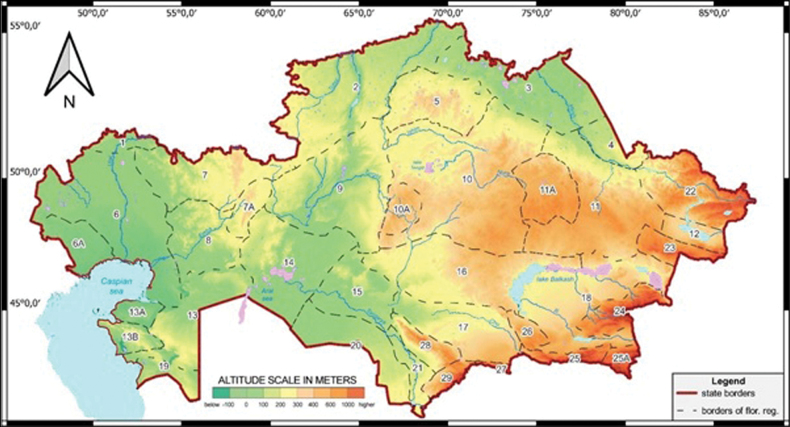
Map of the floristic division of Kazakhstan based on Pavlov (1956): 1 – Syrt, 2 – Tobol-Ishim, 3 – Irtysh, 4 – Semipalatinsk pine forest, 5 – Kokchetav, 6 – Caspian region, 6A – Bukeev, 7 – Aktobe, 7A – Mugojary, 8 – Emba, 9 – Turgay, 10 – Western Upland, 10A – Ulutau, 11 – Eastern Upland, 11A – Karkaraly, 12 – Zaysan, 13 – Northern Ustyrt, 13a – Buzachi, 13B – Mangyshlak, 14 – Aral region, 15 – Kyzylorda, 16 – Betpak -Dala, 17 – Moiynkum, 18 – Balkhash-Alakol, 19 – Southern Ustyrt, 20 – Kyzylkum, 21 – Turkestan, 22 – Altai, 23 – Tarbagatai, 24 – Dzungarian Alatau, 25 – Trans-Ili Kungey Alatau, 25A – Ketmen -Terskey Alatau, 26 – Chu-Ili Range, 27 – Kyrgyz Alatau, 28 – Karatau, 29 – Western Tian Shan.

For the characterization of each species distribution, the floristic division of Kazakhstan (Fig. 1) was divided into 29 regions and 7 sub-regions, based on Pavlov (1956).

### ﻿Species richness and conservations

We created a grid net for Kazakhstan with a spatial resolution of 0.5° × 0.5° grid size (equivalent to approximately 50 × 50 km^2^) using the FishNet tool in ArcGIS 10.3 (Esri 2012). The country was divided into 1294 grid cells. Additionally, three diversity measures were estimated using Biodiverse v.4.1 software: species richness (SR), weighted endemism (WE), and corrected weighted endemism (CWE), (Laffan et al. 2010). The WE was estimated by considering the presence or absence of a species within a cell, whereas the CWE was determined by calculating the proportion of endemic species within a cell relative to the total endemic SR of the cell (Laffan and Crisp 2003).

The conservation status of each species followed the Red Book of Kazakhstan (Baitulin 2014), which assumes three categories of rarity, i.e., I – very rare and critically endangered species, II – very rare species, and III – a rare species with a shrinking range. In addition, the global conservation status of the species, if assessed, was defined using the IUCN (2024) criteria.

### ﻿Phylogenetic analysis

To explore the evolutionary relationship of *Tulipa* spp. in Kazakhstan, internal transcribed spacer (ITS) sequences for 30 *Tulipa* species, collected only from Kazakhstan and belonging to three subgenera, were downloaded from the National Center for Biotechnology Information (NCBI) GenBank (Suppl. material 1: table S1). *Amanaedulis* (Miq.) Honda and *Erythroniumsibiricum* (Fisch. & C.A.Mey.) Krylov were selected as the outgroup based on previous studies. Briefly, the sequences were aligned using Clustal Omega (Sievers et al. 2020) as implemented in a Geneious Prime 2024.0.5 (http://www.geneious.com). The phylogenetic analyses were conducted using the maximum likelihood (ML) method in RAxML v.8.2.11 (Stamatakis 2006) with the best-scoring ML tree algorithm and 1000 bootstrap replicates. The reconstructed trees were visualized using Figtree v.1.4.2 (Rambaut 2012).

## ﻿Results and discussion

### ﻿Diversity of *Tulipa* in Kazakhstan

In this study, 41 *Tulipa* taxa, including 1 hybrid, Tulipa×tschimganica Botschantz., were identified (Suppl. material 1: table S2) in Kazakhstan, currently the highest number of taxa reported for a particular country in terms of the number of species and endemics. According to our previous study (Kubentayev et al. 2024), a total of 13 species— i.e., *T.alberti*, *T.auliekolica*, *T.annae*, *T.brachystemon*, *T.berkariensis*, *T.dianaeverettiae*, *T.ivasczenkoae*, *T.kolbintsevii*, *T.lemmersii*, *T.orthopoda*, *T.regelii*, *T.salsola*, and *T.turgaica* — were identified as being endemic to the country. However, two previously described endemic tulips from the present study, i.e., *T.annae* and *T.berkariensis* (Kubentayev et al. 2024) were excluded from the current study’s list of endemic tulips in Kazakhstan as *T.annae* also occurs in China (de Groot and Zonneveld 2020) and *T.berkariensis* is a synonym of *T.kaufmanniana* (Christenhusz et al. 2013; Everett et al. 2013; Sennikov and Tojibaev 2021). Instead, two newly described species endemic to Kazakhstan, *T.jansii* and *T.kujukense* (de Groot and Zonneveld 2024), were included on the list. Finally, a point distribution map was generated based on the herbarium (black dot) and observation records (red triangles) across the country for each species. A distribution map of Tulipa×tschimganica was created based on the *T.kaufmanniana* record, a parent species.

### ﻿*Tulipa* collection efforts in Kazakhstan

**Figure 2.**
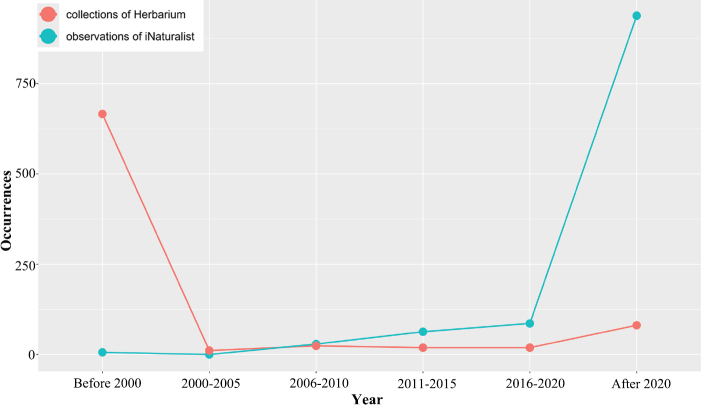
Number of herbarium specimen collection and observations on iNaturalist of *Tulipa* species in Kazakhstan.

The number of occurrences and collection years between the herbarium specimens and the observations were compared (Fig. 2). Most herbarium collections were found to be conducted before 2000. In contrast, the number of *Tulipa* observations on the iNaturalist platform has dramatically increased, owing to the efforts of numerous citizen scientists. Specifically, over 1,600 occurrences of 29 *Tulipa* species have been recorded by iNaturalist. Meanwhile, herbarium collections of *Tulipa* species in Kazakhstan have declined over the past 20 years, primarily due to many *Tulipa* species being threatened. In contrast, observations via photographic evidence have recently increased, which is beneficial for protecting threatened species and monitoring the current distribution of *Tulipa*.

### ﻿Phylogenetic relationship of *Tulipa* spp. in Kazakhstan

**Figure 3.**
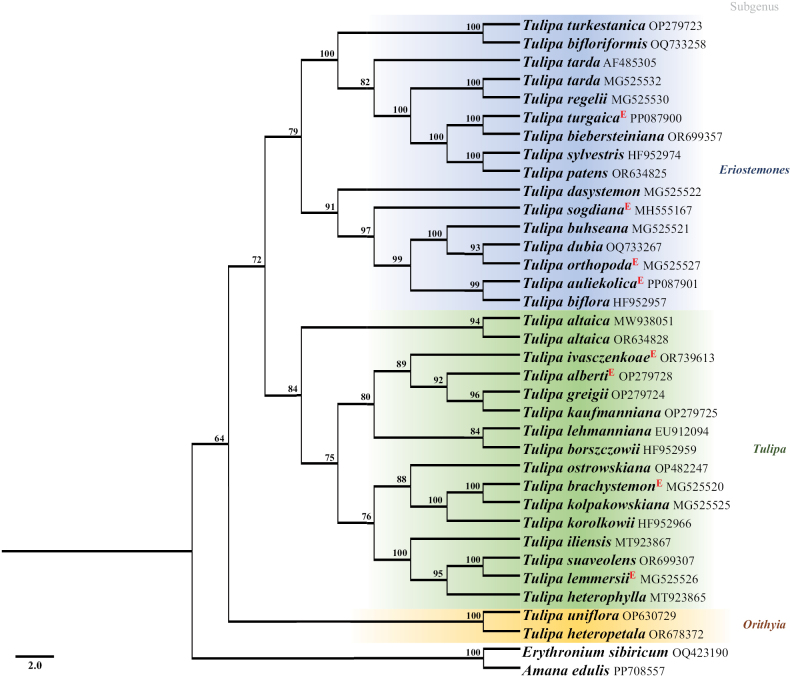
Phylogenetic tree of the *Tulipa* species based on the ITS sequence using the maximum parsimony method. Each subgenus is indicated as follows: *Orithyia* in yellow, *Eriostemones* in blue, and *Tulipa* in green. The endemic *Tulipa* species are indicated by E in red color.

A phylogenetic tree was generated for 43 accessions of 31 *Tulipa* species, including 7 endemic species (Fig. 3). The phylogenetic analysis of *Tulipa* was based on the nuclear ITS region with a length of 475 bp, of which 85 bp were parsimony-informative. *Tulipa* species from Kazakhstan formed a monophyletic phylogenetic tree with strong bootstrap values (Fig. 3). In general, *Tulipa* species were clustered into two main clades. The first included species of the subgenus Orithyia (Fig. 3), while the second clade was divided into two groups represented by species of the subgenera *Eriostemones* and *Tulipa* (Fig. 3), similar to the results of previous studies (Christenhusz et al. 2013; Hajdari et al. 2021; Chernysheva et al. 2023; Eker et al. 2024). Finally, about 90% of total tulip species from Kazakhstan were successfully sequenced (nrITS) and available on NCBI (Suppl. material 1: table S1). In addition, several studies have been investigating the complete plastome analysis (Almerekova et al. 2024a, b) and the population genetic (Yermagambetova et al. 2024) of *Tulipa* species in the country.

### ﻿Species richness and conservation of *Tulipa* in Kazakhstan

**Figure 4.**
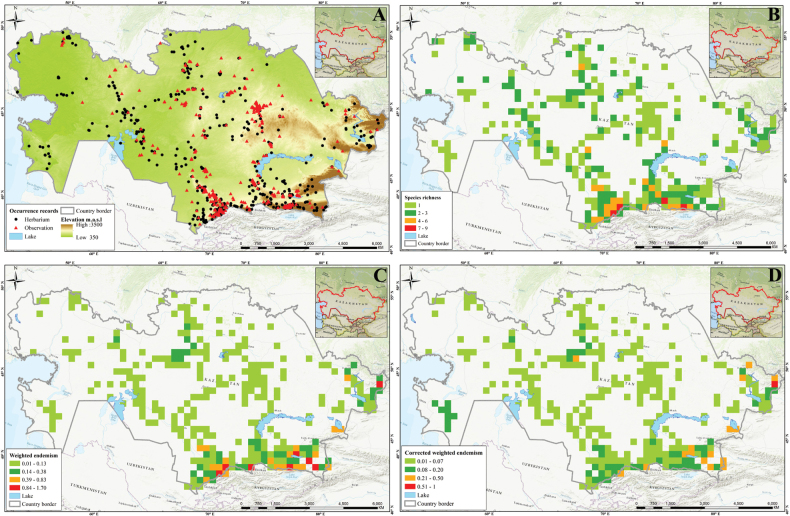
Species diversity of *Tulipa* in Kazakhstan **A** distribution map based on all records **B** species richness (SR) **C** weighted endemism (WE) **D** corrected weighted endemism (CWE).

Three different indices were analyzed (i.e., SR, WE, and CWE, for *Tulipa* species using 1,942 occurrences across the country (Fig. 4). The spatial distribution of records was assessed with most tulips collected in eastern Kazakhstan (Fig. 4A). In general, *Tulipa* species were unevenly distributed in the 295 grid cells across the country (Fig. 4B). For the SR, we identified 22 grids with high tulip SR, with 4–9 species concentrated in these grids, particularly in eastern Kazakhstan (Fig. 4B). The CWE ranged between 0.001 and 1, and the maximum values of 0.51–1 in a single grid cell were recorded for CWE. The WE ranged between 0.01 and 0.13, but a WE maximum value of 0.84–1.70 was recorded for the eight grid cells. In conclusion, based on the SR, WE, and CWE indices, most *Tulipa* species are distributed in the high mountains of southern and eastern Kazakhstan.

The distribution of *Tulipa* species in Kazakhstan was uneven, with most species recorded on the ridges of Western Tian Shan. The fewest species were recorded in the desert areas of southwestern Kazakhstan and the steppe regions of Northern and Central Kazakhstan. The highest concentrations of *Tulipa* species were documented in three floristic regions: Western Tian Shan (12 species), Trans-Ili Kungey Alatau (10 species) and Dzungarian Alatau (8 species). Additionally, 5–7 species were observed in 11 other floristic regions, namely Karatau (7 species), Moiynkum (7 species), Turgay (7 species), Altai (6 species), Betpak-Dala (6 species), Chu-Ili Range (6 species), Kyrgyz Alatau (6 species), Turkestan (6 species), Western Upland (6 species), Aral region (5 species), and Balkhash-Alakol (5 species); 2–4 species were identified in 15 floristic regions: Aktobe (4 species), Eastern Upland (4 species), Ketmen-Terskey Alatau (4 species), Mugojary (4 species), Tobol-Ishim (4 species), Zaysan (4 species), Bukeev (3 species), Caspian region (3 species), Kyzylorda (3 species), Syrt (3 species), Tarbagatai (3 species), Ulutau (3 species), Emba (2 species), Karkaraly (2 species), and Northern Ustyrt (2 species); and 1 species each was identified in Irtysh, Kokchetav, Kyzylkum, Mangyshlak, Semipalatinsk Pine Forest, and Southern Ustyrt. Meanwhile, no *Tulipa* species were detected in Buzachi (Suppl. material 1: table S2).

Generally, the distribution of tulip species was consistent with the findings of Asatulloev et al. (2023), who mapped and analyzed the distribution patterns in ecoregions and phytogeographic regions in Central Asia. The results also aligned with the studies of Ivashchenko and Belyalov (Ivashchenko 2005; Ivashchenko and Belyalov 2019), who distinguished two mountainous centers of species diversity in Kazakhstan (Tian Shan Zhetusy and Altai-Tarbagatai) and one plain center of tulip diversity, covering vast areas of steppes and deserts.

However, a new location for *T.altaica* was found in the current study in the Western Upland and Betpak-Dala in Kazakhstan, significantly extending the species’ general distribution range. Additionally, new localities of *T.auliekolica* were established in Northern Kazakhstan. Previously, this species was only reported in two localities, including the classical locality (15 km from the Karamendy). Moreover, *T.annae* was listed for the first time in the Dzungarian Alatau (Taskora Gorge), based on photographic observations by Kolbintsev (2016).

Of all 41 species of tulips in Kazakhstan, 18 species are listed in the Red Data Book of Kazakhstan (Baitulin 2014), including: Category I – two species (*T.biflora* and *T.lehmanniana*); Category II – eight species (*T.alberti*, *T.borszczowii*, *T.brachystemon*, *T.heteropetala*, *T.korolkowii*, *T.regelii*, *T.tarda*, and *T.zenaidae*); Category III – eight species (*T.greigii*, *T.kaufmanniana*, *T.kolpakowskiana*, *T.ostrowskiana*, *T.patens*, *T.suaveolens*, and *T.uniflora*) (Suppl. material 1: table S2). This categorization does not correspond to the current state of tulip rarity in Kazakhstan, requiring radical revision. Hence, a regional reassessment of all tulips according to the IUCN assessment criteria is required for the next edition of the Red Data Book of Kazakhstan. Additionally, according to long-term observations, *T.biflora*, *T.patens* and *T.biebersteiniana* should be excluded from the Red Data Book of Kazakhstan; they do not require protection. Meanwhile, the following five species should be included in the next edition of the Red Data Book of Kazakhstan: *T.dubia*, *T.iliensis*, *T.ivasczenkoae*, *T.lemmersii*, and *T.orthopoda.* Additionally, the regional conservation status for the potential protection of nine tulip species in Kazakhstan requires reassessment: *T.annae*, *T.jansii*, *T.kujukense*, *T.kolbintsevii*, *T.salsola*, *T.turgaica*, *T.auliekolica*, T.×tschimganica, and *T.dianaeverettiae*.

Based on the IUCN (2024) criteria, 27 tulips species from Kazakhstan were assessed at the global level. This analysis identified seven threatened species, comprising two critically endangered (CR) species (*T.dianaeverettiae* and *T.ivasczenkoae*), two endangered (EN) species (*T.regelii* and *T.kolbintsevii*), and three vulnerable (VU) species (*T.lemmersii*, *T.orthopoda* and *T.zenaidae*). The remaining 20 species were assessed as near threatened, comprising 10 near threatened (NT) species (*T.alberti*, *T.borszczowii*, *T.dubia*, *T.iliensis*, *T.kaufmanniana*, *T.kolpakowskiana*, *T.korolkowii*, *T.lehmanniana*, *T.ostrowskiana* and *T.uniflora*) and 10 least concern (LC) species (*T.altaica*, *T.bifloriformis*, *T.brachystemon*, *T.dasystemon*, *T.greigii*, *T.heteropetala*, *T.heterophylla*, *T.tarda*, *T.tetraphylla*, and *T.turkestanica*).

### ﻿Taxonomic key of *Tulipa* in Kazakhstan

#### ﻿Identification key for the sections of *Tulipa* in Kazakhstan

**Table:** 

1	Filaments glabrous, flowers generally bowl-shaped with a rounded base and predominantly with red or yellow color, if white ovary as long as stamens, with a very long style	**2**
–	Filaments with hairs at the base and/or soft hairs along their length; flowers funnel-shaped, with a slight constriction just above the base, and predominantly white or whitish-yellow color	**7**
2	Bulb tunics typically papery and glabrous inside; leaves 2; ovary as long as stamens, with a very long style	**sect. Orithyia**
–	Bulb tunics typically of varying consistency, from papery to coriaceous and covered with varying degrees of hair on the inside; leaves usually 2 to numerous; ovary slightly shorter than stamens, with sessile style	**3**
3	Anthers opening gradually and slowly (during 2–3 days) from tip to base and becoming contorted	**sect. Spiranthera**
–	Anthers opening rapidly, not becoming contorted	**4**
4	Leaves narrow, glaucous; bulb tunic extended, sometimes up to soil level	**sect. Kolpakowskianae**
–	Leaves often broad; bulb tunic not extended up to soil level	**5**
5	Leaves with anthocyan markings	**sect. Vinistriatae**
–	Leaves without anthocyan markings	**6**
6	Bulb tunics scales leathery, long hairy inside, more densely hairy basally and apically; stem pubescent; leaves erect-ascending, the lower leaf base is as high above the ground, glossy, pubescent adaxially, whitish-reddish margined, keeled; tepals red with yellow 3-dentate spot at base	**sect. Lanatae**
–	Bulb tunics weakly coriaceous, blackish-brown, more or less hairy all over; stem glabrous to more or less pubescent; leaves more or less spreading, scattered, glaucous, rather crisp, glabrous or pubescent; tepals polychrome in color, usually red, yellow or white, basal blotch black or yellow or absent	**sect. Tulipa**
7	Bulb tunics covered inside with some short, sometimes adpressed hairs on the top, middle part glabrous or ± glabrous; anthers oblong and without cusp	**sect. Sylvestres**
–	Bulb tunics covered inside with woolly, sometimes densely adpressed hairs on the top and base anthers terminating in a short cusp	**sect. Biflores**

#### ﻿Subgenus Tulipa

##### ﻿ Sect. Tulipa

Taxon classificationPlantaeLilialesLiliaceae

L.

794952BD-B8D4-53D9-B91A-BF4DDEED032F

Tulipa suaveolens Roth in Ann. Bot. (Usteri) 10: 44 (1794).

###### Type.

—*Tulipagesneriana* L., Sp. Pl. 1: 306 (1753).

##### ﻿ Sect. Lanatae

Taxon classificationPlantaeLilialesLiliaceae

(Raamsd.) Zonn. in Pl. Syst. Evol. 298: 90 (2012).

E61E11E5-3A92-5F2C-9087-FBE91A4DC9D6

Tulipa ivasczenkoae Epiktetov & Belyalov in Turczaninowia 16: 5 (2013).

###### Basionym.

Tulipaser.Lanatae van Raamsd., van Raamsd. & T. de Vries, Pl. Syst. Evol. 195: 40 (1995).

###### Type.

—*Tulipalanata* Regel, Trudy Imp. S.-Peterburgsk. Bot. Sada 8: 647 (1884).

##### ﻿ Sect. Kolpakowskianae

Taxon classificationPlantaeLilialesLiliaceae

Raamsd. ex Zonn. & Veldkamp., Pl. Syst. Evol. 298: 90 (2012).

8C67A5B5-3E5F-5244-851C-9BE72DA15B7D

###### Type.

—*T.kolpakowskiana* Regel, Trudy Imp. S. Peterburgsk. Bot. Sada 5: 266 (1877).

#### ﻿Identification key for Tulipasect.Kolpakowskianae in Kazakhstan

**Table:** 

1	Bulb tunic fibrous, splitting, black to tawny, prolonged to soil surface, inside densely covered with curly woolly hairs; stamens one-third to two-fifths the length of perigone; filaments black to purple	**2**
–	Bulb tunic not fibrous and splitting, black to dark brown, not prolonged or slightly prolonged (if prolonged up to soil surface, then lined with woolly hairs at the top and glabrous below); stamens two or three times shorter than the perigone, filaments yellow	**3**
2	Upper leaves exceeding or at least reaching the flower; aboveground part one-half to two-thirds as long as the underground part; tepals with a dark violet blotch on both surfaces	***T.borszczowii***
–	Upper leaves do not exceed the flower; aboveground part as long as or slightly longer than the underground part; tepals usually with a dark basal blotch on the inner surface, rarely on both surfaces	***T.lehmanniana***
3	Stem in upper part and peduncle pubescent	**4**
–	Stem and peduncle glabrous	**5**
4	Stem up to 7.5 cm long, reddish green leaves usually lying on the soil surface ; tepals with short hairs on both sides near the base, tepals outside dull pinkish-red with yellow margins	***T.annae***
–	Stem up to 35 cm long, green; leaves usually scattered on the stem, never lying on the soil surface, glabrous; tepals glabrous, outer tepals outside yellowish-gray with a pinkish haze between the center and narrow yellow margins	***T.altaica***
5	Tepals usually red, sometimes yellow-red or yellow; filaments black in the lower part and purple in the upper part, rarely in yellow form entirely yellow	***T.korolkowii***
–	Tepals usually yellow or yellowish, sometimes red filaments commonly concolorous	**6**
6	Filaments gradually dilated at base, expanded in the middle part	**7**
–	Filaments with nearly parallel margins, abruptly narrowed at apex	**9**
7	Leaves (3–4) 5 up to 7, very close together, all sublorate, surpassing the flower; flowers 1 or 2, up to 4	***T.tetraphylla***
–	Leaves (3) 4, spreading, commonly not surpassing the flower; flower usually solitary	**8**
8	Bulb tunics coriaceous; lowest leaf sublorate to linear-lanceolate, 0.7–1.5 cm broad; tepals mostly acute or acuminate; anthers half as long as filaments	***T.iliensis***
–	Bulb tunics papery, sometimes subcoriaceous; lowest leaf linear-lanceolate, 2–3 cm broad; tepals mostly obtuse; anthers as long as filaments	***T.brachystemon***
9	Tepals yellow, not blotched; leaves commonly surpassing the flower	**10**
–	Tepals usually red with a black basal blotch, sometimes yellow-red or yellow; leaves commonly not surpassing the flower	**12**
10	Plants small, up to 7 cm tall; tepals usually incurved	***T.lemmersii***
–	Plants taller, 10–15 cm tall; tepals usually not incurved	**11**
11	Bulb ovoid or usually slightly prolonged with fibers above the bulb; outer tepals oblong to oblong-rhomboidal, inner tepals oblong-oblanceolate to oblong; filaments straight, orange-yellow	***T.kolpakowskiana***
–	Bulb pear-shaped, tunics not prolonged and without fibers; outer tepals lanceolate, the tip and nearby margins covered with short hairs, inner tepals obovate; filaments ovate to almost straight, yellow, mottled with grayish	***T.jansii***
12	Tepals usually yellow, lower-most leaf oblong-lanceolate to oblong, 3–6 cm broad	***T.zenaidae***
–	Tepals usually red, lower-most leaf linear-lanceolate to lanceolate, 1–4 cm broad	***T.ostrowskiana***

##### ﻿ Sect. Vinistriatae

Taxon classificationPlantaeLilialesLiliaceae

(Raamsd.) Zonn., Pl. Syst. Evol. 298: 91 (2012).

DFE3856D-1DAF-5557-82A7-116DB4EE2920

###### Type.

—*Tulipagreigii* Regel in Gartenflora 22: 290, t. 773. (1873).

#### ﻿Identification key for Tulipasect.Vinistriatae in Kazakhstan

**Table:** 

1	Inner tepals obovate and slightly longer than the outer; leaves profusely violet-speckled on upper side	***T.greigii***
–	Inner tepals triangular-obovate and shorter than the outer; leaves without violet-speckled on upper side	***T.alberti***

##### ﻿ Sect. Spiranthera

Taxon classificationPlantaeLilialesLiliaceae

Vved. ex Zonn. & Veldkamp, Pl. Syst. Evol. 298: 90 (2012).

7F32FA2E-0352-5BD8-9885-7D4517FFA72F

###### Type.

—*Tulipakaufmanniana* Regel in Gartenflora 26: 194 (1877).

#### ﻿Identification key for Tulipasect.Spiranthera in Kazakhstan

**Table:** 

1	Relatively small plants, sometimes with almost sessile flowers; leaves curled or undulate; anthers almost equal in length with filaments or up to 1.5 times longer, not becoming strongly incurved or twisted; filaments rather thick	***T.dubia***
–	Relatively tall plants; flowers usually not sessile; leaves not curled or slightly undulate; anthers 2–4 times longer than filaments, when ripe the tips are curved down and outwards; filaments narrowly triangular	**2**
2	Anthers becoming strongly incurved or twisted; filaments yellow, dilated at the base; widespread in various habitats on all ranges of Kazakhstan part of Western Tian-Shan	***T.kaufmanniana***
–	Anthers not becoming strongly incurved or twisted; filaments yellow with brown apex, narrow, slightly dilated at the base; grows on stony-gravelly slopes of Karzhantau Range	***T.tschimganica***

##### ﻿ Subgenus Orithyia

Taxon classificationPlantaeLilialesLiliaceae

(D.Don) Baker, J. Linn. Soc. Bot. 14: 277 (1874).

572BB2A3-7DED-5D69-993A-E06C91EA640C

###### Lectotype.

*Tulipauniflora* (L.) Besser ex Baker.

##### ﻿ Sect. Orithyia

Taxon classificationPlantaeLilialesLiliaceae

(D. Don) Vved., Brit. Fl. Gard. [Sweet] Ser. 2: 336 (1836).

C05B29D4-3FEE-5922-8333-5AD0123B1E95

###### Type.

—*Tulipauniflora* Besser ex Baker, J. Linn. Soc., Bot. 14: 295 (1874).

#### ﻿Identification key for Tulipasect.Orithyia in Kazakhstan

**Table:** 

1	Bulb elongate-ovoid, tunics naked, leaves opposite	***T.heterophylla***
–	Bulb ovoid, tunics with appressed hairs inside at apex; leaves alternate; anthers 3–6 mm long	**2**
2	Leaves narrowly linear-lanceolate, usually slightly surpassing the flower with brown margins; tepals obtuse or subobtuse; filaments gradually attenuate from base	***T.uniflora***
–	Leaves much scattered, linear, glabrous, not surpassing the flower with reddish margins; tepals very acute; filaments dilated below the middle; anthers up to 9 mm long	***T.heteropetala***

##### ﻿ Sect. Sylvestres

Taxon classificationPlantaeLilialesLiliaceae

in Gard. Chron. 20: 233 (1883).

F1FDAA31-1BC0-51D2-94E8-E2732716C78B

###### Type.

—*Tulipasylvestris* Pall., Sp. Pl.: 305 (1753).

#### ﻿Identification key for Tulipasect.Sylvestres in Kazakhstan

**Table:** 

1	Tepals yellow, the outer often violet tinged on the outside	**2**
–	Tepals white, yellow at base, the outer greenish-gray on the outside, becoming darker toward the base	***T.patens***
2	Bulb tunics ca. 2–2.5 cm; ovary slightly shorter than stamens; forest lawns and meadows in river valleys	***T.biebersteiniana***
–	Bulb tunics up to 4.5 cm; ovary equal to or longer than stamens; dry steppe or semi-desert plains	***T.turgaica***

##### ﻿ Sect. Biflores

Taxon classificationPlantaeLilialesLiliaceae

A.D.Hall ex Veldkamp & Zonn., Pl. Syst. Evol. 298: 89 (2012).

8B36DAA9-D2AC-5891-8C74-3506BBA47735

###### Type.

—*Tulipabiflora* Pall., Reise Russ. Reich. 3: 727 (1776).

#### ﻿Identification key for Tulipasect.Biflores in Kazakhstan

**Table:** 

1	Filaments glabrous	***T.sogdiana***
–	Filaments with a ring of hairs at the base or scattered hairs along their length	**2**
2	Leaf solitary, with raised undulating ridges along its length	***T.regelii***
–	Leaves 2 to several, never with undulating ridges along its length	**3**
3	Bulb tunics glabrous; leaves 3–7, very close together; flowers 1–8	***T.tarda***
–	Bulb tunics always hairy to varying degrees, sometimes with few appressed hairs at apex (*T.dasystemon*); leaves commonly 2 or sometimes 3, scattered, spreading	**4**
4	Bulb tunics papery or nearly so	**5**
–	Bulb tunics coriaceous or sub-coriaceous	**9**
5	Flowers commonly yellow; bulb tunics blackish-brown	***T.auliekolica***
–	Flowers commonly white, creamy white with a yellow (whitish yellow) blotch; bulb tunics brown or grayish-brown	**6**
6	Stem 30–50 mm long, covered with short hairs; bulb tunics reddish-brown with woolly hairs at the top; capsule with a small dome on the top; at an altitude of 1800 m	***T.dianaeverettiae***
–	Stem up to 20–25 cm long, glabrous; bulb tunics light to dull brown, inside glabrous or covered with woolly hairs more densely at the top; capsule without dome on the top; below an altitude of 1800 m	**7**
7	Stamens slightly longer than ovary; anthers 2–3 mm long	***T.biflora***
–	Stamens shorter or equal to ovary; anthers 5–7 mm long	**8**
8	Bulbs globose or pear-shaped, inside glabrous, at the neck with some hairs; flowers solitary; stamens longer than the ovary; low bushes in Taskora and adjacent Kolasu Valleys of Dzjungarian Ala-Tau	***T.kolbintsevii***
–	Bulbs ovoid, inside in upper third thinly covered with thin, more or less parallel hairs, becoming more densely hairy apically; flowers 2(3); stamens shorter or equal to ovary; stabilized fixed and hilly-ridged sands over brown and gray-brown soils, in depressions of takyrs and solonchaks with sparse semi-desert/desert vegetation in Zhetysu region, extreme southern part of Dzungarian Alatau	***T.salsola***
9	Leaves almost opposite, very close together; style very short or practically absent; flower buds and flowers upturned; endemic of Karatau Ridge	***T.orthopoda***
–	Leaves mostly distant or more or less approximate; style short or with a rather long style; buds and flowers are not inverted; plants are more widespread in the desert and mountainous parts of Kazakhstan	**10**
10	Upper part of stem and peduncle pubescent; flower whitish with yellow blotch; style short; plants of plains, foothills and mid-mountains, up to 2400–2500 m	**11**
–	Upper part of stem and peduncle glabrous; flower pale yellow or whitish; style long; plants of high mountains, above 2400–2500 m	**14**
11	Leaves very scattered, usually shorter than flower; sandy and clay soils in predominantly arid plains	***T.buhseana***
–	Leaves more or less distant, commonly longer than the flower; foothills and mid-mountains of West Tian Shan	**12**
12	Bulb tunic brown-gray or reddish-brown, covered with woolly hairs	**13**
–	Bulb tunic dark brown, at the upper part densely covered with more appressed long silky hairs	***T.turkestanica***
13	Bulb tunic reddish-brown or pink, densely covered with woolly hairs on the inside, most of which are at the top and base filaments narrow triangular with hairs above the hairy rim	***T.bifloriformis***
–	Bulb tunic brown-gray, covered with felt-like short woolly hairs concentrated at the top; leaves narrower; filaments narrow triangular, glabrous above the hairy rim	***T.kujukense***
14	Bulb tunic usually light-brown or brown, papery, glabrous or covered with few straight, adpressed, white hairs at tip; anthers oblong; ovary scarcely shorter than stamens	***T.dasystemon***
–	Bulb tunic black to tawny, thin coriaceous, densely lined with woolly hairs at tip; anthers linear-oblong; ovary about the length of stamens	***T.dasystemonoides***

### ﻿Taxonomic status of *Tulipa* in Kazakhstan

**Figure 5.**
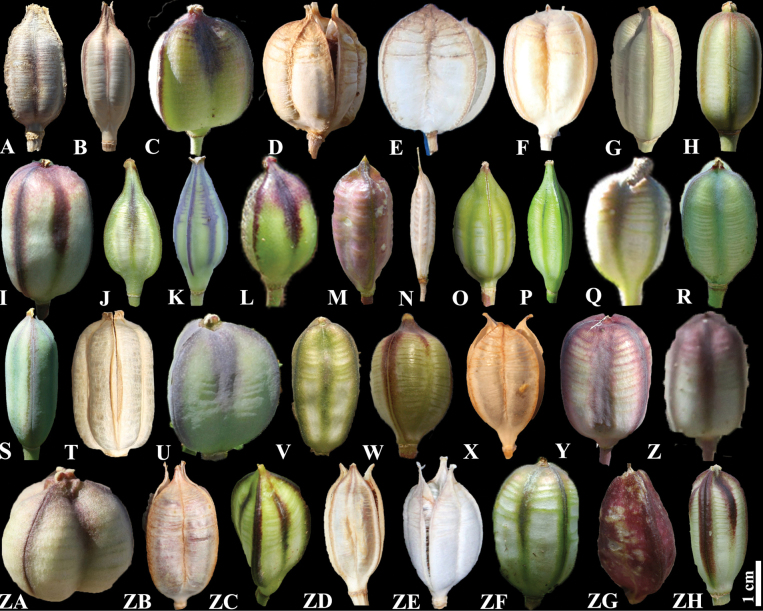
Capsules of *Tulipa* species in Kazakhstan **A***T.alberti***B***T.altaica***C***T.annae***D***T.auliekolica***E***T.biflora***F***T.bifloriformis***G***T.borszczowii***H***T.brachystemon***I***T.buhseana***J***T.dasystemon***K***T.dubia***L***T.greigii***M***T.heteropetala***N***T.heterophylla***O***T.ivasczenkoae***P***T.kaufmanniana***Q***T.kolbintsevii***R***T.korolkowii***S***T.kolpakowskiana***T***T.lehmanniana***U***T.lemmersii***V***T.orthopoda***W***T.ostrowskiana***X***T.patens***Y***T.regelii***Z***T.salsola***ZA***T.sogdiana* ZB *T.biebersteiniana***ZC***T.tarda***ZD***T.tetraphylla***ZE***T.turgaica***ZF***T.turkestanica***ZG***T.uniflora***ZH***T.zenaidae*.

Detailed taxonomic notes are provided for each species with phenology, conservation status, distribution, and type information. In addition, capsule characteristics are important for identifying *Tulipa* species, according to Botschantzeva (1962). Therefore, the capsules of 34 *Tulipa* species are illustrated based on our own and other sources (Fig. 5). According to Fig. 5, the capsule shape relatively differs among the studied species; however, further morphological studies on capsules of *Tulipa* species are needed.

#### ﻿ Tulipa alberti

Taxon classificationPlantaeLilialesLiliaceae

Regel, Gartenflora 26: 257, t 912 (1877).

38C896D1-2CA3-5419-9490-DCB8B7C2278D

Fig. 6

##### Type.

Kazakhstan • Illustration t. 912 in Gartenflora 26 (1877) [lectotype designated by Christenhusz et al. 2013: 303].

##### General distribution.

Endemic to Kazakhstan (Kubentayev et al. 2024).

##### Distribution in Kazakhstan and habitat.

Balkhash-Alakol, Betpak-Dala, Chu-Ili Range, Dzungarian Alatau, Eastern Upland, Karatau, Moinkym, Trans-Ili Kungey Alatau, and Western Upland. Grows on dry rubbly and stony slopes of low mountains.

##### Conservation status.

*Tulipaalberti* is assessed near threatened at the global level (IUCN 2024) and is included in the Red Book of Kazakhstan (Category II).

##### Phenology.

Flowering in April–May; fruiting in May–June.

##### Notes.

Eduard Regel described *T.alberti* in 1877, based on materials collected by his son Albert Regel, who worked as a doctor in Kulja, from the Karatau Mountains in 1876 (Ivashchenko and Belyalov 2019). The locus classicus of this species in the Chirchik River Valley was incorrectly stated when describing *T.alberti* (Vvedensky and Kovalevskaya 1971). More recently, Sutula et al. (2024) reported the hybridization of *T.alberti* and *T.patens* and the possible existence of a new undescribed species of hybridogenic origin. However, given that these species are morphologically different and belong to different subgenera, we believe that hybridization between these taxa is highly unlikely. Moreover, the distribution ranges of the species do not overlap, excluding the possibility of spontaneous hybridization in nature. Meanwhile, spontaneous hybrids of *T.alberti* and *T.greigii* exist in the wild (Ivashchenko and Belyalov 2019; Vvedensky and Kovalevskaya 1971). Recently, Yermagambetova et al. (2024) studied the genetic diversity and gene flow of *T.alberti* in Kazakhstan. In this work, the more isolated populations were determined to be genetically distinct with low genetic diversity (Yermagambetova et al. 2024).

**Figure 6.**
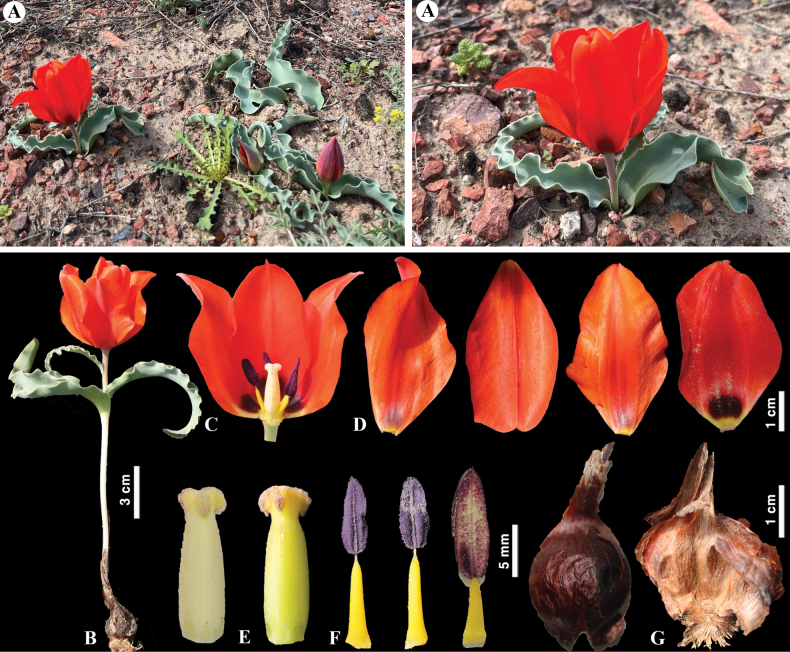
*Tulipaalberti* in Kazakhstan **A, B** general habits **C** flower **D** tepals **E** gynoecium **F** stamens **G** bulb sheath. (Photos: **A–G** by S. Kubentayev).

#### ﻿ Tulipa altaica

Taxon classificationPlantaeLilialesLiliaceae

Pall. ex Spreng., Syst. Veg., ed. 16 [Sprengel] 2: 63 (1825).

95611CBB-2323-5FEB-860E-6FD23255AC91

Fig. 7

##### Type.

• ‘Mons Imaus’ *Pallas* (not located). Neotype: Ledebour, Ic. Pl. Ross. 2: t. 134 (1830) [designated by Christenhusz et al. 2013: 304].

##### General distribution.

China (Xinjiang), Kazakhstan and Russia (Altai, west Siberia) (POWO 2024).

##### Distribution in Kazakhstan and habitat.

Altai, Betpak-Dala, Eastern Upland, Tarbagatai, Western Upland, and Zaysan. This species grows on stony slopes of low mountains and steppe areas at the foot of mountains, as well as among shrubs on rubbly slopes.

**Figure 7.**
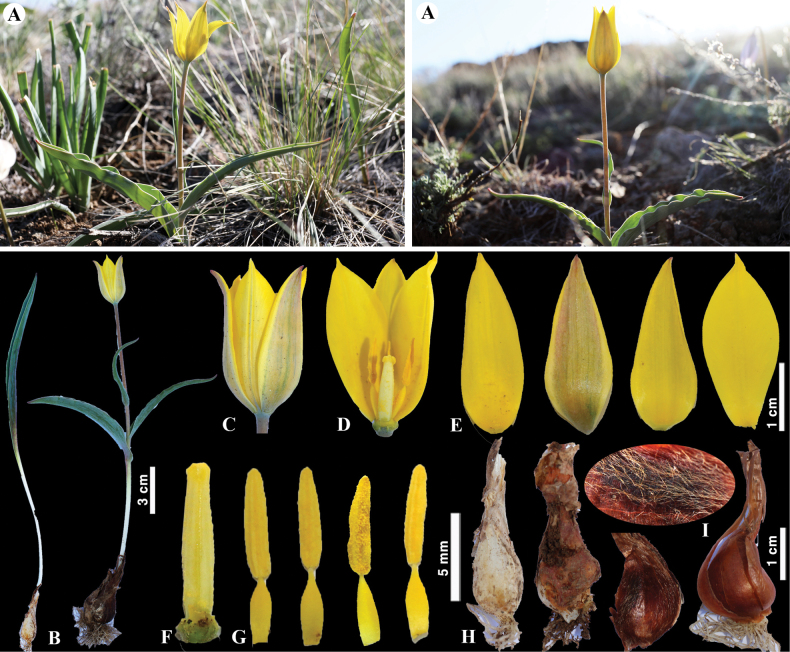
*Tulipaaltaica* in Kazakhstan **A** general habits **B** general appearance **C**, **D** flowers **E** tepals **F** gynoecium **G** stamens **H** bulb **I** bulb sheath. (Photos: **A–I** by S. Kubentayev).

##### Conservation status.

*Tulipaaltaica* is assessed as least concern at the global level (IUCN 2024).

##### Phenology.

Flowering in April–May; fruiting in May–June.

##### Notes.

This species was first collected by P.S. Pallas in Altai (Mount Imaus) in the late 18^th^ century. However, it was first described in 1825 by the German botanist Kurt Sprengel (Ivashchenko and Belyalov 2019). In the past, this species was recorded only in the Bektauata Mountain, Eastern Upland of Kazakhstan (Ivashchenko and Belyalov 2019; Kupriyanov 2020). In this study, however, a new location was found in the Kokshetau Mountains (Karamysheva *s.n.* LE), Western Upland of Kazakhstan, which significantly extended its distribution range.

#### ﻿ Tulipa annae

Taxon classificationPlantaeLilialesLiliaceae

J.de Groot & Zonn., Int. Rock Gard. 122: 10 (2020).

A7C2D22C-304E-5C12-BCBC-642FE8D501B6

Fig. 8

##### Type.

Kazakhstan • Marble Pass, Altai region, north-eastern Kazakhstan, 2008, *JJ de Groot* (holotype L 3986814).

##### General distribution.

China (Xinjiang) and Kazakhstan (de Groot and Zonneveld 2020).

##### Distribution in Kazakhstan and habitat.

Altai and Dzungarian Alatau. This species grows on cliffs, rocky and rubbly slopes, and mountainous plumes.

**Figure 8.**
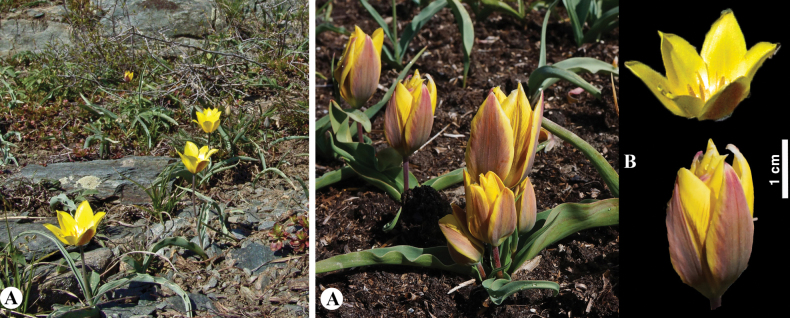
*Tulipaannae* in Kazakhstan **A** general habits **B** flowers. (Photos: **A, B** by J.J. de Groot).

##### Conservation status.

The IUCN conservation status of this species requires assessment.

##### Phenology.

Flowering in March–April; fruiting in May–June.

##### Notes.

*Tulipaannae* was described in 2020 from plants grown in a cultural collection in the Netherlands and bulbs collected at the Marble Pass in eastern Kazakhstan. This species is morphologically similar to *T.altaica* but differs by the presence of short hairs on the leaves, a smaller overall habit, the ovary shorter than the stamens and a tunic on the bulb elongated into a long spout. *Tulipaannae* is named after Anna Ivaschenko, a well-known botanist living and working in Almaty, Kazakhstan (de Groot and Zonneveld 2020). Based on the results of our research, this species was listed for the first time in the Dzungarian Alatau (Taskora Gorge) based on the photographic observations by Kolbintsev (2016). Due to the relative paucity of information on *T.annae* in the literature, further studies are needed regarding its distribution, abundance, and conservation status.

#### ﻿ Tulipa auliekolica

Taxon classificationPlantaeLilialesLiliaceae

Perezhogin, Novosti Sist. Vyssh. Rast. 45: 145 (2014).

F1CAC865-6FA1-5130-8529-D296F45278EE

Fig. 9

##### Type.

Kazakhstan • Prov. Kostanay, Auliekol distr., 25 April 2009, *Yu. Perezhogin s.n.* (LE).

##### General distribution.

Endemic to Kazakhstan (Kubentayev et al. 2024).

##### Distribution in Kazakhstan and habitat.

Tobol-Ishim and Turgay. This species grows in cereal steppes on plains.

**Figure 9.**
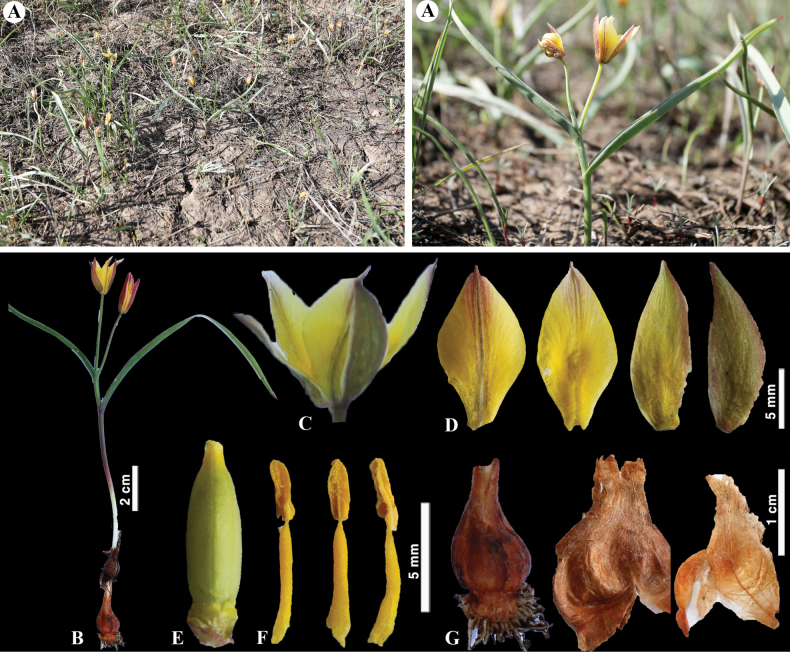
*Tulipaauliekolica* in Kazakhstan **A, B** general habits **C** flower **D** tepals **E** gynoecium **F** stamens **G** bulb and bulb sheath. (Photos: **A–G** by S. Kubentayev).

##### Conservation status.

The IUCN conservation status of this species requires assessment.

##### Phenology.

Flowering in April; fruiting expected from May to June.

##### Notes.

*Tulipaauliekolica* was first described in 2013 by Yu. V. Perezhogin from Kostanay region, Northern Kazakhstan. Morphologically, this species is similar to *T.biflora* but differs in its darker blackish-brown bulb tunics and yellow flower petals (Perezhogin 2013). The independence of this species raises doubts, as the known morphological characteristics are insufficient to assign the species rank. Additionally, *T.biflora* and *T.auliekolica* were placed in the same clade on the phylogenetic tree constructed using data from GenBank (Fig. 3). Accordingly, we consider it necessary to conduct phylogenetic and morphological studies to establish the taxonomic position of *T.auliekolica* in *Tulipa* genus. New localities of this species were established in Northern Kazakhstan during this study, according to herbarium collections of Yu. V. Perezhogin in the herbarium of the KSPI. Previously, the distribution of this species was only reported for two localities, including the type locality (15 km from the Karamendy turnoff) (Perezhogin 2013) and FR Turgay, north of the Akkuma sands (Perezhogin et al. 2015).

#### ﻿ Tulipa biebersteiniana

Taxon classificationPlantaeLilialesLiliaceae

Schult.f., Syst. Veg., ed. 15 bis [Roemer & Schultes] 7: 382 (1829).

99872511-62CD-5419-BE27-E24FF1393AEF

Fig. 10

##### Type.

Russia • ‘In hortis et vineis ad fluvium Terek inter Mosdok et Kisljar’, April, Bieberstein (not found).

##### General distribution.

Kazakhstan, Krym, North Caucasus, and Transcaucasus (Kutlunina et al. 2013).

##### Distribution in Kazakhstan and habitat.

Aktobe, Mugojary, Aral region, Western Upland, Ulytau, Syrt, Tobol-Ishim, Bukeev, Turgay, and Caspian region.

##### Conservation status.

The IUCN conservation status of this species requires assessment. It is included in the red book of Kazakhstan (CategoryIII).

##### Phenology.

Flowering in March–April; fruiting in May–June.

##### Notes.

*Tulipabiebersteiniana* was described in 1829 by J.H. Schultes from specimens from the North Caucasus (between Mozdok and Kizlyar). The species was named in honor of Russian botanist F. K. Biberstein-Marshall (Ivashchenko and Belyalov 2019). Some taxonomists classified *T.biebersteiniana* and *T.patens* as synonyms of the widespread European T.sylvestrissubsp.australis (Christenhusz et al. 2013; Everett et al. 2013). However, according to Zonneveld (2009), the difference in genome size between *T.biebersteiniana* (56.7 pg) and *T.sylvestris* (62.3 pg) may differentiate *T.biebersteiniana*. In 2013, Perezhogin (2013) described a new species, *T.turgaica* Perezhogin, similar to *T.biebersteiniana* and grows in arid conditions. It was suggested that *T.biebersteiniana*, should form its own subgenus based on hierarchical cluster analysis of genetic profiles of taxa conducted on Iranian tulips using AFLPs (Asgari et al. 2020). However, this hypothesis requires further investigation. Wilson (2023) included *T.biebersteiniana* on the list of tulips, which may require reinstatement if evidence confirms their uniqueness.

**Figure 10.**
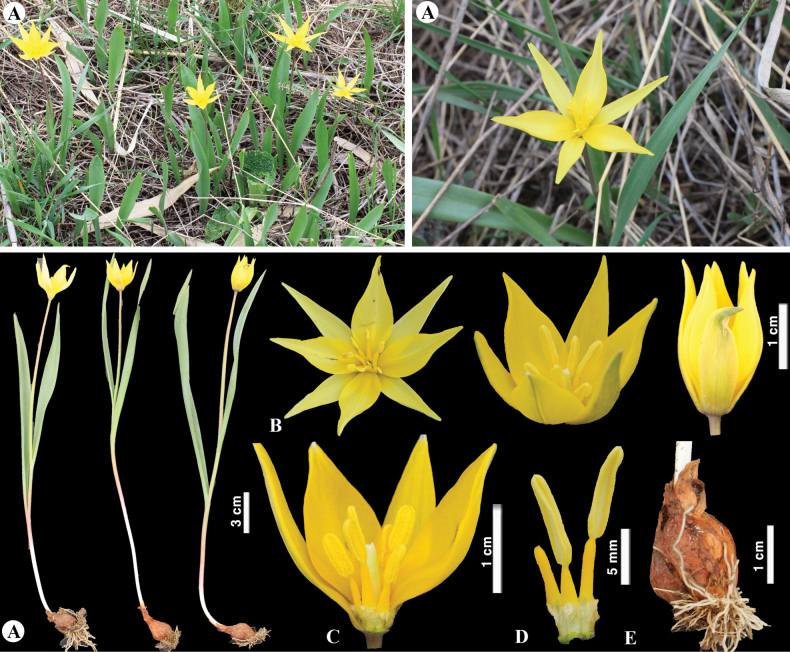
*Tulipabiebersteiniana* in Kazakhstan **A** general habits **B, C** flowers **D** ovary and stamen **E** bulb (Photos: **A–E** by S. Kubentayev).

#### ﻿ Tulipa biflora

Taxon classificationPlantaeLilialesLiliaceae

Pall., Reise Russ. Reich. 3: 727 (1776).

24AC80C5-FBD0-521D-8A99-74C66A876FB7

Fig. 11

##### Type.

Russia • Described from southern Russia, *Pallas* (lectotype BW000528948) [lectotype designated by Eker et al. 2014].

##### General distribution.

Kazakhstan, Uzbekistan (Northern Ustyurt), China (northern part), the south of Russia, Transcaucasia, and Crimea (Tojibaev et al. 2022).

##### Distribution in Kazakhstan and habitat.

Aktobe, Aral region, Betpak-Dala, Bukeev, Caspian region, Eastern Upland, Karkaraly, Mugojary, Northern Ustyrt, Turgay, Western Upland, and Zaysan. It grows on solonetz, clay steppe, and desert areas and less often on the stony and rubbly slopes of hills.

**Figure 11.**
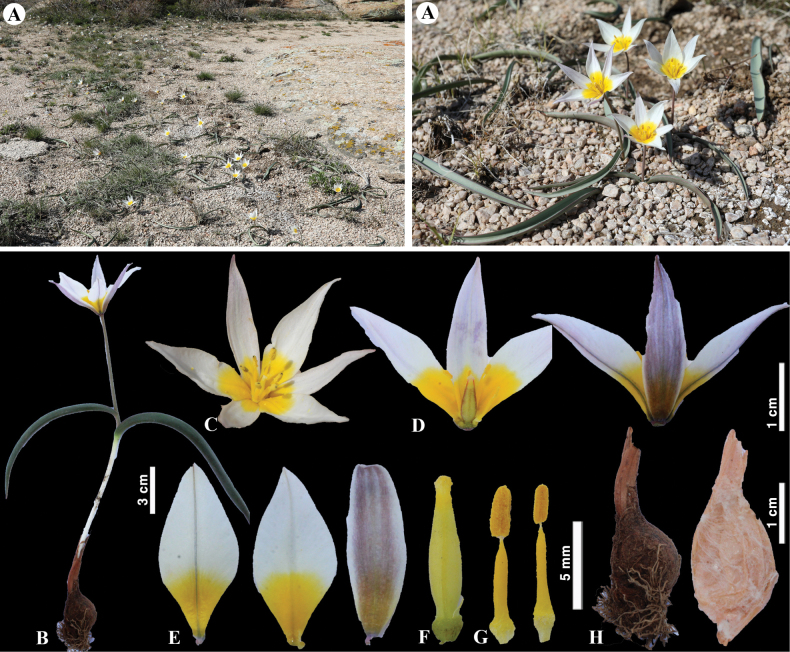
*Tulipabiflora* in Kazakhstan **A, B** general habits **C**, **D** flower **E** tepals **F** gynoecium **G** stamens **H** bulb and bulb sheath. (Photos: **A–H** by S. Kubentayev).

##### Conservation status.

The IUCN conservation status of this species requires assessment. It is included in the red book of Kazakhstan (Category I).

##### Phenology.

Flowering in April–May; fruiting in May–June.

##### Notes.

The species was described in 1776 by P. S. Pallas from specimens collected from the Caspian deserts. The location of the type specimens is unknown (Ivashchenko and Belyalov 2019). According to the latest system of *Tulipa* (Christenhusz et al. 2013), many species from the section Biflores A.D.Hall ex Veldkamp & Zonn., including *T.buhseana* and *T.sogdiana*, are considered synonyms of the *T.bifloras.l.* complex. However, we propose that *T.buhseana* and *T.sogdiana* should be considered independent taxa based on their complex morphological characteristics, ecology, and distribution range. In addition, these taxa were arranged in a separate clade from *T.biflora* in the phylogenetic tree (Fig. 3). The southern boundaries of the *T.biflora* range in Kazakhstan from west to east pass through the Northern Ustyurt, Aral region, Sarysu region sands, and the northern Balkhash region. Perezhogin (2013) considered the reports of *T.biflora* from Tobol-Ishim and Turgay erroneous. In his opinion, *T.biflora* was replaced with *T.auliekolica*. Given that it is impossible to distinguish *T.biflora* from *T.auliekolica* based on herbarium material, it is impossible to confirm or deny this statement.

#### ﻿ Tulipa bifloriformis

Taxon classificationPlantaeLilialesLiliaceae

Vved., Opred. Rast. Sred. Azii 2: 320 (1971).

8F312426-16C5-5945-AFD5-1703E7164CD1

Fig. 12

##### Type.

Uzbekistan • Tian-Schan occidentalis, in collibus argillosis circa urb. Taschkent, 19 March 1923, *M.G. Popov & A.I. Vvedensky* 1036 (holotype TASH000502!; isotypes BM, H1200916, K).

##### General distribution.

Kazakhstan, Kyrgyzstan, Tajikistan, and Uzbekistan (Tojibaev et al. 2022).

##### Distribution in Kazakhstan and habitat.

Karatau, Kyrgyz Alatau, Kyzylorda, Moiynkum, Turkestan, and Western Tian Shan. This species grows on the clay, stony, and rubbly slopes of hills, steppes and semi-desert foothill plains.

##### Conservation status.

*Tulipabifloriformis* is assessed as least concern at the global level (IUCN 2024).

##### Phenology.

Flowering in March–April; fruiting in May–June.

##### Notes.

*Tulipabifloriformis* was described by Vvedensky in 1935 from herbarium material collected near Tashkent in 1923. Later, a complete diagnosis of this species was published in ‘Conspectus Florae Asiae Mediae’ (Vvedensky and Kovalevskaya 1971). This species is morphologically similar to *T.biflora*, differing in its leathery bulb sheaths and drooping buds before flowering. Externally, *T.bifloriformis* is similar to *T.buhseana* but differs in its tomentose bulb sheaths on the inner side (Vvedensky and Kovalevskaya 1971). This species often inhabits open slopes after landslides and forms dense carpets that spread vegetatively (Tojibaev et al. 2022).

**Figure 12.**
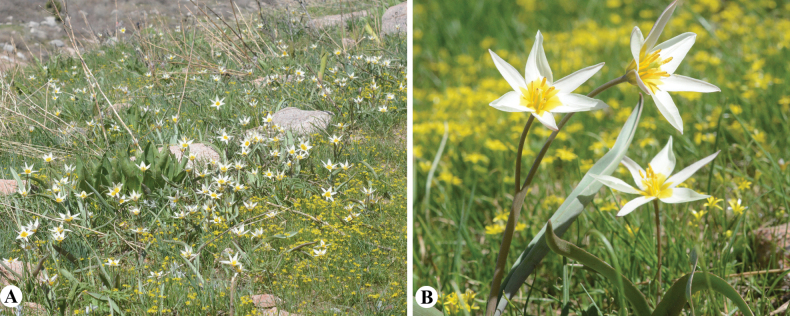
*Tulipabifloriformis* in Kazakhstan. **A** habitat **B** habit. (Photos: **A, B** by K.Tojibaev).

#### ﻿ Tulipa borszczowii

Taxon classificationPlantaeLilialesLiliaceae

Regel, Bull. Soc. Imp. Naturalistes Moscou 41: 438 (1868).

343F8AC4-CA1C-547C-80FF-C764BBE26F8D

Fig. 13

##### Type.

Kazakhstan • Steppe Kara-Kum am Aralsee, *Borszczow 677* (holotype LE!; photograph K).

##### General distribution.

Kazakhstan and Uzbekistan (POWO 2024).

##### Distribution in Kazakhstan and habitat.

Aral region, Betpak-Dala, Kyzylkum, Kyzylorda, Moiynkum, and Turkestan. This species grows in sandy and clay-sandy deserts.

##### Conservation status.

*Tulipaborszczowii* is a near threatened species at the global level (IUCN 2024). It is included in the red book of Kazakhstan (Category II).

##### Phenology.

Flowering in April–May; fruiting in May–June.

##### Notes.

*Tulipaborszczowii* was described by E.L. Regel in 1868 from the collections of Russian botanist I.G. Borshchow from Aral Karakum. The species is morphologically similar to *T.lehmanniana*, differing in its flower stalk that does not droop in buds, leaves exceeding or reaching the flower, and a shorter above-ground stem part that is 1.5–2 times shorter than the underground part (Ivashchenko and Belyalov 2019). This species was considered endemic to Kazakhstan until 2002, when it was found in Uzbekistan (from the western part of the Hungry Steppe) (TASH), and elsewhere in the Uzbek part of Kyzylkum (Abduraimov et al. 2020). The southernmost limit of its total range is in Uzbekistan, the northernmost limit is in the northern Aral region, and the range reaches the Transkaratau foothill plain in the east (Kyzylkol and Akkol lakes).

**Figure 13.**
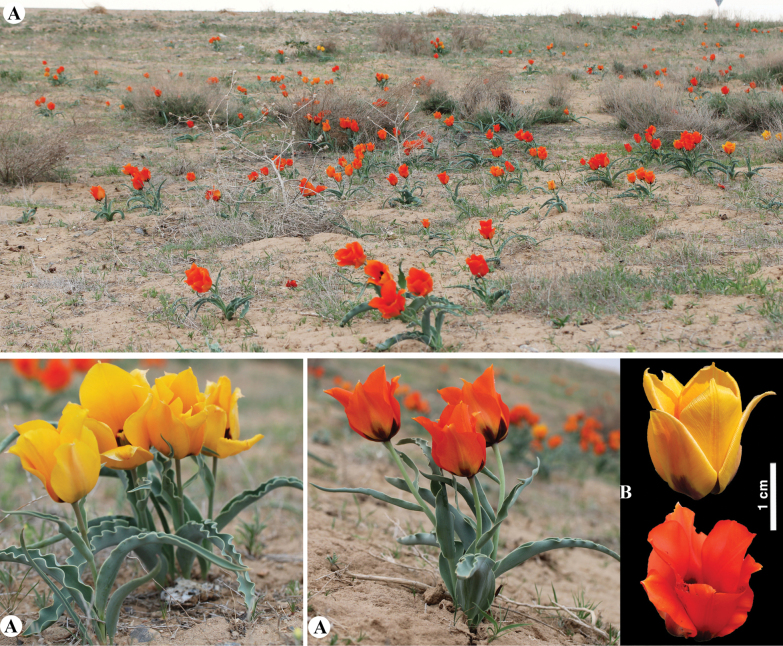
*Tulipaborszczowii* in Kazakhstan **A** general habits with different color forms **B** flowers. (Photos: **A, B** by S. Kubentayev).

#### ﻿ Tulipa brachystemon

Taxon classificationPlantaeLilialesLiliaceae

Regel, Gartenflora 323 (1882).

284ADE70-0702-5B45-9065-B3C7DFF2AF01

Fig. 14

##### Type.

• Illustration t. 1099, f. 2 in Gartenflora 31 (1882) [lectotype designated by Christenhusz et al. 2013: 325].

##### General distribution.

Endemic to Kazakhstan (Kubentayev et al. 2024).

##### Distribution in Kazakhstan and habitat.

Dzungarian Alatau. It grows on stony, rubbly slopes of the lower and middle belt of mountains in Dzungarian Alatau (up to 1700 m a.s.l.).

##### Conservation status.

*Tulipabrachystemon* is a least concern species at the global level (IUCN 2024). It is included in the red book of Kazakhstan (Category II).

##### Phenology.

Flowering in April–May; fruiting in May–June.

##### Notes.

*Tulipabrachystemon* was described by E.L. Regel in 1882, without the precise location of the type specimens. The LE herbarium contains collections of A. E. Regel from Schengeldy (Ivashchenko and Belyalov 2019). *Tulipabrachystemon* is listed as a synonym of *T.tetraphylla* in POWO (2024) and according to Christenhusz et al. (2013) and Everett et al. (2013). However, Zonneveld (2009) distinguished *T.brachystemon* as an independent taxon based on DNA barcoding. In addition, *T.brachystemon* and *T.tetraphylla* clustered in different clades based on our phylogenetic tree (Fig. 3). Thus, we consider it necessary to conduct further morphology and phylogeny studies to resolve the taxonomies of these two species.

**Figure 14.**
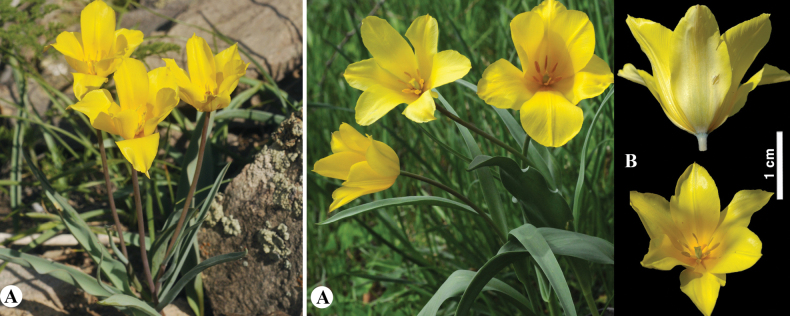
*Tulipabrachystemon* in Kazakhstan **A** general habits **B** flowers. (Photos: **A, B** by V. Epiktetov)

#### ﻿ Tulipa buhseana

Taxon classificationPlantaeLilialesLiliaceae

Boiss., Diagn. Pl. Orient. ser. 2, 4: 98 (1859).

54972286-5DC3-5E17-ACCE-690F4932E83D

Fig. 15

##### Type.

Iran • ‘Prope Yezd Persiae’, *Buhse* (holotype G).

##### General distribution.

Afghanistan, China, Kazakhstan, Iran, Turkmenistan, and Uzbekistan (Tojibaev et al. 2022).

##### Distribution in Kazakhstan and habitat.

Aral region, Balkhash-Alakol, Betpak-Dala, Chu-Ili range, Karatau, Kyzylorda, Moiynkum, Trans-Ili Kungey Alatau, Turgay, Turkestan, and Western Tian Shan. This species grows on sandy and clay deserts, and semi-deserts also occur on the rubbly slopes of lowlands.

##### Conservation status.

The IUCN conservation status of this species requires assessment.

##### Phenology.

Flowering in March–April; fruiting in May–June.

##### Notes.

*Tulipabuhseana* was described by P.E. Boissier in 1859 based on the collections of V.A. Buhse from Iran. Collections of *T.buhseana* from the northern Balkhash region had filaments with sparse pubescence, while the collections from the vicinity of the Shieli town, Kyzylorda region had glabrous filaments. According to Christenhusz et al. (2013), Zonneveld (2009), Everett et al. (2013) and POWO (2024), *T.buhseana* is considered a synonym of *T.biflora*. However, these species are morphologically distinguished by the lowering of bulb sheaths on the inner. Additionally, the bulb sheaths of *T.buhseana* are densely hairy on the inner side, whereas those of *T.biflora* are tomentose. In the phylogenetic tree, *T.buhseana* and *T.biflora* were placed in different clades (Fig. 3). Therefore, additional studies are required to determine the taxonomic position of *T.buhseana*.

**Figure 15.**
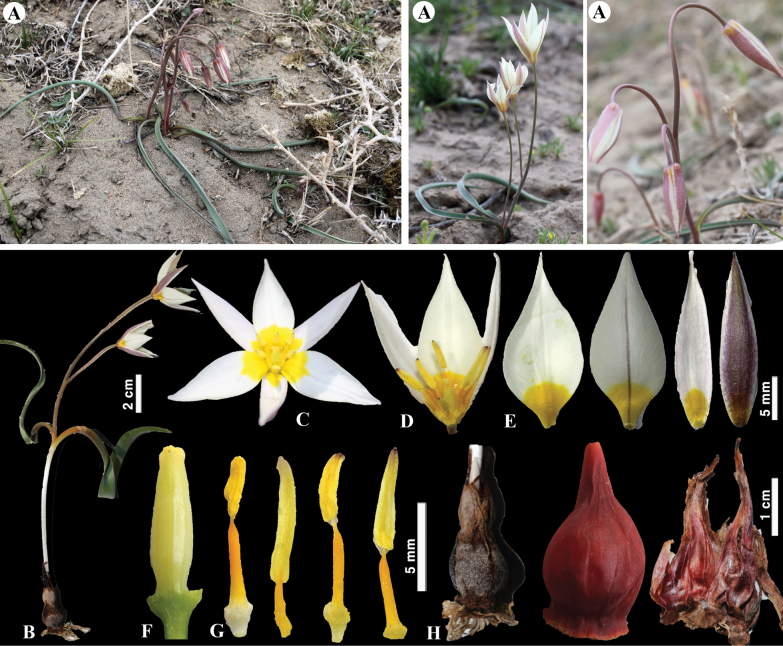
*Tulipabuhseana* in Kazakhstan **A, B** general habits **C, D** flower **E** tepals **F** gynoecium **G** stamens **H** bulb and bulb sheath. (Photos: **A–H** by S. Kubentayev).

#### ﻿ Tulipa dasystemon

Taxon classificationPlantaeLilialesLiliaceae

(Regel) Regel, Trudy Imp. S. Peterburgsk. Bot. Sada 6: 507 (1879).

B48374D1-318F-5FF1-A5E6-3472163D67B7

Fig. 16

##### Type.

Kazakhstan • ‘In montibus prope Wernoje ad fluvium Almatinka’, *A. Regel* (holotype LE; isotype PRC454341).

##### General distribution.

Kazakhstan, Kyrgyzstan, Tajikistan, Uzbekistan, and China (Xinjiang) (POWO 2024).

##### Distribution in Kazakhstan and habitat.

Ketmen Terskey Alatau, Kyrgyz Alatau, Trans-Ili Kungey Alatau. This species grows on steppe and meadow slopes, as well as on forest glades from the middle to the alpine belt (1900–3000 m a.s.l.).

##### Conservation status.

*Tulipadasystemon* is a least concern species at global level (IUCN 2024).

##### Phenology.

Flowering in April–June; fruiting in May–August.

##### Note.

*Tulipadasystemon* was described by E.L. Regel in 1879 from the Almaty vicinity (in the valley of the Almatinka River) (Regel 1879). Originally described as *Orithyiadasystemon* Regel, this species was later classified as *Tulipa* due to its long, narrow stems. In addition to the present *T.dasystemon*, A.I. Vvedensky (1935) wrote about a close species in Fergana Valley, which occurs at a lower rate and is characterized by strong and leathery bulb sheaths, potentially representing an undescribed species or demonstrating the ecological variability of *T.dasystemon*.

**Figure 16.**
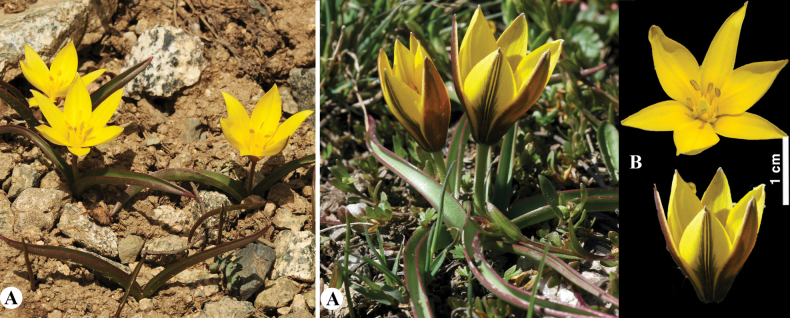
*Tulipadasystemon* in Kazakhstan **A** general habits **B** flowers. (Photos: **A, B** by V. Epiktetov).

#### ﻿ Tulipa dasystemonoides

Taxon classificationPlantaeLilialesLiliaceae

Vved., Byull. Sredne-Aziatsk. Gosud. Univ. 21: 147 (1935).

0D28DC00-15D3-5317-9EEF-8305937B023A

Fig. 17

##### Type.

Kazakhstan • ‘Altai Talac’, 11 June 1909, *Minkwitz 1365* (holotype LE!).

##### General distribution.

Kazakhstan, Kyrgyzstan, Tajikistan, and Uzbekistan (POWO 2024).

##### Distribution in Kazakhstan and habitat.

Kyrgyz Alatau, and Western Tian Shan. This species grows in meadows in the alpine belt of mountains and is sometimes found at the lower limit of the sub-alpine belt.

##### Conservation status.

The IUCN conservation status of this species requires assessment.

##### Phenology.

Flowering in May–June; fruiting in June–August.

##### Notes.

*Tulipadasystemonoides* was described by A.I. Vvedensky in 1935, based on herbarium material collected from the valley of the Maidantal River in Talas Alatau. Christenhusz et al. (2013) referred to *T.dasystemonoides* as being synonymous of *T.dasystemon*. However, *T dasystemonoides* is distinguished from *T.dasystemon* primarily by its dense woolly bulb sheaths and cream-colored flowers (Vvedensky 1935; Everett et al. 2013). In addition, the two species have different genome sizes (57.7 pg for *T.dasystemonoides* and 51.5 pg for *T.dasystemon*) (Zonneveld 2009). Therefore, we propose that *T.dasystemonoides* is an independent species.

**Figure 17.**
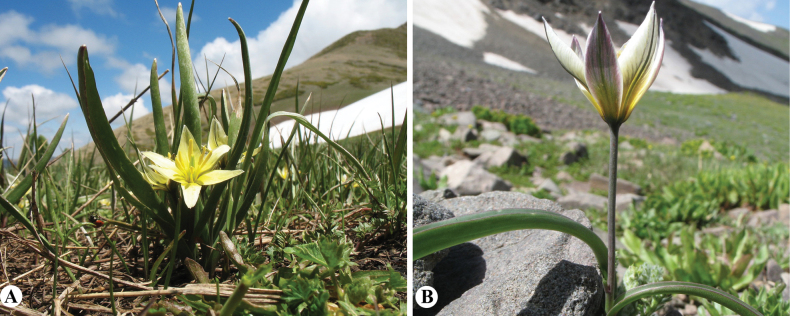
*Tulipadasystemonoides* in Kazakhstan **A** general habits. (Photos: **A, B** by V. Kolbintsev).

#### ﻿ Tulipa dianaeverettiae

Taxon classificationPlantaeLilialesLiliaceae

J.de Groot & Zonn., Int. Rock Gard. 122: 7 (2020).

88D8737A-3B6E-555A-8244-09D6B1D206B5

Fig. 18

##### Type.

Kazakhstan • Altai. Altai Pass, approximately 1800 m altitude, cult. *J.J. de Groot* (L 3986813).

##### General distribution.

Endemic to Kazakhstan (Kubentayev et al. 2024).

##### Distribution in Kazakhstan and habitat.

This species grows in open sunny places in dry sandy soil mixed with stones in the Altai.

##### Conservation status.

*Tulipadianaeverettiae* is a critically endangered species globally (IUCN 2024).

##### Phenology.

Flowering in April– May; fruiting in May–June.

##### Notes.

*Tulipadianaeverettiae* was described in 2020 by de Groot and Zonneveld (2020) from cultivated plants grown in the Netherlands from seeds collected by W. Lemmers in Kazakhstan (Alatai Pass Kurchumskiy Ridge) in 2001. *Tulipadianaeverettiae* is distinguishable from other closely related species (*T.biflora*, *T.kolbintsevii* and *T.patens*) by the presence of short hairs on the stem, particularly on the leaf margin, and by a distinct capsule with a small dome at the apex. The authors reported that this is the only species of the sect. Biflores growing at an altitude of 1800 m a.s.l. in the Altai Mountains. The flowers of *T.dianaeverettiae* have a sweet odor (de Groot and Zonneveld 2020). Currently, little information is available on the distribution and population status of this species.

**Figure 18.**
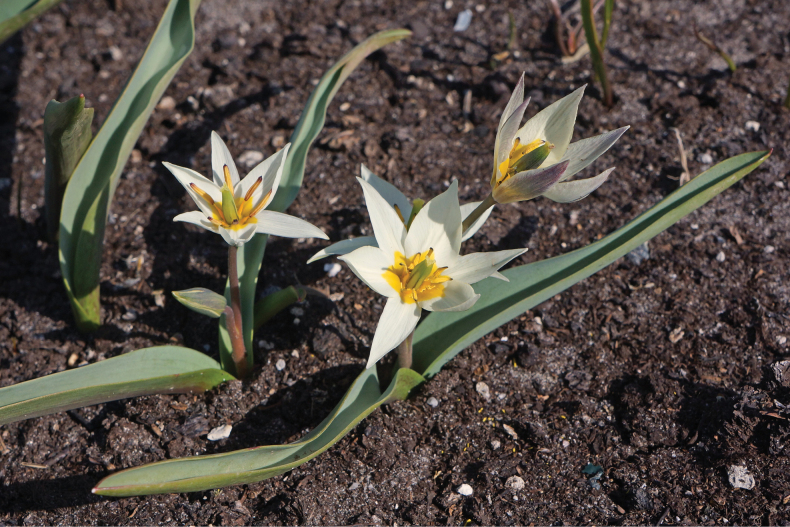
*Tulipadianae-verettiae* in Kazakhstan. (Photo by J.J. de Groot).

#### ﻿ Tulipa dubia

Taxon classificationPlantaeLilialesLiliaceae

Vved., Byull. Sredne-Aziatsk. Gosud. Univ. 21: 148 (1935).

C5101DF7-CB95-56FF-8F11-F8B9934605DA

Fig. 19

##### Type.

Tashkent district • Chotan river gorge, slope near the confluence of Kashka-su brook into Chotan, 10 June 1909, *Z. von Minkwitz* (holotype LE00053022!).

##### General distribution.

Kazakhstan, Kyrgyzstan, and Uzbekistan (POWO 2024).

##### Distribution in Kazakhstan and habitat.

Western Tian Shan. This species grows on fine-grained and rubbly-small-grained slopes in the upper belt of the mountains.

##### Conservation status.

*Tulipadubia* is an assessed near threatened species at the global level (IUCN 2024).

##### Phenology.

Flowering in April–June; fruiting in May–August (depending on the elevation).

##### Notes.

*Tulipadubia* was described by A.I. Vvedensky in 1935 from herbarium material collected by Z. Minkwitz in 1909 from the Tashkent district, Chotan River gorge. Typically, *T.dubia* populations occur in higher altitudinal zones than *T.kaufmanniana* and *T.tschimganica*. However, all three species grow at similar elevations but occupy different habitats in the Aksay Valley (Greater Chimgan). The main characteristic distinguishing *T.dubia* from the other species of Tulipasect.Spiranthera is the shape of the stamen filaments and anthers (Tojibaev et al. 2022). This species forms spontaneous hybrids with *T.kaufmanniana* in common habitats (Vvedensky 1935). The main range of this species is Uzbekistan with a few populations in Western Tian Shan. In addition, high genetic variability has been reported in *T.greigii* in Kazakhstan (Yermagambetova et al. 2024).

**Figure 19.**
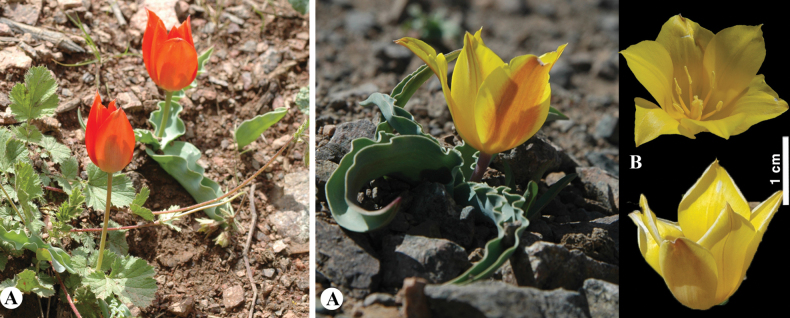
*Tulipadubia* in Uzbekistan **A** general habits **B** flowers (Photos: A–B by K. Tojibaev).

#### ﻿ Tulipa greigii

Taxon classificationPlantaeLilialesLiliaceae

Regel, Gartenflora 290: 773 (1873)

AE64B8B3-9AA0-5470-A5CF-9A5900D079D3

Fig. 20

##### Type.

• Illustration t. 773 in Gartenflora 22 (1873) [lectotype designated by Christenhusz et al. 2013: 312].

##### General distribution.

Kazakhstan, Kyrgyzstan, Tajikistan, and Uzbekistan (Tojibaev et al. 2022).

##### Distribution in Kazakhstan and habitat.

Chu-Ili Range, Karatau, Kyrgyz Alatau, Moiynkum, Trans-Ili Kungey Alatau, Turkestan, and Western Tian Shan. This species grows on clay and rubbly slopes up to 2400 m a.s.l., as well as on foothill plains and mountain flutes.

##### Conservation status.

*Tulipagreigii* is a least concern species at the global level (IUCN 2024). It is included in the Red Book of Kazakhstan (Category III).

##### Phenology.

Flowering in April–June; fruiting in June–July.

##### Notes.

*Tulipagreigii* was described by E.L. Regel in 1873 from the Karatau Mountains, based on herbarium collections of A. Sivertsev and B. Fedtschenko, initially as a variety of T.altaicavar.karatavica Regel. In the same year, it was described by the same author as an independent species (Ivashchenko and Belyalov 2019; Tojibaev et al. 2022). The species is named in honor of S.A. Greig, president of the Russian Botanical Society of Gardeners. It contains unusual speckles on its leaves that serve as the progenitors of hundreds of tulip varieties. This species often hybridizes with *T.kaufmanniana* and *T.alberti* in nature (Ivashchenko and Belyalov 2019). Vvedensky (1935) noted that the collection of this species from Syrdarya deserves a separate study as it differs from the northern (typical) *T.greigii* in that it has longer pedicels and gradually decreasing leaves at the top. In our opinion, this population represents the ecological variability in this species.

**Figure 20.**
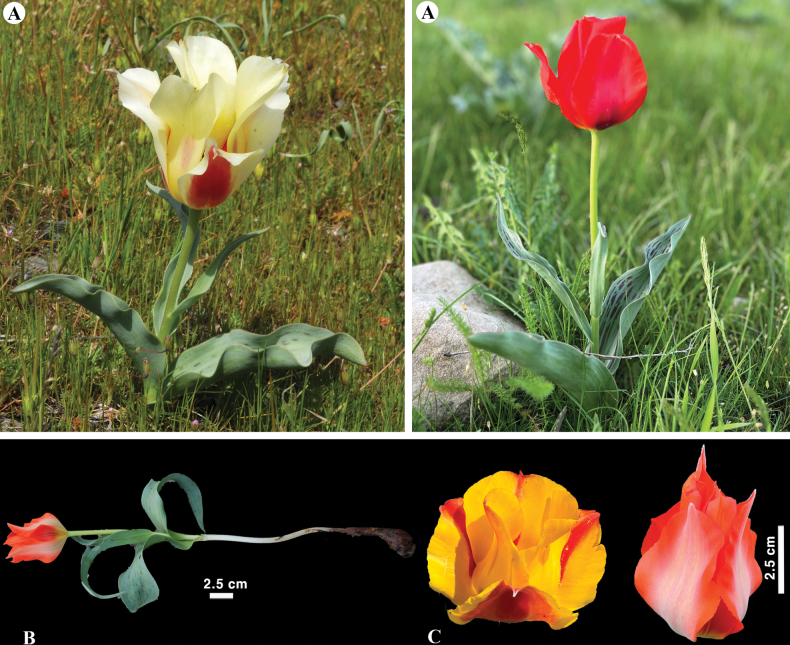
*Tulipagreigii* in Kazakhstan **A, B** general habits with different morphological form **C** flowers with different color. (Photos: **A** by V. Epiktetov, **B, C** by S. Kubentayev).

#### ﻿ Tulipa heteropetala

Taxon classificationPlantaeLilialesLiliaceae

Ledeb., Icon. Pl. [Ledebour] 1: 21, t. 85 (1829).

F363DC50-7F6D-5547-9584-47B7A97C1EBA

Fig. 21

##### Type.

Kazakhstan • ‘Bukhtarminsk et Mont Kurtschum’, *Ledebour* (holotype LE).

##### General distribution.

China (Xinjiang), Kazakhstan, and Russia (Altai) (POWO 2024).

##### Distribution in Kazakhstan and habitat.

Altai, Tarbagatai, and Zaysan. This species grows on steppes and semi-deserts on stony and rubbly slopes.

##### Conservation status.

*Tulipaheteropetala* is a least concern species at the global level (IUCN 2024). It is included in the Red Book of Kazakhstan (Category II).

##### Phenology.

Flowering in April–May; fruiting in June–July.

##### Notes.

*Tulipaheteropetala* was described by C.F. Ledebour in 1829 using herbarium material from the vicinity of Bukhtarma (East Kazakhstan). This species is morphologically similar to *T.uniflora* but different by its strong spreading, deviated leaves, very sharp tepals, and expanded stamen filaments below the middle (Vvedensky 1935). Some authors (Mordak 1990, 1992; Cherepanov 1995) consider *T.heteropetala* a synonym of *T.uniflora*. However, most authors consider it an independent species (Vvedensky 1935; Abdulina 1999; Zonneveld 2009; Christenhusz et al. 2013; Everett et al. 2013; Ivashchenko and Belyalov 2019). According to recent morphological analyses of Tulipa species of the subgenus Orithyia, *T.heteropetala* does not grow in South Siberia, and the previously reported tulips in this region, under the name *T.heteropetala* are large individuals of *T.uniflora* (Chernysheva et al. 2023).

**Figure 21.**
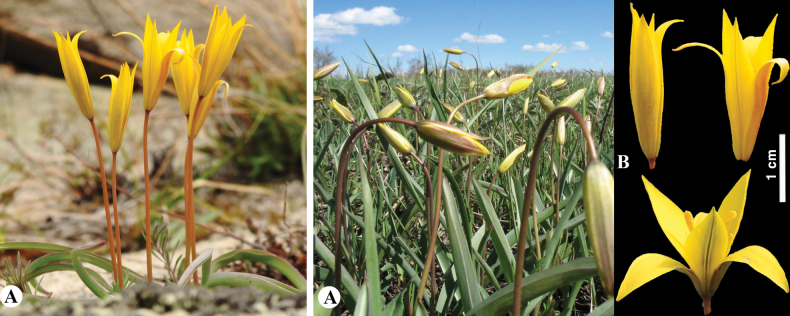
*Tulipaheteropetala* in Kazakhstan **A** general habits **B** flowers. (Photos: **A, B** by G. Bolbotov and V. Kolbintsev).

#### ﻿ Tulipa heterophylla

Taxon classificationPlantaeLilialesLiliaceae

Baker, J. Linn. Soc., Bot. 14: 295 (1874).

B98A6933-AF07-5BBA-B9CC-0206FE386F3F

Fig. 22

##### Type.

China • ‘Tianshan: Trens Ui Ala-Tau’, *Semenow* (holotype LE).

##### General distribution.

Kazakhstan, Kyrgyzstan, and China (Xinjiang) (POWO 2024).

##### Distribution in Kazakhstan and habitat.

Trans-Ili Kungey Alatau, Ketmen Terskey Alatau. This species grows on gravelly slopes, forest clearings, and subalpine meadows.

##### Conservation status.

*Tulipaheterophylla* is a least concern species at the global level (IUCN 2024). It is included in the Red Book of Kazakhstan (Category II).

##### Phenology.

Flowering in April– July; fruiting in June–August (depending on the elevation).

##### Notes.

*Tulipaheterophylla* was initially described by E.L. Regel in 1868 from Zailiyskiy Alatau as *Orithyiaheterophylla* Regel. In 1874, Baker assigned this species to the genus *Tulipa*. Notably, M.G. Popov allocated *T.heterophylla* to a new genus, *Eduardoregelia* Popov, in 1936 due to its unusual flower appearance (Ivashchenko and Belyalov 2019). This species is distinguished from other species of the subgenus Orithyia by the fact that its flower stalks are curved at the top of the stem resulting in flowers in a more or less horizontal position (Zonneveld 2009; Everett et al. 2013). More recently, *T.heterophylla* was found to have a lower level of intrachromosomal asymmetry than other species of the subgenus Orithyia (Chernysheva et al. 2023). The main range of this species does not extend beyond the Tian Shan.

**Figure 22.**
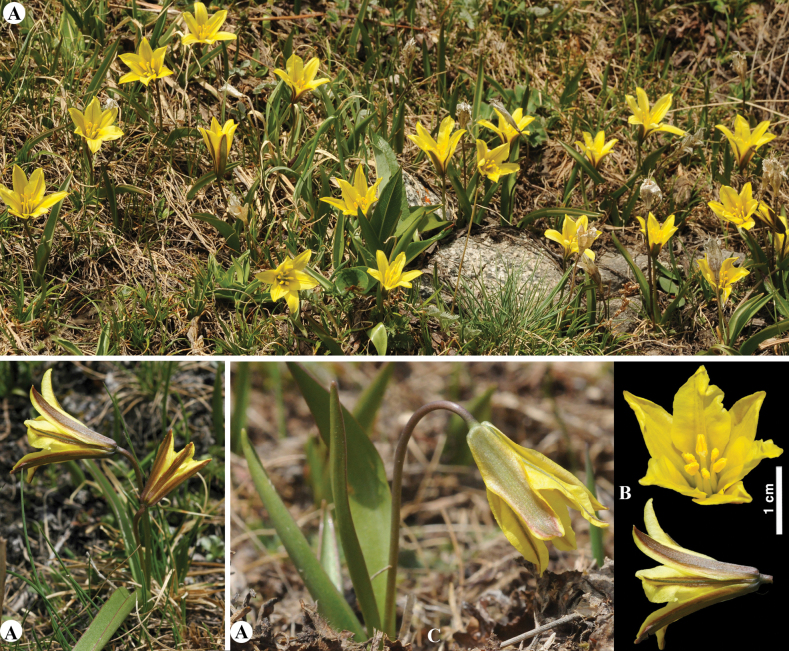
*Tulipaheterophylla* in Kazakhstan **A** general habits **B** flowers. (Photos: **A, B** by V. Epiktetov).

#### ﻿ Tulipa iliensis

Taxon classificationPlantaeLilialesLiliaceae

Regel, Gartenflora 28: 162 (1879).

0E62ABB6-2E47-5341-8B77-AC97810086CD

Fig. 23

##### Type.

Kyrgyzstan. ‘Sarybulak’, 23 Apr 1878, *A. Regel* (lectotype P-00730916; isolectotype BM) [lectotype designated by Christenhusz et al. 2013: 315].

##### General distribution.

Kazakhstan, Kyrgyzstan, and China (NW-Xinjiang) (POWO 2024).

##### Distribution in Kazakhstan and habitat.

Ketmen, and Terskey Alatau. This species grows on steppe slopes and among shrubs in the altitude range of 1300–1500 m a.s.l.

##### Conservation status.

*Tulipailiensis* is near threatened species at the global level (IUCN 2024).

##### Phenology.

Flowering in April–May; fruiting in June–July.

##### Notes.

*Tulipailiensis* was described by E.L. Regel in 1879 based on collections by A. Regel from the upper reaches of the Ile River. This species is morphologically similar to *T.altaica*, differing by the appressed hair-like pubescence at the apex and base of the bulb sheaths and linear (linear-lanceolate) leaves with a flat margin (Vvedensky 1935). Van Raamsdonk and De Vries (1995) listed *T.iliensis* and *T.kolpakowskiana* as synonyms of *T.altaica*. However, this species is recognized as independent according to the latest classifications of the genus *Tulipa* (Zonneveld 2009; Christenhusz et al. 2013; Everett et al. 2013). *Tulipailiensis*, *T.kolpakowskiana*, *T.altaica*, and *T.thianschanica* are closely related species, as confirmed by molecular genetic data (Christenhusz et al. 2013; Li et al. 2021). The main range of *T.iliensis* is in China (Xinjiang) (Qin et al. 2024). In Kazakhstan, only a few localities of this species are known on the Ketmen Terskey Alatau.

**Figure 23.**
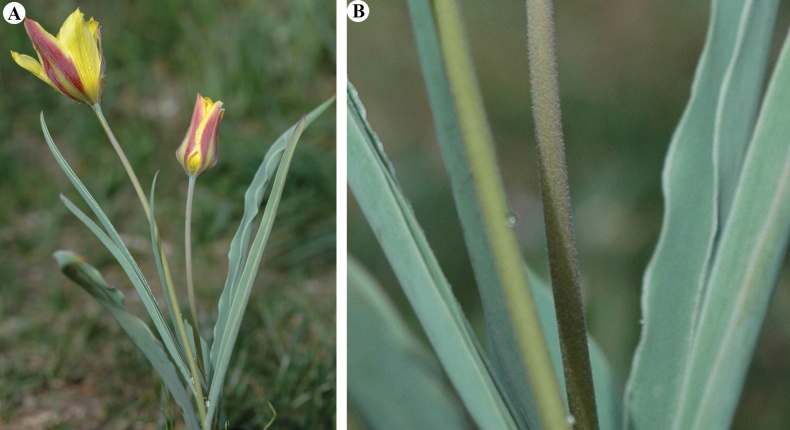
*Tulipailiensis* in Kazakhstan **A** general habits **B** stem with short pubescences and leaves with flat edge. (Photos: **A, B** by V. Epiktetov).

#### ﻿ Tulipa ivasczenkoae

Taxon classificationPlantaeLilialesLiliaceae

Epiktetov & Belyalov, Turczaninowia 16: 5 (2013).

AC7D5407-ADD1-5584-8402-8B664244AFD0

Fig. 24

##### Type.

Kazakhstan • SW part of Dzhungarian Alatau, mts. Chulak; Aiyrkezen, upper third of stony northern slope (1100 m. a. s. l.), between bushes, 26 April 2013, *V.G. Epiktetov & O.V. Belyalov* (ALTB, iso – LE).

##### General distribution.

Endemic to Kazakhstan (Kubentayev et al. 2024).

##### Distribution in Kazakhstan and habitat.

Dzungarian Alatau. This species grows on dry stony slopes among shrubs in low-desert mountains (1100 m a.s.l.).

##### Conservation status.

*Tulipaivasczenkoae* is a critically endangered species at the global level (IUCN 2024).

##### Phenology.

Flowering in April–May; fruiting in June.

##### Notes.

*Tulipaivasczenkoae* was described in 2013 from the south-western part of the Dzungarian Alatau, Chulak Mountain, Ayyrkezen. The species was named in honor of the Kazakhstani scientist Anna Andreyevna Ivasczenko, who has been studying wild bulbous plants of Kazakhstan, including tulips, since 1963. This species is closely related to the Pamir-Alai endemic *T.fosteriana* Irw. and *T.carinata* Vved. It is distinguished from the former by keeled leaves and from the latter by blunt or short pointed rather than long-pointed perianth leaflets and larger broad leaves (Epiktetov and Belyalov 2013). This species has not been encountered in other areas since its description; thus far, it is known only from its type locality.

**Figure 24.**
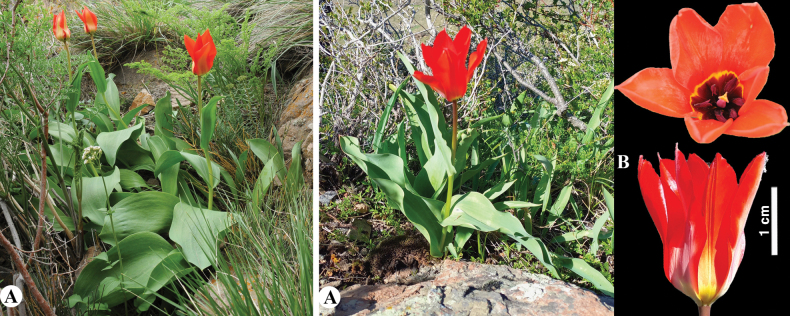
*Tulipaivasczenkoae* in Kazakhstan **A** general habits **B** flowers. (Photos: **A, B** by V. Epiktetov).

#### ﻿ Tulipa jansii

Taxon classificationPlantaeLilialesLiliaceae

J.J.de Groot & Zonn., Int. Rock Gard. 168: 1 (2024).

07156501-204C-5E05-9B7A-34ECA1FF2FAF

Fig. 25

##### Type.

• Wild collected material from the Ily Valley north of Kapchagay. *J.J. de Groot and B.J.M. Zonneveld* (holotype L4513065).

##### General distribution.

Endemic to Kazakhstan (de Groot and Zonneveld 2024).

##### Distribution in Kazakhstan and habitat.

Balkhash-Alakol and Dzungarian Alatau. This species grows on dry, stony slopes at the foot of mountains.

##### Conservation status.

The IUCN conservation status of this species requires assessment.

##### Phenology.

Flowering in April–May; fruiting in June.

##### Notes.

*T.jansii* was recently described from the Ili River valley north of Kapchagai (de Groot and Zonneveld 2024). This species was named after Harry Jans, a famous world traveler. The main difference between *T.jansii* and other representatives of T.sect.Kolpakowskianae is the pear-shaped bulb with stolons, non-elongated tunic without fibers, narrower lanceolate leaves and narrow, almost straight filaments (de Groot and Zonneveld 2024).

**Figure 25.**
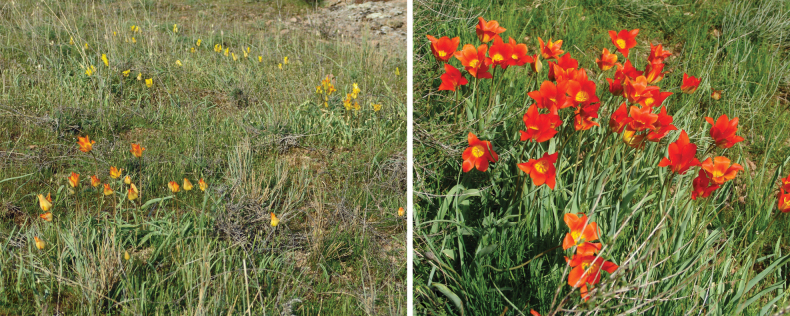
*Tulipajansii* in Kazakhstan, general habits. (Photos by J.J. de Groot).

#### ﻿ Tulipa kaufmanniana

Taxon classificationPlantaeLilialesLiliaceae

Regel, Gartenflora 26: 194 (1877).

3DFAC912-F283-50F0-93F7-7058BCBDF982

Fig. 26

##### Type.

Uzbekistan • ‘In Turkestaniae montibus fluvium Tschirtschik [Chirchiq] adjacentibus’, *A. Regel* (LE!).

##### General distribution.

Kazakhstan, Kyrgyzstan, Tajikistan, and Uzbekistan (POWO 2024).

##### Distribution in Kazakhstan and habitat.

Western Tian Shan, Karatau, and Kyrgyz Alatau. This species grows on shaded slopes, meadow areas, and scrub thickets and less often on rocky slopes from the lower to the upper mountain belts.

##### Conservation status.

*Tulipakaufmanniana* is a near threatened species at the global level (IUCN 2024). It is included in the Red Book of Kazakhstan (Category III).

##### Phenology.

Flowering in March–May; fruiting in June–July.

##### Notes.

*Tulipakaufmanniana* was described by E.L. Regel in 1877 from the Chirchik River basin. The species was named in honor of Kaufmann, Governor-General of the Turkestan region (Ivashchenko and Belyalov 2019). *Tulipaberkariensis* Rukšāns, described from the Berkari Gorge by Rukšāns (2019), differs from *T.kaufmanniana* by the globular bulb shape and the presence of horizontal stolons. Currently, many researchers consider *T.berkariensis* a synonym of *T.kaufmanniana* (Christenhusz et al. 2013; Everett et al. 2013; Sennikov and Tojibaev 2021). However, *T.berkariensis* has a slightly smaller genome size than the authentic *T.kaufmanniana* from Uzbekistan (Zonneveld 2009). Further research is needed to study *T.berkariensis* and establish its taxonomic status.

**Figure 26.**
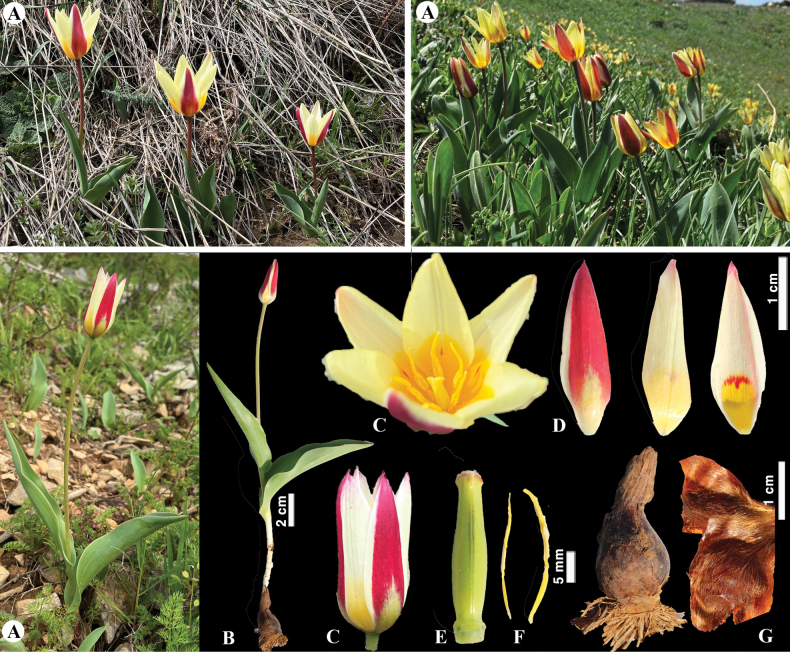
*Tulipakaufmanniana* in Kazakhstan **A, B** general habits **C** flower **D** tepals **E** gynoecium **F** stamens **G** bulb and bulb sheath. (Photos: **A** by S. Kubentayev and V. Epiktetov, **B–G** by S. Kubentayev).

#### ﻿ Tulipa kolbintsevii

Taxon classificationPlantaeLilialesLiliaceae

Zonn., Pl. Syst. Evol. 298: 1294 (2012).

0980F1E3-D12C-599D-AC9F-573009C86873

Fig. 27

##### Type.

Kazakhstan • Grown in the Netherlands from material collected at Dzjungarian Ala-Tau, Taskora Valley, cult. *J.J. de Groot* (holotype L 0821329!).

##### General distribution.

Endemic to Kazakhstan (Kubentayev et al. 2024).

##### Distribution in Kazakhstan and habitat.

Dzungarian Alatau. This species grows among shrubs at an altitude of 650 m a.s.l.

##### Conservation status.

*Tulipakolbintsevii* is an endangered species at the global level (IUCN 2024).

##### Phenology.

Flowering in March–June; fruiting in May–June.

##### Notes.

*Tulipakolbintsevii* was described from the cultural collection of J.J. de Groot in the Netherlands, collected from Taskora Valley, 650 m from Dzjungarian Ala-Tau, Kazakhstan (Zonneveld and de Groot 2012). The main difference between *T.kolbintsevii* and other Tulipa species in the section Biflores is that the sepals have a pilose margin at the base rather than a pilose band. This species was named in honor of V. Kolbintsev, who guided the expedition leading to discovery (Zonneveld and de Groot 2012). *Tulipakolbintsevii* is diploid, with a 48.0 pg genome size, smaller than that of other species in T.sect.Biflores, ranging from 51.5 to 76.3 pg (Zonneveld 2009). The distribution of this species is poorly studied, and known from only two localities, including “locus classicus”.

**Figure 27.**
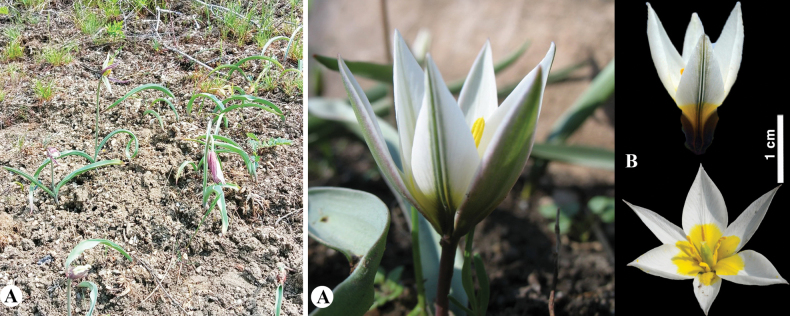
*Tulipakolbentsevii* in Kazakhstan **A** general habits **B** flowers. (Photos: **A, B** by Zh. Nurgozhanova and V. Epiktetov).

#### ﻿ Tulipa kolpakowskiana

Taxon classificationPlantaeLilialesLiliaceae

Regel, Trudy Imp. S. Peterburgsk. Bot. Sada 5: 266 (1877).

A5618EF8-51F3-5218-AFC2-1FFFE00E08A5

Fig. 28

##### Type.

Kazakhstan • ‘In Turkestania prope Verniy et in valle fluvii Almatinka’, *A. Regel* (holotype LE; possible isotype K).

##### General distribution.

Afghanistan, Kazakhstan, Kyrgyzstan, and China (Xinjiang) (POWO 2024).

##### Distribution in Kazakhstan and habitat.

Trans-Ili Kungey Alatau, Moiynkum, Chu-Ili range, Dzungarian Alatau, Kyrgyz Alatau, and Ketmen Terskey Alatau. This species grows on clayey, less often gravelly, slopes of steppe and desert foothills.

**Figure 28.**
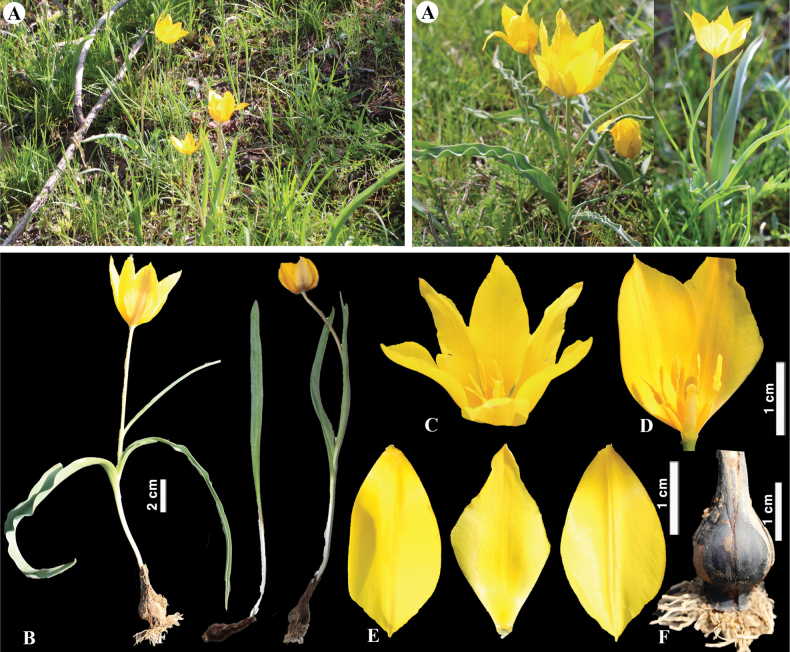
*Tulipakolpakowskiana* in Kazakhstan **A, B** general habits **C, D** flower **E** tepals **F** bulb. (Photos: **A–F** by S. Kubentayev).

##### Conservation status.

*Tulipakolpakowskiana* is a near threatened species at the global level (IUCN 2024). It is included in the red book of Kazakhstan (Category III).

##### Phenology.

Flowering in April–May; fruiting in June–July.

##### Notes.

*Tulipakolpakowskiana* was first described by E.L. Regel in 1877 from Verniy vicinity (Almaty). This species was named in honor of G. A. Kolpakowskiy, an honorary member of the Russian Geographical Society (Ivashchenko and Belyalov 2019). Van Raamsdonk et al. (1997) classified this species as a synonym of T.altaicavaraltaica. However, after studying wild material, Zonneveld (2009), distinguished it as a separate species of the section Kolpakowskianae (Everett et al. 2013). In nature, this species often hybridizes with *T.ostrowskiana*, *T.tetraphylla*, and *T.brachystemon* (Vvedensky 1935; Ivashchenko and Belyalov 2019).

#### ﻿ Tulipa korolkowii

Taxon classificationPlantaeLilialesLiliaceae

Regel, Trudy Imp. S. Peterburgsk. Bot. Sada 3: 295 (1875).

3332DC4A-E3BC-5FD1-9D42-D69E14B69374

Fig. 29

##### Type.

Uzbekistan • ‘Habitat in solo lutoso in desertis inter Turkestaniam et Khiwam prope Farisch’, *Korolkow et Krause* (holotype LE!).

##### General distribution.

Kazakhstan, Kyrgyzstan, Tajikistan, Turkmenistan, and Uzbekistan (Tojibaev et al. 2022).

##### Distribution in Kazakhstan and habitat.

Turkestan, and Western Tian Shan. This species grows on dry gravel and stony surfaces, sandy ceilings, slopes, and plains at the outlet (opening), and in the lower belt of the mountain (up to 1800 m a.s.l.).

**Figure 29.**
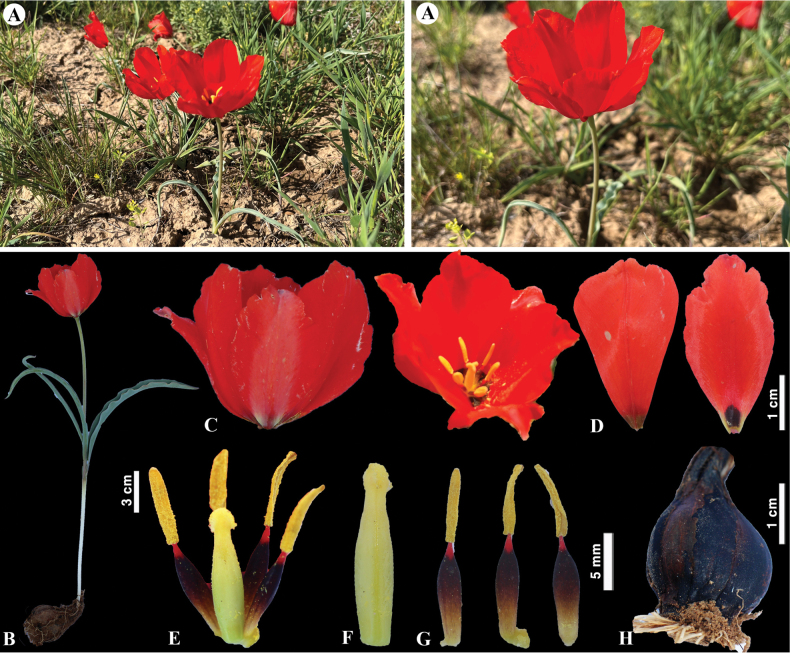
*Tulipakorolkowii* in Kazakhstan **A, B** general habits **C** flower **D** tepals **E** gynoecium and stamens **F** gynoecium **G** stamens **H** bulb. (Photos: **A–H** by S. Kubentayev).

##### Conservation status.

*Tulipakorolkowii* is a near threatened species at the global level (IUCN 2024). It is included in the Red Book of Kazakhstan (Category II).

##### Phenology.

Flowering in March–April; fruiting in May–June.

##### Notes.

*Tulipakorolkowii* was first described by E.L. Regel in 1875 from Forish (Uzbekistan). This species was named in honor of amateur botanist N. I. Korolkow, who lived and worked in the Turkestan region from 1868 to 1905 (Ivashchenko and Belyalov 2019). Vvedensky (1935) distinguished *T.korolkowii* and two related species, *T.rosea* Vved. and *T.nitida* Hoog, as geographically isolated species (Tojibaev et al. 2022). Based on the flow cytometry results, Zonneveld (2009) considered *T.korolkowii* and *T.nitida* two different species and *T.rosea* as T.korolkowiif.rosea (Vvedensky). Christenhusz et al. (2013) considered *T.nitida* and *T.rosea* as synonyms of *T.korolkowii*. Based on long-term studies in natural habitats and observations in *ex situ* living collections in TASH and Tashkent Botanical Garden, Tojibaev et al. (2022) found that morphological characters between *T.korolkowii* and *T.nitida*, vary depending on habitat and climatic conditions. In addition, they did not find any evidence to confirm the presence of *T.rosea* within the present-day boundaries of Uzbekistan.

#### ﻿ Tulipa kujukense

Taxon classificationPlantaeLilialesLiliaceae

J.J. de Groot & Zonn.

AA1983D9-DA71-5EFA-9D4C-4D6E1FB8E370

Fig. 30

##### Type.

Kazakhstan • Vegetative progeny of wild collected material grown in the collection of *J.J. de Groot*, collection number G05-8 (holotype L4513067).

##### General distribution.

Endemic to Kazakhstan (de Groot and Zonneveld 2024).

##### Distribution in Kazakhstan and habitat.

Karatau. This species grows among shrubs.

##### Conservation status.

The IUCN conservation status of this species requires assessment.

##### Phenology.

Flowering in March–April; fruiting in May–June.

##### Notes.

*Tulipakujukense* was first described in 2024 from Karatau (Kuyuk Pass). *Tulipakujukense* is morphologically similar to *T.orthopoda* and *T.bifloriformis* but differs in size and by bulb with gray-brown tunica, characteristic of the Turkestanica species group. In addition, *T.kujukense* has a large genome size (60.7 pg) compared with other species of T.sect.Biflores (de Groot and Zonneveld 2024).

**Figure 30.**
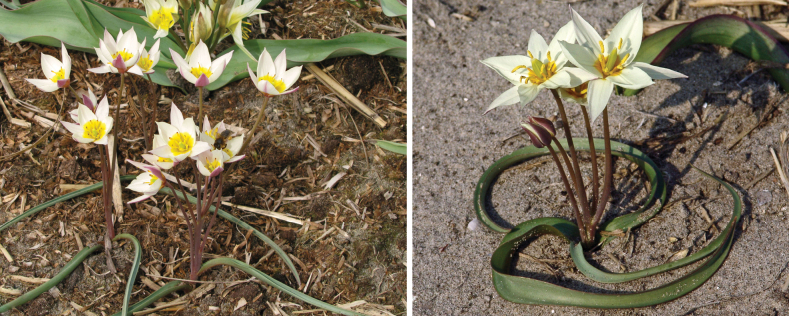
*Tulipakujukense* in Kazakhstan, general habits. (Photo by J.J. de Groot).

#### ﻿ Tulipa lehmanniana

Taxon classificationPlantaeLilialesLiliaceae

Merckl., A.A.von Bunge, Beitr. Fl. Russl. 7: 337 (1852).

D77BD137-CF9A-596C-AFCC-9FB162F5DA58

Fig. 31

##### Type.

Uzbekistan • *A. Lehmann sn*., *Bunge Rel. Lehm. 337* (K-000844622) [lectotype designated by Christenhusz et al. 2013: 316].

##### General distribution.

Afghanistan, Eastern Iran, Kazakhstan, Kyrgyzstan, Tajikistan, Turkmenistan, and Uzbekistan (POWO 2024).

##### Distribution in Kazakhstan and habitat.

Balkhash-Alakol, Betpak-Dala, Chu-Ili range, Kyzylkum, Moiynkum, Turkestan, and Western Tian Shan. This species grows on sand and variegated rock outcrops, in sandy and stony deserts.

**Figure 31.**
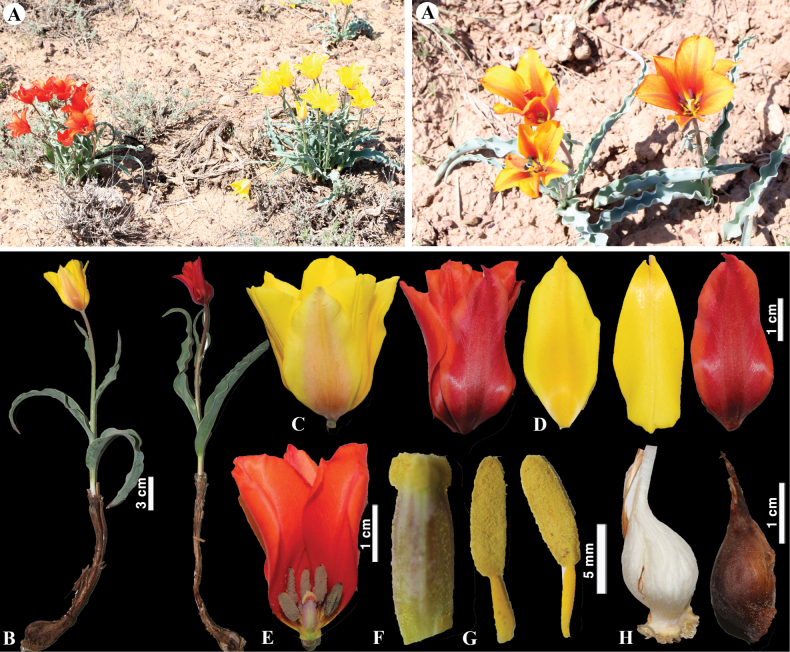
*Tulipalehmanniana* in Kazakhstan **A, B** general habits **C, E** flowers with different color variants **D** tepals of different color variants **F** gynoecium **G** stamens **H** bulb (Photos: **A–H** by S. Kubentayev).

##### Conservation status.

*Tulipalehmanniana* is a near threatened species at the global level (IUCN 2024). It is included in the Red Book of Kazakhstan (Category I).

##### Phenology.

Flowering in April; fruiting in May–June.

##### Notes.

*Tulipalehmanniana* was described by Merklin in 1854 from collections made near Bukhara. The species was named in honor of A. Lehmann, a Russian botanist, who collected plants from Central Asia on behalf of the St. Petersburg Botanical Garden (Ivashchenko and Belyalov 2019). Christenhusz et al. (2013) considered *T.zenaidae* a synonym of *T.lehmanniana*, which we strongly disagree with. These species are distinct morphologically, ecologically, and geographically. Eduard Ludvigovich Regel described *T.behmiana* Regel in 1880 from the vicinity of the Iliysk settlement (Kapchagai). This species was classified as a synonym of *T.lehmanniana* according to the latter classifications (Van Raamsdonk and De Vries 1995; Zonneveld 2009; Christenhusz et al. 2013; Everett et al. 2013).

#### ﻿ Tulipa lemmersii

Taxon classificationPlantaeLilialesLiliaceae

Zonn., Peterse & J.de Groot, Pl. Syst. Evol. 298: 91 (2012).

C0DCE3EF-73FC-5567-A152-28975A679A0A

Fig. 32

##### Type.

Kazakhstan • Chimkent: Mashad Pass, cult. *A. Peterse* (holotype L 0822655).

##### General distribution.

Endemic to Kazakhstan (Kubentayev et al. 2024).

##### Distribution in Kazakhstan and habitat.

Western Tian Shan. This species grows on dry stony slopes with outcrops or shallow deposits of conglomerate rocks, on a plateau or canyon sites.

##### Conservation status.

*Tulipalemmersii* is a vulnerable species at the global level (IUCN 2024).

##### Phenology.

Flowering in March–April; fruiting in May.

##### Notes.

*Tulipalemmersii* was first found in 2007 by A. Peterse, at the top of steep cliffs on Mashat Pass, when he accompanied a tulip expedition organized by W. Lemmers (the species is named in his honor). *Tulipalemmersii* can be distinguished from *T.iliensis*, *T.ferganica*, *T.anisophylla*, and *T.tetraphylla* by its glabrous stems, thin tunic, and single flowers. It can be distinguished from other species of the T.sect.Kolpakowskianae as they have mostly red and yellow flowers. It is a diploid species with the smallest genome size (36 pg) among all species in the section Kolpakowskianae (Zonneveld 2009). The species was later validated using type designation in 2012 (Veldkamp and Zonneveld 2012).

**Figure 32.**
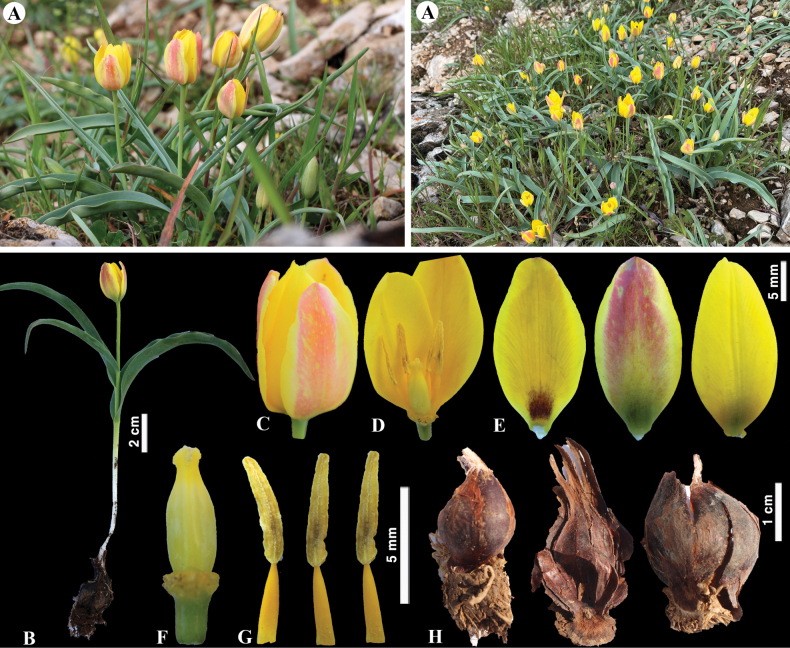
*Tulipalemmersii* in Kazakhstan **A, B** general habits **C, D** flower **E** tepals **F** gynoecium **G** stamens **H** bulb and bulb sheath. (Photos: **A–H** by S. Kubentayev).

#### ﻿ Tulipa orthopoda

Taxon classificationPlantaeLilialesLiliaceae

Vved., Opred. Rast. Sred. Azii 2: 320 (1971).

25285871-BE2A-5A04-A611-FB5E8E031411

Fig. 33

##### Type.

Kazakhstan • Turkestan, 5 April 1930, *Lipschitz & Pavlov 32* (holotype TASH!).

##### General distribution.

Endemic to Kazakhstan (Kubentayev et al. 2024).

##### Distribution in Kazakhstan and habitat.

Karatau, Western Tian Shan. This species grows in clay, gravel and stony slopes of the lowlands.

##### Conservation status.

*Tulipaorthopoda* is a vulnerable species at the global level (IUCN 2024).

##### Phenology.

Flowering in March–April; fruiting in May–June.

##### Notes.

*Tulipaorthopoda* is a narrowly localized endemic species to Kazakhstan (Kamelin 1990). This species was first mentioned by Vvedensky (1935) in Flora of the USSR in a note on *T.bifloriformis* Tulip specimens from Karatau are reportedly characterized by drooping buds, lower height, and more pubescent leaves. A detailed description in Latin was first published in 1971 in the Conspectus Florae Asiae Mediae (Vvedensky and Kovalevskaya 1971). Christenhusz et al. (2013) reported that *T.orthopoda* is a synonym of *T.bifloriformis*; however, later indicated that it deserves recognition. According to Zonneveld (2009), the genome size of *T.orthopoda* is 59.3 pg, while that of *T.bifloriformis* is 56.6 pg. This difference was sufficient to distinguish between two representatives of the same group (Everett et al. 2013). Wilson (2023) listed *T.orthopoda* as an independent taxon in his updated list of recognized tulip species. We agree that *T.orthopoda* should be recognized as an independent taxon based on several morphological characteristics and different flowering times (*T.orthopoda* flowers earlier than *T.bifloriformis*).

**Figure 33.**
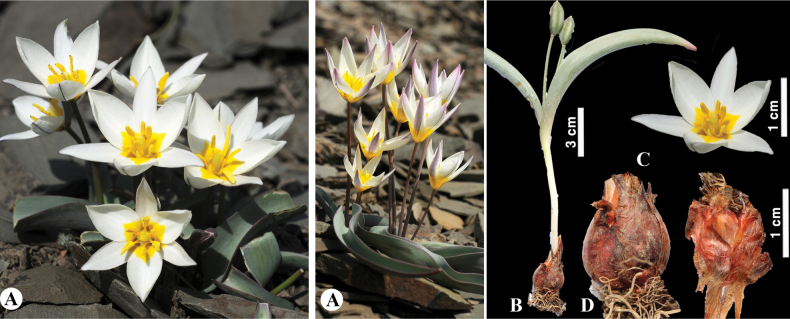
*Tulipaorthopoda* in Kazakhstan **A, B** general habits **C** flower **D** bulb and bulb sheath. (Photos: **A, C** by V. Epiktetov; **B, D** by S. Kubentayev).

#### ﻿ Tulipa ostrowskiana

Taxon classificationPlantaeLilialesLiliaceae

Regel, Gartenflora 33: 34 (1884).

DDE010C2-97E5-5BA8-9E60-BA8C41AA28B5

Fig. 34

##### Type.

Kazakhstan • ‘Iter Turkestanicum, Kl. Almaty Schlucht bei Werny’, 2 April 1879, *A. Regel* (K).

##### General distribution.

Kazakhstan, and Kyrgyzstan (POWO 2024).

##### Distribution in Kazakhstan and habitat.

Kyrgyz Alatau and Trans-Ili Kungey Alatau. This species grows on slopes with deep nutritious soil (less often gravelly), in the lower and middle mountain belts.

##### Conservation status.

*Tulipaostrowskiana* is assessed near threatened at the global level (IUCN 2024). It is included in the Red Book of Kazakhstan (Category III).

##### Phenology.

Flowering in April– May; fruiting in June– July.

##### Notes.

*Tulipaostrowskiana* was described in 1884 by E.L. Regel from collections near Verny (Almaty). This species was first collected in 1879 by A.E. Regel and A.M. Fetisov (the chief gardener of Pishpek (Bishkek) in charge of the Treasury Garden in Verny) (Ivashchenko and Belyalov 2019). Zonneveld (2009) suggested that *T.ostrowskiana* may be an allotetraploid originating from *T.kolpakowskiana* and *T.lemmersii* based on genome size. Spontaneous hybrids of *T.ostrowskiana* and *T.kolpakowskiana* with intermediate flower coloration and habit traits are known in nature (Vvedensky 1935; Ivashchenko and Belyalov 2019).

**Figure 34.**
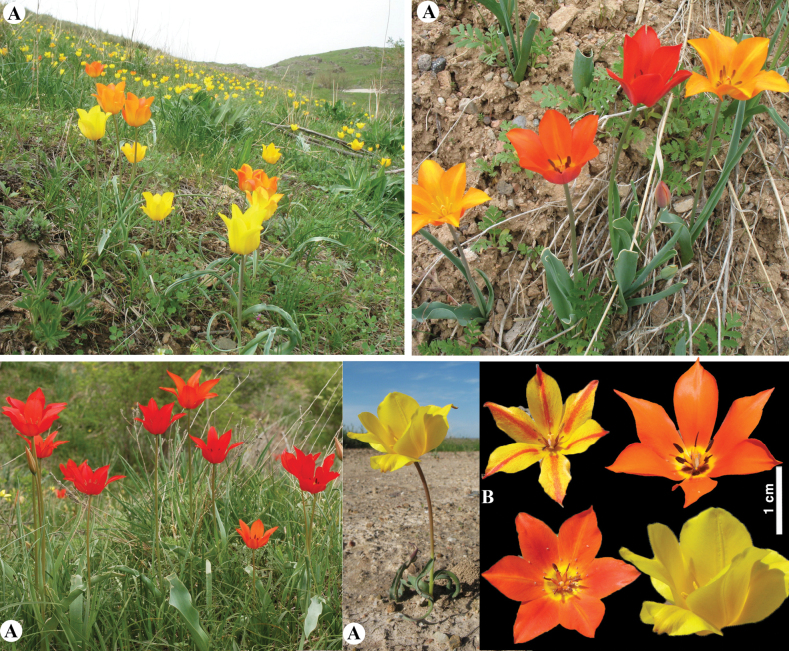
*Tulipaostrowskiana* in Kazakhstan **A** general habits **B** flowers showing different color morphs. (Photos: **A, B** by V. Kolbintsev and V. Epiktetov).

#### ﻿ Tulipa patens

Taxon classificationPlantaeLilialesLiliaceae

C.Agardh, Syst. Veg., ed. 15 bis [Roemer & Schultes] 7: 384 (1829).

E8340256-2A81-5040-ACC7-EEE807EC5ADF

Fig. 35

##### Type.

‘In Sibiria’, Agardh (LD?, not found).

##### General distribution.

Kazakhstan, and Russia.

##### Distribution in Kazakhstan and habitat.

Syrt, Tobol-Ishim, Irtysh, Semipalatinsk pine forest, Kokchetav, Mugojary, Turgay, Western Upland, Ulytau, Zaysan, Eastern Upland, Karkaraly, and Altai, Tarbagatai. This species grows in steppe, semi-desert and shrub land on gravelly clayey slopes.

##### Conservation status.

The IUCN conservation status of this species requires assessment. It is included in the Red Book of Kazakhstan (Category III).

##### Phenology.

Flowering in April–May; fruiting June.

##### Notes.

A complete species description of *T.patens* was published in 1829 by J. Roemer and J. Schultes (Ivashchenko and Belyalov 2019). Subsequently, C. F. Ledebour described it as *T.tricolor* Ledeb. The taxonomic position of *T.patens* is relatively controversial. Some authors (Christenhusz et al. 2013; Everett et al. 2013; Li et al. 2021) place it in synonymy of T.sylvestrissubsp.australis, while other authors (Polyakov 1958; Cherepanov 1995; Zonneveld 2009; Kutlunina et al. 2013; Wilson 2023) consider this species an independent taxon. We believe that *T.patens* deserves recognition based on its complex morphological characteristics and ecology.

**Figure 35.**
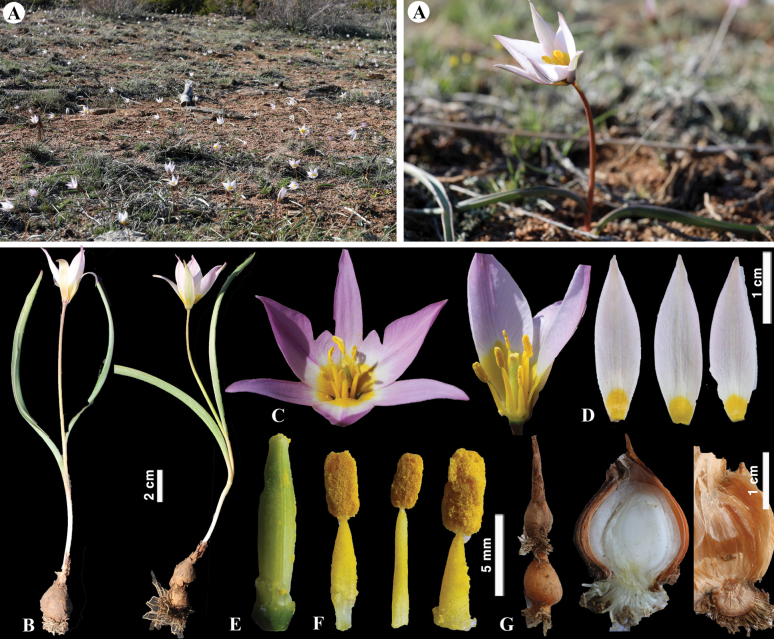
*Tulipapatens* in Kazakhstan **A, B** general habits **C** flower **D** tepals **E** gynoecium **F** stamens **G** bulb and bulb sheath. (Photos: **A–G** by S. Kubentayev).

#### ﻿ Tulipa regelii

Taxon classificationPlantaeLilialesLiliaceae

Krasn., Bot. Zap. 2: 21 (1888).

E77EDC0C-016A-5B65-97A8-D0535B8910ED

Fig. 36

##### Type.

Kazakhstan • ‘Prope fauces fluminis Kurtu inter saxa non rara in montibus Andrakai rarior’, April 1886, *A. Krassnow s.n.* (LE).

##### General distribution.

Endemic to Kazakhstan (Kubentayev et al. 2024).

##### Distribution in Kazakhstan and habitat.

Balkhash-Alakol, and Chu-Ili range. This species grows on rocky, gravelly slopes and scree (800–1100 m a.s.l.).

##### Conservation status.

*Tuliparegelii* is an endangered species at the global level (IUCN 2024). It is included in the Red Book of Kazakhstan (Category II).

##### Phenology.

Flowering in March–April; fruiting in May–June.

##### Notes.

*Tuliparegelii* was described in 1887 by the prominent Russian botanical geographer A. N. Krasnov, who surveyed the Shu-Ili Mountains (where the species was collected from the Anyrakai and Kurti tracts). The species is named in honor of E.L. Regel, the director of the botanical garden in St. Petersburg (Ivashchenko and Belyalov 2019). It is one of the most distinctive tulips due to the leaf blade surface having many parallel ridge-like outgrowths. However, the position of this species in the genus *Tulipa* remains controversial. Vvedensky (1935) classified *T.regelii* into a separate section, T.sect.Lophophyllon Vved, due to the unique structure of its leaf blade. Meanwhile, Zonneveld (2009) placed it in the section Biflores based on its genome size and flower structure. However, Christenhusz et al. (2013) assigned *T.regelii* to the subgenus Eriostemones. Later, Wilson (2023) placed *T.regelii* in the section Sylvestres (Baker) Baker.

**Figure 36.**
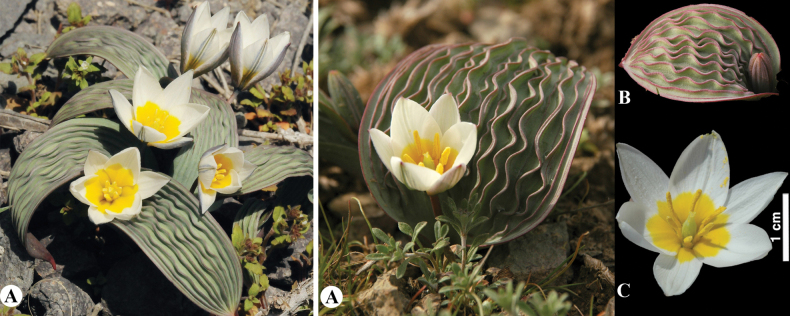
*Tuliparegelii* in Kazakhstan **A** general habits **B** leaf blade **C** flower. (Photos: **A–C** by V. Epiktetov).

#### ﻿ Tulipa salsola

Taxon classificationPlantaeLilialesLiliaceae

Rukšāns & Zubov, Int. Rock Gard. 148: 11 (2022).

3E2D081E-1AA4-5F26-83A5-D3345FA2BD61

Fig. 37

##### Type.

Kazakhstan • Zhetysu region, the extreme southern part of Dzungarian Alatau (44°10'N, 79°31'E); sandy, saline soils within semi-desert habitat, c. 880 m.a.s.l.; leg. 05 May 2012, *Rukšāns*; cult. (12KZ-059 specimen grown in J. Rukšāns garden, Latvia), fl. 11 Apr. 2021, *Rukšāns* (holotype GB).

##### General distribution.

Endemic to Kazakhstan (Kubentayev et al. 2024).

##### Distribution in Kazakhstan and habitat.

Dzungarian Alatau, and Trans-Ili Kungey Alatau. This species grows on fixed and hilly-ridged sands over brown and gray-brown soils, in depressions of takyrs and solonchaks with Saxaul.

##### Conservation status.

The IUCN conservation status of this species requires assessment.

##### Phenology.

Flowering in March–April; fruiting in May–June.

##### Notes.

*Tulipasalsola* was described relatively recently in 2022 from the southern part of Dungarian Alatau, Zhetysuyskaya Oblast, Kazakhstan. It is morphologically similar to *T.kolbintsevii* but differs by the presence of 2(–3) flowers, an adaxially strongly woolly bulb tunic, absent elongated bulb tunic, and stamens shorter or equal to the ovary (vs. one flower, naked on the neck with some hairs on the bulb tunic adaxially, elongated bulb tunic, and stamens longer than the ovary in *T.kolbintsevii*) (Rukšāns and Zubov 2022).

**Figure 37.**
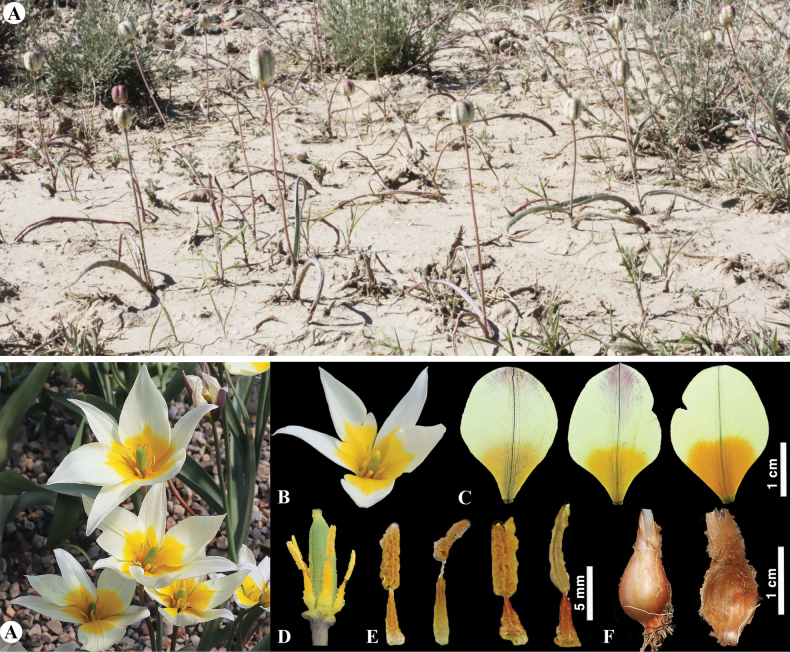
*Tulipasalsola* in Kazakhstan. **A** general habits **B** flower **C** tepals **D** gynoecium and stamens **E** stamens **F** bulb and bulb sheath. (Photos: A–F by J. Rukšāns).

#### ﻿ Tulipa sogdiana

Taxon classificationPlantaeLilialesLiliaceae

Bunge, Beitr. Fl. Russl. 338 (1852).

A139F476-B744-523C-8A48-3964BD22A857

Fig. 38

##### Type.

Uzbekistan • Inter Bukhara et Kermine, A. Lehmann *s.n.* (holotype LE; isotypes K 000844627, P00730919, P00730920).

##### General distribution.

Kazakhstan, Tajikistan, Turkmenistan, and Uzbekistan (Tojibaev et al. 2022).

##### Distribution in Kazakhstan and habitat.

Kyzylkum, Southern Ustyrt, Northern Ustyrt, and Mangyshlak. This species grows in deserts on sandy and clay soils.

##### Conservation status.

The IUCN conservation status of this species requires assessment.

##### Phenology.

Flowering in March–April; fruiting in May–June.

##### Notes.

*Tulipasogdiana* was first described in 1854 by A.A. Bunge from a desert region between Bukhara and Kermin (Uzbekistan). In “Flora of Kazakhstan” (Polyakov 1958), this species was listed in Kyzylkum. Although we did not identify herbarium materials in this area, we believe that it is in Kzyzlkum, as there are numerous confirmed localities of this species in the Uzbekistan part of Kyzylkum (Tojibaev et al. 2022). *Tulipasogdiana* differs from similar species of the subgenus Eriostemones by its glabrous filaments of stamens (Everett et al. 2013; Ivashchenko and Belyalov 2019; Tojibaev et al. 2022). Christenhusz et al. (2013) and Everett et al. (2013) placed *T.sogdiana* in synonymy of *T.biflora* s.l. However, some authors consider it an independent taxon (Abdulina 1999; Zonneveld 2009; Tojibaev et al. 2022). Wilson (2023) believed that *T.sogdiana* can be recognized if its uniqueness is proven. Additional studies of this species under natural growing conditions are required to establish its taxonomic position.

**Figure 38.**
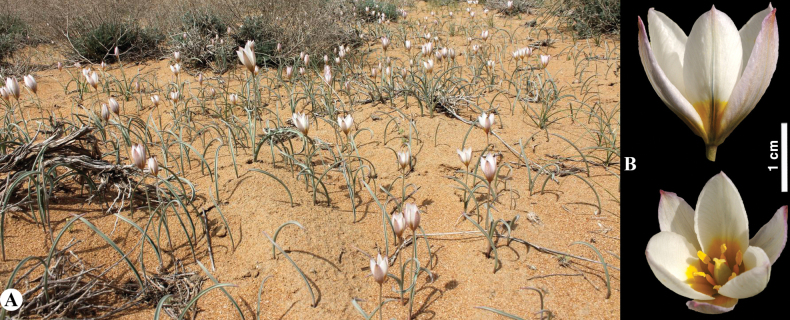
*Tulipasogdiana* in Kazakhstan **A** general habitat **B** flowers. (Photos: **A** by F. Shakula; https://www.plantarium.ru/page/image/id/766687.html); **B** by V. Epiktetov).

#### ﻿ Tulipa suaveolens

Taxon classificationPlantaeLilialesLiliaceae

Roth, Ann. Bot. (Usteri) 10: 44 (1794).

E6633ABD-0ABA-5B3A-AB29-A1A0BE9C6685

Fig. 39

##### Type.

Kazakhstan • ‘Deserta Caspica’, *Pallas* (neotype BM!) [neotype designated by Christenhusz et al. 2013: 320].

##### General distribution.

Kazakhstan, Krym, North Caucasus, and Transcaucasus (POWO 2024).

##### Distribution in Kazakhstan and habitat.

Aktobe, Turgay, Bukeev, Emba, Mugojary, Caspian region, Syrt, Tobol-Ishim, Western Upland, Aral region, and Ulytau. This species grows on steppe and semi-desert areas.

##### Conservation status.

The IUCN conservation status of this species has not yet been assessed. It is included in the Red Book of Kazakhstan (Category III).

##### Phenology.

Flowering in March–April; fruiting in May–June.

##### Notes.

This species is usually reported under *T.schrenkii* Regel, which was first described from the Yesil River valley in 1873. However, the older name *T.suaveolens* takes precedence (Christenhusz et al. 2013). Christenhusz et al. (2013) provided lectotyping of *T.suaveolens* from a specimen collected in the wild, securing the name for wild plants. In Kazakhstan, this species is found in the Caspian region to the Eastern Shallow Basin and from the northern borders of the country to the northern part of Mangystau (Ivashchenko and Belyalov 2019).

**Figure 39.**
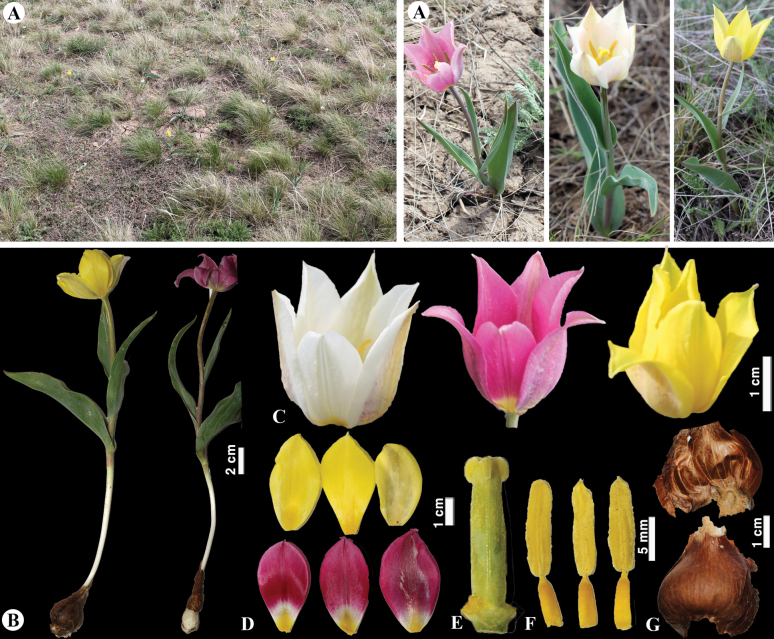
*Tulipasuaveolens* in Kazakhstan **A, B** general habits **C** flowers showing color variation of the species **D** tepals of two different color morphs **E** gynoecium **F** stamens **G** bulb and bulb sheath. (Photos: **A–G** by S. Kubentayev).

#### ﻿ Tulipa tarda

Taxon classificationPlantaeLilialesLiliaceae

Stapf, Bot. Mag. 156: t. 9321 (1933).

014394BA-6F6A-5175-8379-2154521426AF

Fig. 40

##### Type.

Cultivated • Bulbs sent to Firma Van Tubergen, originally from Iran (Urmia) in 1928. A preserved flower from the Van Tubergen garden and associated original illustration are labeled as presented by the Editor of the Botanical Magazine to the Kew Herbarium (K).

##### General distribution.

Kazakhstan, and Kyrgyzstan.

##### Distribution in Kazakhstan and habitat.

Trans-Ili Kungey Alatau. This species grows on stony-gravelly and rocky slopes, steppe areas and thickets of shrubs in the lower mountain belt.

##### Conservation status.

The IUCN conservation status of this species has not yet been assessed. It is included in the Red Book of Kazakhstan (Category II).

##### Phenology.

Flowering in April–May; fruiting June.

##### Notes.

Christenhusz et al. (2013) formally synonymized *T.tarda* into a taxon with the older name *T.urumiensis* Stapf when revising the genus. However, the name *T.tarda* has become entrenched in literature, horticultural trade, and conservation assessments, leading to nomenclatural destabilization and confusion. For these reasons, it has been suggested that the name *T.urumiensis* should be rejected so that *T.tarda* becomes the correct name for this species (Christenhusz and Wilson 2022). *Tulipatarda* is endemic in northern Tien-Shan and grows in the western part of the Zailiyskiy Alatau ridge and adjacent regions of northern Kyrgyzstan, i.e., the valley of the Chu and Chon-Kemin rivers with the adjacent northern slopes of the Kyrgyz ridge and Kungey Alatau (Tolenova et al. 2021).

**Figure 40.**
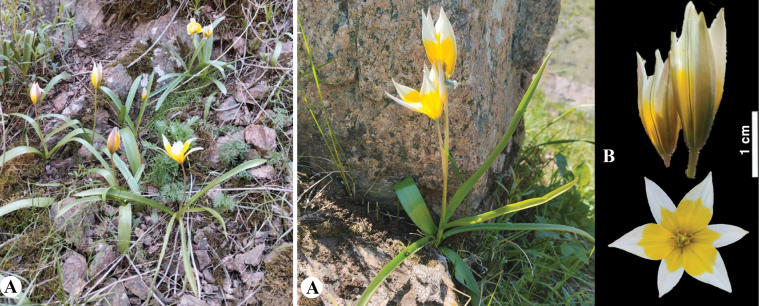
*Tulipatarda* in Kazakhstan **A** general habits **B** flowers (Photos: **A, B** by A. Tolenova).

#### ﻿ Tulipa tetraphylla

Taxon classificationPlantaeLilialesLiliaceae

Regel, Trudy Imp. S. Peterburgsk. Bot. Sada 3: 296 (1875).

55257C91-6039-5BEE-BF51-FFA9577B5118

Fig. 41

##### Type.

Kyrgyzstan • Turkestaniae in valle Kotschkura, Kaulbars, *Baro* (holotype LE, not located).

##### General distribution.

Kazakhstan, Kyrgyzstan, and China (Xinjiang) (POWO 2024).

##### Distribution in Kazakhstan and habitat.

Ketmen Terskey Alatau, and Trans-Ili Kungey Alatau. This species grows on stony slopes, and steppe areas in the lower and middle zones of the mountains.

##### Conservation status.

*Tulipatetraphylla* is a least concern species at the global level (IUCN 2024).

##### Phenology.

Flowering in April–May; fruiting in June –July.

##### Note.

*Tulipatetraphylla* was described by E.L. Regel in 1875 from collections from Central Tian Shan (Kochkur River basin, Kyrgyzstan). Spontaneous hybrids with *T.kolpakowskiana* have also been reported (Ivashchenko and Belyalov 2019; Vvedensky 1935). *Tulipatetraphylla* is often found in its autotetraploid form (Wilson 2023), although it also has a diploid form (Botschantzeva 1962). The new species *T.toktogulica* B.D.Wilson & Lazkov from Jalal-Abad province was described in 2022. It is morphologically similar to *T.tetraphylla* but differs by fewer leaves (3 leaves) and weakly fragrant flowers.

**Figure 41.**
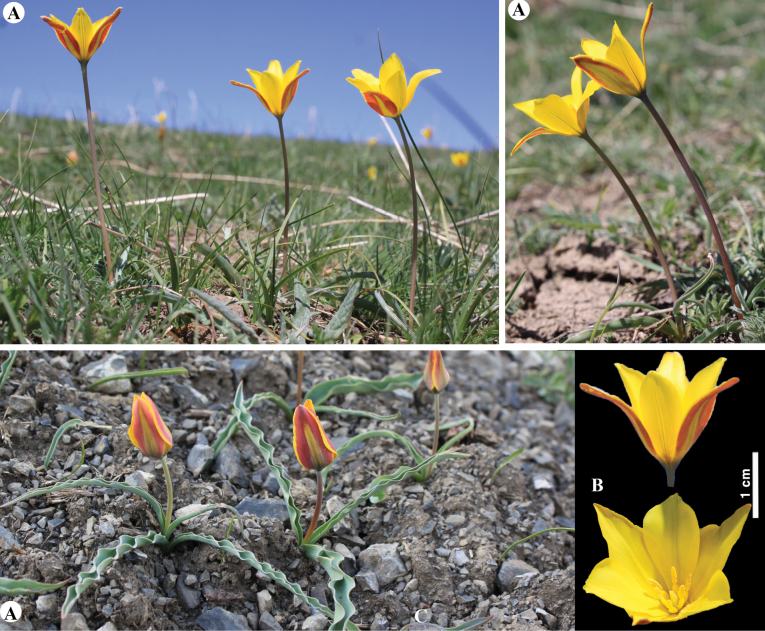
*Tulipatetraphylla* in Kazakhstan **A** general habits **B** flowers. (Photos: **A, B** by S. Mukhtubayeva).

#### ﻿ Tulipa turgaica

Taxon classificationPlantaeLilialesLiliaceae

Perezhogin, Novosti Sist. Vyssh. Rast. 45: 145 (2014).

27A17D87-B091-5FCE-9524-02FEE4428E31

Fig. 42

##### Type.

Kazakhstan • Prov. Kostanay, Zhangeldin distr., pag. Turgay, 2 May 2009, *Yu. Perezhogin* (LE).

##### General distribution.

Endemic to Kazakhstan (Kubentayev et al. 2024).

##### Distribution in Kazakhstan and habitat.

Aktobe, and Turgay. This species grows on dry, deserted clay steppes.

##### Conservation status.

The IUCN conservation status of this species requires assessment.

##### Phenology.

Flowering in April–May; fruiting in June–July.

##### Notes.

*Tulipaturgaica* was first invalidly described in 2013 by Yu. V. Perezhogin from Northern Kazakhstan (Turgai) and validated in 2014. This species is morphologically similar to *T.biebersteiniana* but differs by several flowers and grows in drier habitats (Perezhogin 2013). Some taxonomists classified *T.biebersteiniana* as a synonym of the widespread European T.sylvestrissubsp.australis (Christenhusz et al. 2013; Everett et al. 2013). When we studied populations of *T.turgaica* from the type locality, a high frequency of plants with several flowers was not observed. Individuals with 2–3 flowers were found in the population. However, *T.turgaica* differs morphologically from mesophytic *T.biebersteiniana* in that it does not exhibit vegetative propagation and does not form lateral stolons like *T.biebersteiniana*. Further studies are required to investigate the taxonomy of these species.

**Figure 42.**
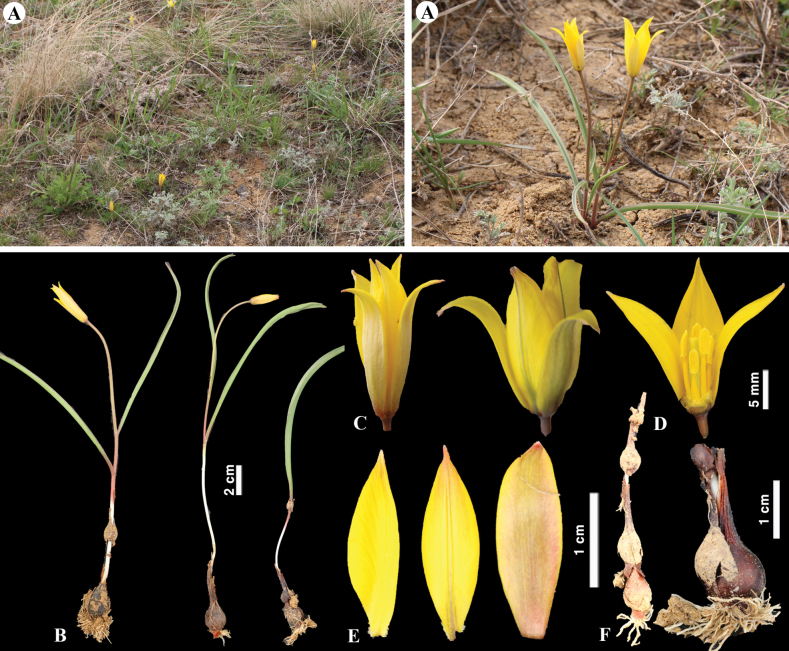
*Tulipaturgaica* in Kazakhstan **A, B** general habits **C, D** flowers **E** tepals **F** bulbs. (Photos: **A–F** by S. Kubentayev).

#### ﻿ Tulipa turkestanica

Taxon classificationPlantaeLilialesLiliaceae

(Regel) Regel, Trudy Imp. S. Peterburgsk. Bot. Sada 3: 296 (1875).

91F8CC72-D3B2-509B-A588-7124EA6AAE59

Fig. 43

##### Type.

Uzbekistan • ‘Chiwa’, Korolkow & Krause (COI-00050870) [lectotype designated by Christenhusz et al. 2013: 325].

##### General distribution.

Kazakhstan, Kyrgyzstan, Tajikistan, Uzbekistan, and China (Xinjiang) (POWO 2024).

##### Distribution in Kazakhstan and habitat.

Western Tian Shan, Karatau. This species grows in clayey and gravelly slopes from the foothills to the alpine zone (800–3000 m a.s.l.).

##### Conservation status.

*Tulipaturkestanica* is a least concern species at the global level (IUCN 2024).

##### Phenology.

Flowering in March–June; fruiting June –July.

##### Notes.

Eduard Ludvigovich Regel originally described *T.turkestanica* as Tulipasylvestrisvar.turkestanica Regel in 1873, but later reclassified it as an independent species in 1875 (Ivashchenko and Belyalov 2019). Morphologically, this species is similar to *T.bifloriformis*, which differs by its erect buds and woolly pubescence at the top of the bulb scales (Vvedensky 1935). Transitional forms close to *T.bifloriformis* and *T.orthopoda* occur in nature (Ivashchenko and Belyalov 2019), making identifying these taxa difficult. These forms have been poorly studied and require additional research. The range of *T.turkestanica* might be restricted to Pamir-Alai, and this species might have been replaced by *T.bifloriformis* in Kazakhstan. However, this hypothesis requires further confirmation.

**Figure 43.**
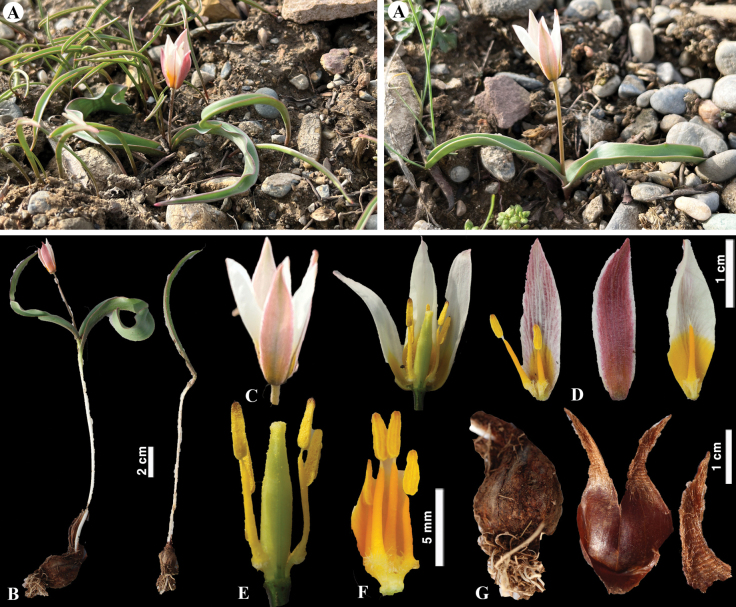
*Tulipaturkestanica* in Kazakhstan **A, B** general habits **C** flowers **D** tepals and stamens **E** gynoecium and stamens **F** stamens **G** bulb and bulb sheath. (Photos: **A–G** by S. Kubentayev).

#### ﻿ Tulipa × tschimganica

Taxon classificationPlantaeLilialesLiliaceae

Botschantz., Bot. Mater. Gerb. Inst. Bot. Akad. Nauk Uzbeksk. SSR. 16: 10 (1961).

87223A1F-4D17-5DA4-A03A-933766F3B45E

Fig. 44

##### Type.

Uzbekistan • Grown in the Botanical Garden of the Academy of Sciences of Uzbek SSR from bulbs collected by Z.P. Botschantzeva in 1959 in the gorge in piedmonts of Greater Chimgan, on fine earth among stones, Botschantzeva 99 (holotype TASH000526!).

##### General distribution.

Kazakhstan, Kirgizstan, and Uzbekistan.

##### Distribution in Kazakhstan and habitat.

Western Tian Shan. This species grows on rubbly and stony slopes in lower and middle mountain belts (1400–1700 m a.s.l.).

##### Conservation status.

Not assessed at the global level. However, this nothospecies is not protected in Kazakhstan.

##### Phenology.

Flowering in April–May; fruiting June–July.

##### Notes.

Tulipa×tschimganica was described in 1961 by Z.P. Botschantzeva from specimens grown in the Tashkent Botanical Garden from bulbs collected in Bolshoi Chimgan Gorge (Uzbekistan) (Ivashchenko and Belyalov 2019). In Kazakhstan, the species was first discovered in 2003 in the territory of the Keles Forestry, Karabausai tract (Karzhantau Ridge) along the rubbly hollows of the northwestern and north-eastern slopes at an altitude of 1600–1700 m a.s.l. (Ivashchenko et al. 2006). The origin of this species is relatively controversial; some consider it a separate species (Botschantzeva 1962; Van Raamsdonk et al. 1997;Pratov et al. 2006; Zonneveld 2009), whereas others consider it a hybrid of *T.greigii* and *T.kaufmanniana* (Vvedensky and Kovalevskaya 1971) or a hybrid of *T.dubia* and *T.kaufmanniana* (Christenhusz et al. 2013). Tojibaev (2010) and Tojibaev and Beshko (2014) previously considered this species a subspecies of T.kaufmannianasubsp.tschimganica but it was listed as a separate species later in the synopsis of the genus *Tulipa* (Liliaceae) in Uzbekistan (Tojibaev et al. 2022).

**Figure 44.**
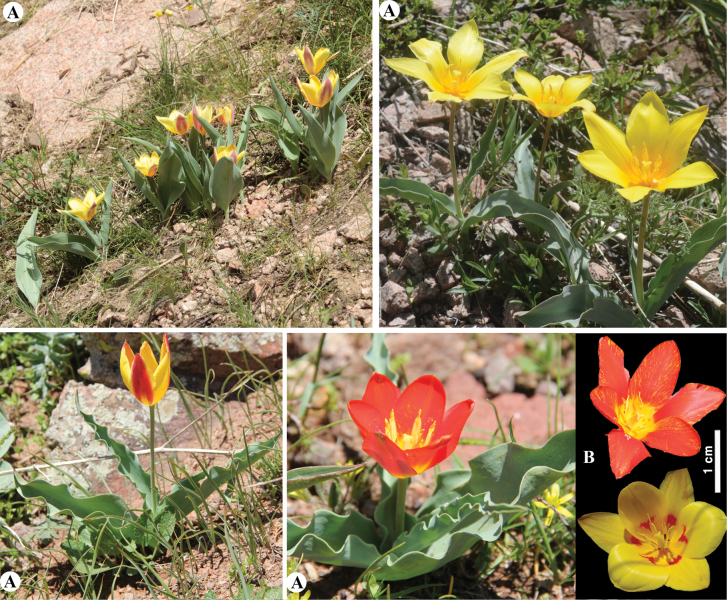
*Tulipa×tschimganica* in Uzbekistan **A** general habits **B** flowers. (Photos: **A, B** by K. Tojibaev).

#### ﻿ Tulipa uniflora

Taxon classificationPlantaeLilialesLiliaceae

(L.) Besser ex Baker, J. Linn. Soc., Bot. 14: 295 (1874).

A3CFC93A-554E-524D-BF15-A6C04117AF4F

Fig. 45

##### Type.

Russia • “In Siberiae montis Sini Sopka”, *E. Laxmann* (LE; isoneotype K-000844631) [neotype designated by Levichev, 1997].

##### General distribution.

China (Inner Mongolia, Xinjiang), Kazakhstan, Russia (Altay, Chita, Irkutsk, Krasnoyarsk, Tuva), and Mongolia (Baasanmunkh et al. 2022).

##### Distribution in Kazakhstan and habitat.

Altai, Tarbagatay, and Zaysan. This species grows in sandy places, gravelly slopes, and as shrubs from desert foothills to the upper belt of mountains.

##### Conservation status.

*Tulipauniflora* is a near threatened species at the global level (IUCN 2024); it is included in the Red Book of Kazakhstan (Category III).

##### Phenology.

Flowering in April–May; fruiting June –July.

##### Notes.

This species was first described by Linnaeus in 1767 as *Ornithogalumuniflorum* L. from specimens collected from the Altai Mountains. Don (1836) described the new genus *Orithyia* D.Don. from specimens of *Ornithogalumuniflorum*. It was not until 1874 that Ch. Bassey assigned this species to *Tulipa*. *Tulipauniflora* is similar to *T.heteropetala*; their differences are outlined in the description of *T.heteropetala* above.

**Figure 45.**
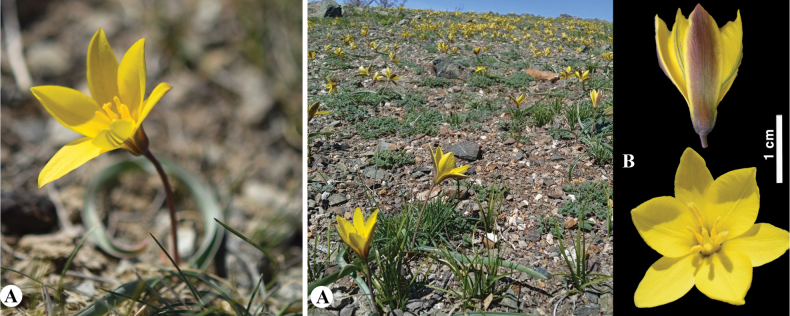
*Tulipauniflora* in Kazakhstan **A** general habits **B** flowers. (Photos: **A, B** by Sh. Baasanmunkh).

#### ﻿ Tulipa zenaidae

Taxon classificationPlantaeLilialesLiliaceae

Vved., Byull. Sredne-Aziatsk. Gosud. Univ. 21: 150 (1935).

FD2F8013-4A6D-52C9-A992-23BE44B9B436

Fig. 46

##### Type.

Kyrgyzstan • ‘Habitat in montibus Alexandricis (Tian-Shan)’, 14 June 1932, *Vvedensky 280* (TASH).

##### General distribution.

Kazakhstan, and Kyrgyzstan (Sennikov and Tojibaev 2021).

##### Distribution in Kazakhstan and habitat.

Kyrgyz Alatau. This species grows in fine earth and gravelly slopes, usually with thickets of bushes, in the lower mountain belt.

**Figure 46.**
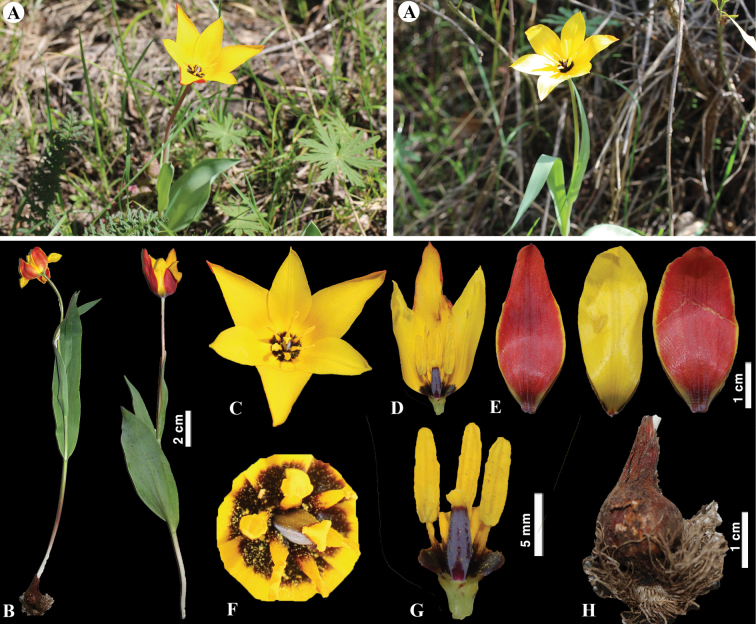
*Tulipazenaidae* in Kazakhstan **A, B** general habits **C, D** flowers **E** tepals **F, G** gynoecium and stamens **H** bulb (Photos: **A–H** by S. Kubentayev).

##### Conservation status.

*Tulipazenaidae* is a vulnerable species at the global level (IUCN 2024); it is included in the Red Book of Kazakhstan (Category II).

##### Phenology.

Flowering in April–May; fruiting May–July.

##### Notes.

*Tulipazenaidae* was described by A.I. Vvedensky in 1935 from Kyrgyz Ridge (Mount Shekule). The species was named in honor of Zinaida Botschantzeva, who devoted her life to studying Central Asian tulips (Ivashchenko and Belyalov 2019). According to the latest classification, this species was assigned the synonym of *T.lehmanniana* (Christenhusz et al. 2013; Everett et al. 2013). Wilson (2023) later confirmed that *T.zenaidae* is a different species from *T.lehmanniana* which we agree with in this study.

## ﻿Conclusion

**Figure 47.**
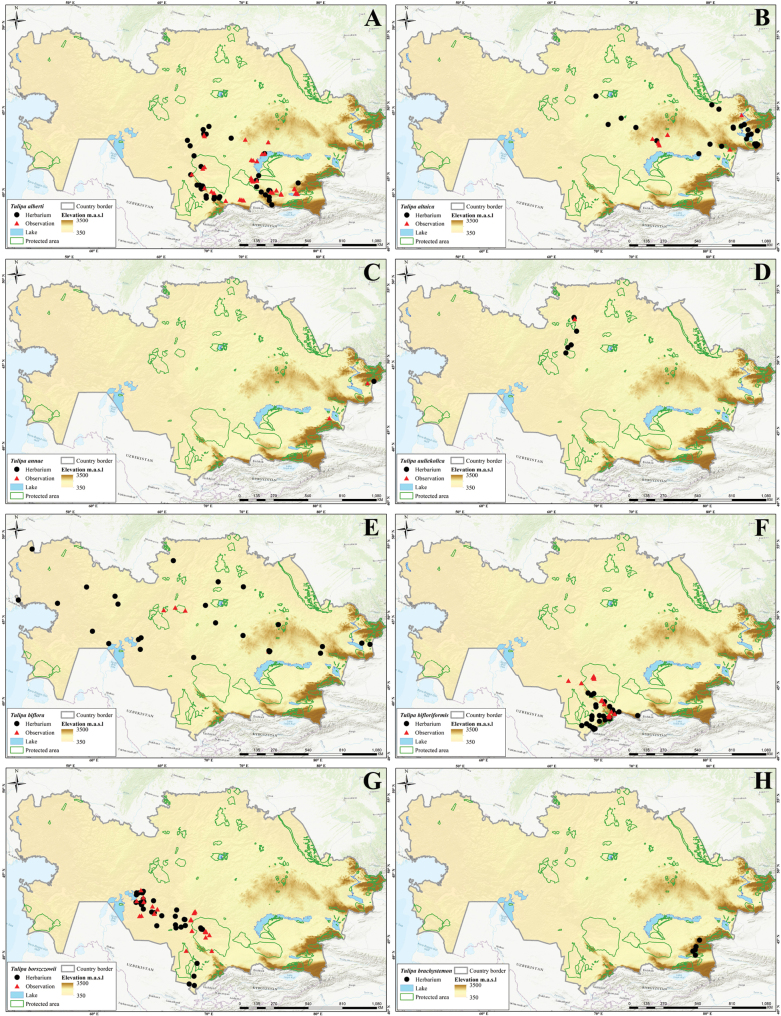
Distribution maps of *Tulipa* in Kazakhstan **A***T.alberti***B***T.altaica***C***T.annae***D***T.auliekolica***E***T.biflora***F***T.bifloriformis***G***T.borszczowii***H***T.brachystemon*.

**Figure 48.**
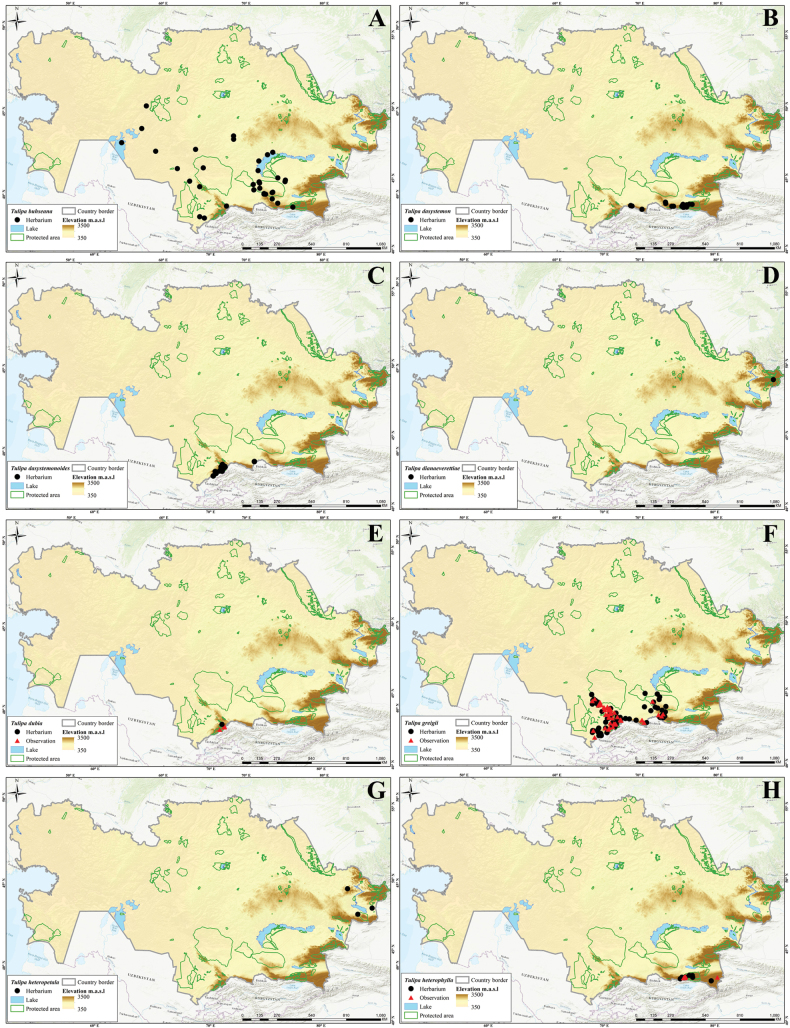
Distribution maps of *Tulipa* in Kazakhstan **A***T.buhseana***B***T.dasystemon***C***T.dasystemonoides***D***T.dianae-verettiae***E***T.dubia***F***T.greigii***G***T.heteropetala***H***T.heterophylla*.

**Figure 49.**
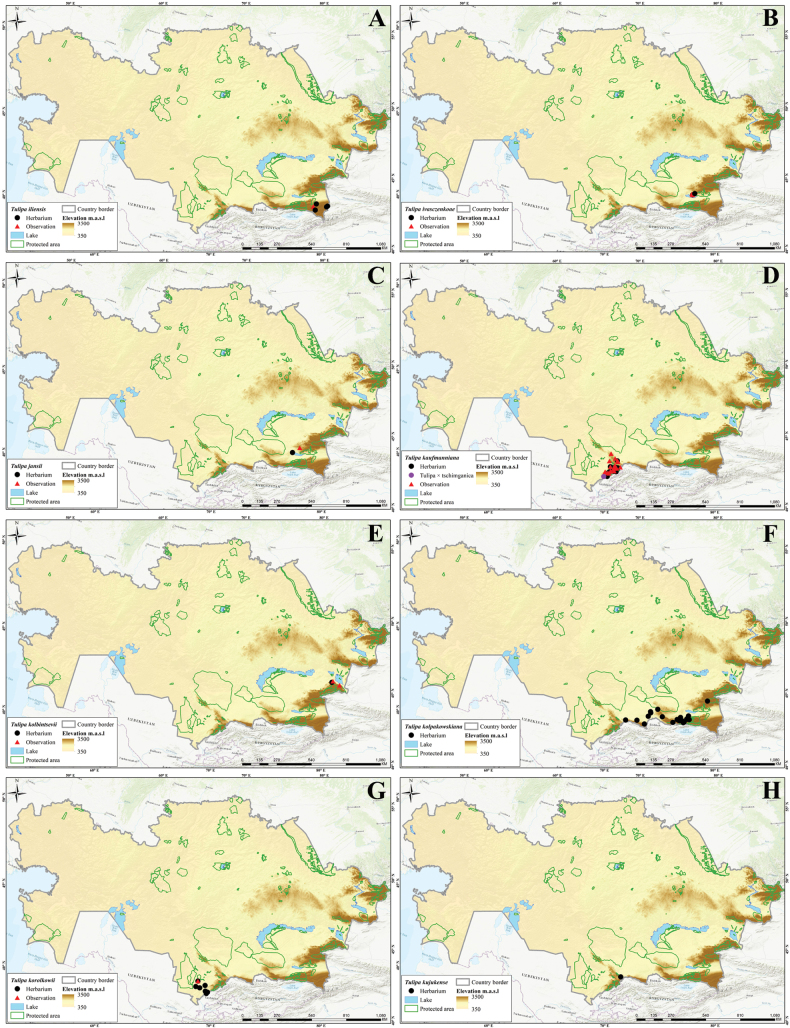
Distribution maps of *Tulipa* in Kazakhstan **A***T.iliensis***B***T.ivasczenkoae***C***T.jansii***D***T.kaufmanniana***E***T.kolbintsevii***F***T.kolpakowskiana***G***T.korolkowii***H***T.kujukense*.

**Figure 50.**
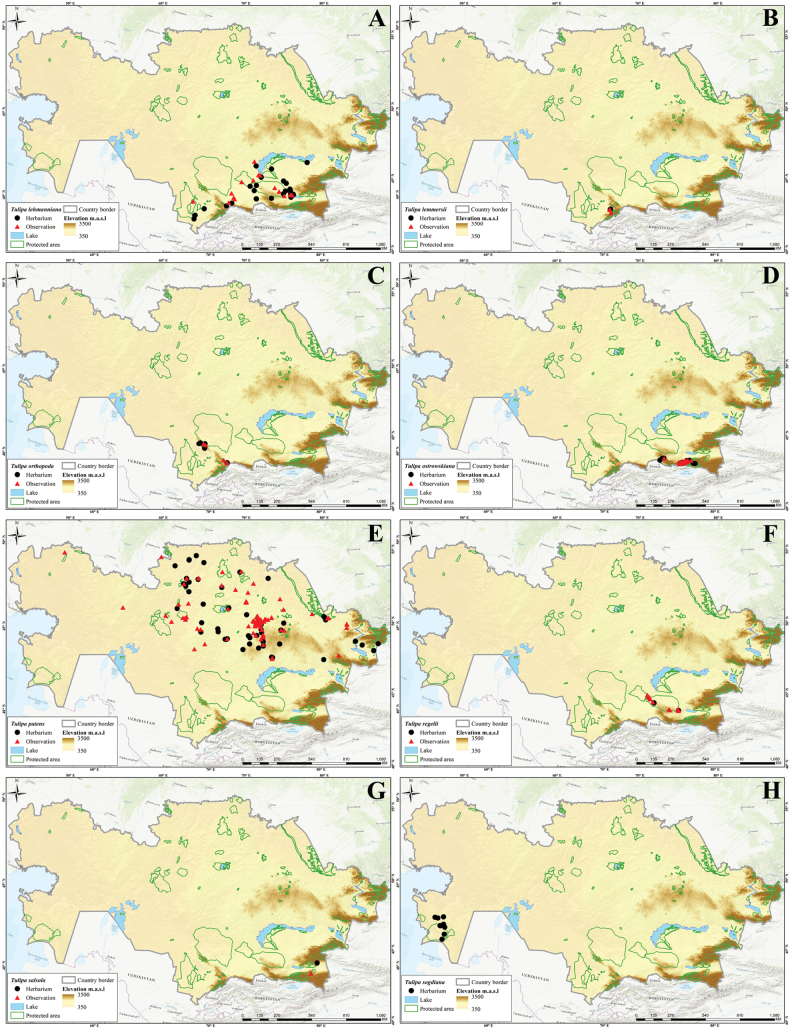
Distribution maps of *Tulipa* in Kazakhstan **A***T.lehmanniana***B***T.lemmersii***C***T.orthopoda***D***T.ostrowskiana***E***T.patens***F***T.regelii***G***T.salsola***H***T.sogdiana*.

**Figure 51.**
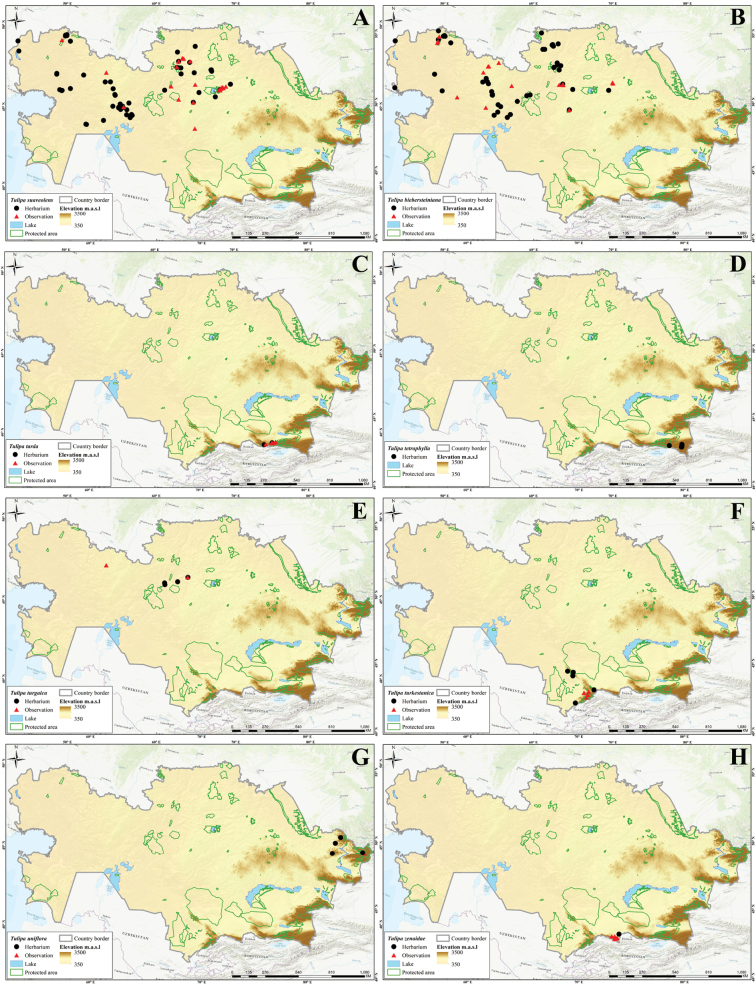
Distribution maps of *Tulipa* in Kazakhstan **A***T.suaveolens***B***T.biebersteiniana***C***T.tarda***D***T.tetraphylla***E***T.turgaica***F***T.turkestanica***G***T.uniflora***H***T.zenaidae*.

The total number of *Tulipa* species varies according to the source, representing 90–120 species worldwide. Similarly, the number of tulips in Kazakhstan is 32–42, according to various sources. Therefore, we revisited the species diversity of *Tulipa* in Kazakhstan based on field observations, extensive herbarium specimens, and literature data. We confirmed that 41 tulip taxa are currently distributed in Kazakhstan, of which 13 species are endemic. The present study provides valuable information on the tulip species richness in Kazakhstan, focusing on taxonomic keys, historical notes, species diversity, point distribution maps (Figs 47–51), phylogenetic analysis, and photographs of the wild plants. Notably, we identified several high-biodiversity hotspots, particularly in the floristic regions of Western Tian Shan and Trans-Ili Kungey Alatau. We compared herbarium data and iNaturalist observations of tulips, revealing interesting trends in collection periods and observation frequencies. Although herbarium specimens were predominantly collected before 2000, the number of iNaturalist observations has since steadily increased demonstrating the growing contribution of citizen scientists to biodiversity research.

## Supplementary Material

XML Treatment for Sect. Tulipa

XML Treatment for Sect. Lanatae

XML Treatment for Sect. Kolpakowskianae

XML Treatment for Sect. Vinistriatae

XML Treatment for Sect. Spiranthera

XML Treatment for Subgenus Orithyia

XML Treatment for Sect. Orithyia

XML Treatment for Sect. Sylvestres

XML Treatment for Sect. Biflores

XML Treatment for Tulipa alberti

XML Treatment for Tulipa altaica

XML Treatment for Tulipa annae

XML Treatment for Tulipa auliekolica

XML Treatment for Tulipa biebersteiniana

XML Treatment for Tulipa biflora

XML Treatment for Tulipa bifloriformis

XML Treatment for Tulipa borszczowii

XML Treatment for Tulipa brachystemon

XML Treatment for Tulipa buhseana

XML Treatment for Tulipa dasystemon

XML Treatment for Tulipa dasystemonoides

XML Treatment for Tulipa dianaeverettiae

XML Treatment for Tulipa dubia

XML Treatment for Tulipa greigii

XML Treatment for Tulipa heteropetala

XML Treatment for Tulipa heterophylla

XML Treatment for Tulipa iliensis

XML Treatment for Tulipa ivasczenkoae

XML Treatment for Tulipa jansii

XML Treatment for Tulipa kaufmanniana

XML Treatment for Tulipa kolbintsevii

XML Treatment for Tulipa kolpakowskiana

XML Treatment for Tulipa korolkowii

XML Treatment for Tulipa kujukense

XML Treatment for Tulipa lehmanniana

XML Treatment for Tulipa lemmersii

XML Treatment for Tulipa orthopoda

XML Treatment for Tulipa ostrowskiana

XML Treatment for Tulipa patens

XML Treatment for Tulipa regelii

XML Treatment for Tulipa salsola

XML Treatment for Tulipa sogdiana

XML Treatment for Tulipa suaveolens

XML Treatment for Tulipa tarda

XML Treatment for Tulipa tetraphylla

XML Treatment for Tulipa turgaica

XML Treatment for Tulipa turkestanica

XML Treatment for Tulipa × tschimganica

XML Treatment for Tulipa uniflora

XML Treatment for Tulipa zenaidae

## ﻿Additional information

### Author ORCIDs

Serik A. Kubentayev https://orcid.org/0000-0002-0369-0591

Shukherdorj Baasanmunkh https://orcid.org/0000-0003-4224-9376

Daniyar T. Alibekov https://orcid.org/0000-0003-1555-1430

Komiljon Sh. Tojibaev https://orcid.org/0000-0003-2846-5777

Nudkhuu Nyamgerel https://orcid.org/0000-0002-9110-423X

Anna A. Ivashchenko https://orcid.org/0000-0002-8526-4296

Zagarjav Tsegmed https://orcid.org/0000-0002-6024-4918

Vladimir G. Epiktetov https://orcid.org/0000-0001-5406-0568

Gulnara T. Sitpayeva https://orcid.org/0000-0003-4614-6155

Klara S. Izbastina https://orcid.org/0000-0002-6418-1950

Zhansaya T. Idrissova https://orcid.org/0000-0003-3231-8518

Saule K. Mukhtubayeva https://orcid.org/0000-0001-5921-3113

Nurganym B. Abubakirova https://orcid.org/0000-0002-7503-0955

Hee-Young Gil https://orcid.org/0000-0003-3714-0827

Hyeok Jae Choi https://orcid.org/0000-0003-3644-6795

### Data availability

All of the data that support the findings of this study are available in the main text or Supplementary Information.

## Supplementary materials

Supplementary material 1 Supplementary Information Data type doc Explanation note Sample list for phylogenetic analysis, summary of distribution and conservation status, and list of specimens examined. https://binary.pensoft.net/file/1206007This dataset is made available under the Open Database License (http://opendatacommons.org/licenses/odbl/1.0/). The Open Database License (ODbL) is a license agreement intended to allow users to freely share, modify, and use this Dataset while maintaining this same freedom for others, provided that the original source and author(s) are credited.Serik A. Kubentayev, Shukherdorj Baasanmunkh, Daniyar T. Alibekov, Komiljon Sh. Tojibaev, Nudkhuu Nyamgerel, Anna A. Ivashchenko, Zagarjav Tsegmed, Vladimir G. Epiktetov, Gulnara T. Sitpayeva, Klara S. Izbastina, Zhansaya T. Idrissova, Saule K. Mukhtubayeva, Nurganym B. Abubakirova, Hee-Young Gil, Hyeok Jae Choi

## References

[B1] Abdulina SA (1999) List of Vascular Plants of Kazakhstan. Almaty, 187 pp. [In Russian]

[B2] Abduraimov OS Shomurodov HF Daniyarov SA Mamatkasimov OT Teshaev MI (2020) Distribution and Current State of Rare and Endangered *Tulips* (Liliaceae) Arid Zones of Uzbekistan. American Journal of Plant Sciences 11(5): 736–744. 10.4236/ajps.2020.115053

[B3] Almerekova S Yermagambetova M Ivashchenko A Abugalieva S Turuspekov Y (2024a) Assessment of complete plastid genome sequences of *Tulipaalberti* Regel and *Tulipagreigii* Regel species from Kazakhstan. Genes 15(11): 1447. 10.3390/genes15111447 39596647 PMC11593697

[B4] Almerekova S Yermagambetova M Ivaschenko A Turuspekov Y Abugalieva S (2024b) Comparative analysis of plastome sequences of seven *Tulipa* L. (Liliaceae Juss.) species from section Kolpakowskianae Raamsd. Ex Zonn and Veldk. International Journal of Molecular Sciences 25(14): 7874. 10.3390/ijms25147874 39063115 PMC11277319

[B5] Asatulloev T Dekhkonov D Yusupov Z Tojiboeva U Cai L Tojibaev K Sun W (2023) Ecoregional and phytogeographical insights into the distribution of *Tulipa* in the ‘Nature Imperiled’ Area of Central Asia for effective conservation. Diversity 15(1195): 1–17. 10.3390/d15121195

[B6] Asgari D Babaei A Naghavi MR Kiani M (2020) Biodiversity status of *Tulipa* (Liliaceae) in Iran inferred from molecular characterization. Horticulture, Environment and Biotechnology 61(3): 559–567. 10.1007/s13580-019-00158-0

[B7] Baasanmunkh S Urgamal M Oyuntsetseg B Sukhorukov AP Tsegmed Z Son DC Erst A Oyundelger K Kechaykin AA Norris J Kosachev P Ma J-S Chang KS Choi HJ (2022) Flora of Mongolia: Annotated checklist of native vascular plants. PhytoKeys 192: 63–169. 10.3897/phytokeys.192.79702 35437387 PMC8938380

[B8] Baitenov MS (2001) Flora of Kazakhstan. Generic complex of flora. Science Publishers, Almaty, 280 pp. [In Russian]

[B9] Baitulin IO [Ed.] (2014) The Red Book of Kazakhstan (plants), Vol. 2(1). Art Print XXI, Astana, 452 pp. [In Russian]

[B10] Botschantzeva ZP (1962) Tulips: morphology, cytology and biology. [In Russian] [English translation: Varekamp HQ (1982) CRC Press, Rotterdam, Balkema, The Netherlands, 230 pp.]

[B11] Cherepanov SK (1995) Vascular plants of Russia and adjacent states (the Former USSR). Cambridge University Press, New York.

[B12] Chernysheva OA Bukin YS Kulakova NV Mitrenina EY Murashko VV Khadeeva ER Erst AS Krivenko DA (2023) How many species of tulips of the subgenus Orithyia (*Tulipa*, Liliaceae) are in Southern Siberia? Botanica Pacifica 12(1): 29–37. 10.17581/bp.2023.12104

[B13] Christenhusz MJ Wilson B (2022) Proposal to reject the name *Tulipaurumiensis* (Liliaceae). Taxon 71(4): 906–907. 10.1002/tax.12786

[B14] Christenhusz MJ Govaerts R David JC Hall T Borland K Roberts PS Fay MF (2013) Tiptoe through the tulips-cultural history, molecular phylogenetics and classification of *Tulipa* (Liliaceae). Botanical Journal of the Linnean Society 172(3): 280–328. 10.1111/boj.12061

[B15] de Groot JJ Zonneveld BJM (2020) Two new tulip species from the Altai mountains, Kazakhstan. International Rock Gardener 122: 3–16.

[B16] de Groot JJ Zonneveld BJM (2024) Three new species of *Tulipa* from Central Asia. International Rock Gardener 168: 3–18.

[B17] Dekhkonov D Tojibaev K Makhmudjanov D Baasanmunkh S Yusupov Z Choi HJ Jang CG (2021) Mapping and analyzing the distribution of the species in the genus *Tulipa* (Liliaceae) in the Ferghana Valley of Central Asia. Korean Journal of Plant Taxonomy 51(3): 181–191. 10.11110/kjpt.2021.51.3.181

[B18] Dekhkonov D Tojibaev K Yusupov Z Makhmudjanov D Asatulloev T (2022) Morphology of tulips (*Tulipa*, Liliaceae) in its primary centre of diversity. Plant Diversity of Central Asia 1(1): 52–70. 10.54981/PDCA/vol1_iss1/a3

[B19] Don D [Ed.] (1836) *Orithyiauniflora*. The British Flower Garden, ser. 2, 4. Ridgway, London, 336 pp.

[B20] Eker I Babac MT Koyuncu M (2014) Revision of the genus *Tulipa* L. (Liliaceae) in Turkey. Phytotaxa 157(1): 1–112. 10.11646/phytotaxa.157.1.1

[B21] Eker İ Tarıkahya Hacıoğlu B Özgişi K (2024) Phylogeny and infrageneric classification of *tulips*. Plant Systematic and Evolution 310: 23. 10.1007/s00606-024-01907-0

[B22] Epiktetov VG Belyalov OV (2013) A new species of the genus *Tulipa* L. (Liliaceae) from Kazakhstan. Turczaninowia 16(3): 5–7. 10.14258/turczaninowia.16.3.1

[B23] Esri (2012) Advancing the power of geography. For business, government, and society. https://www.esri.com/enus/about/about‐esri/overview

[B24] Everett D Fay MF Christenhusz MJ Wilford R (2013) The genus *Tulipa*. Tulips of the world. Kew Garden Press, London, 379 pp.

[B25] Hajdari A Pulaj B Schmiderer C Malaj X Wilson B Lluga-Rizani K Mustafa B (2021) A phylogenetic analysis of the wild *Tulipa* species (Liliaceae) of Kosovo based on plastid and nuclear DNA sequence. Advanced Genetics 2(1): e202100016. 10.1002/ggn2.202100016 PMC9744470 36620432

[B26] Hall AD (1940) The genus *Tulipa*. Royal horticultural society, London, 171 pp.

[B27] Hoog MH (1973) On the origin of *Tulipa*. Lilies and other Liliaceae. Royal Horticulture Society, London, England, 47–64.

[B28] IUCN (2024) The IUCN Red List of Threatened Species. https://www.iucnredlist.org [accessed 18 June 2024]

[B29] Ivashchenko AA (1987) Ephemeroids of Aksu-Dzhabagly Reserve. Nauka, Alma-Ata, 172 pp. [In Russian]

[B30] Ivashchenko AA (2005) *Tulips* and other bulbous plants of Kazakhstan. Two Capitals, Almaty, 192 pp.

[B31] Ivashchenko AA (2007) Treasures of the flora of Kazakhstan. On pages of the Red Book. Almatykitap, Almaty, 125 pp. [In Russian]

[B32] Ivashchenko AA Belyalov OV (2019) Kazakhstan is the birthplace of *Tulips*. Almaty: Atamura, 368 pp. [In Russian]

[B33] Ivashchenko AA Olontseva AH Nelina NV (2006) About some rare and new plants of the Western Tian Shan for Kazakhstan / Mat. Conf. Current problems of ecology and nature management in Kazakhstan and adjacent territories. Pavlodar, 218–220.

[B34] Kamelin RV (1990) Flora of the Syrdarya Karatau. Science Publishers, Leningrad, 184 pp. [In Russian]

[B35] Kolbintsev V (2016) Image of *Tulipaannae* J. de Groot & Zonn. Plantarium. Plants and lichens of Russia and neighboring countries: open online galleries and plant identification guide. https://www.plantarium.ru/lang/en/page/image/id/416158.html [accessed on 5 Dec 2024]

[B36] Kubentayev SA Alibekov DT Perezhogin YV Lazkov GA Kupriyanov AN Ebel AL Izbastina KS Borodulina OV Kubentayeva BB (2024) Revised checklist of endemic vascular plants of Kazakhstan. PhytoKeys 238: 241–279. 10.3897/phytokeys.238.114475 38456166 PMC10918586

[B37] Kupriyanov AN (2020) Synopsis of the Flora of the Kazakh Uplands. Geo, Novosibirsk, 423 pp. [In Russian]

[B38] Kutlunina NA Polezhaeva MA Permyakova MV (2013) Morphological and AFLP analysis of relationships between tulip species *Tulipabiebersteiniana* (Liliaceae). Russian Journal of Genetics 49(4): 401–410. 10.1134/S1022795413040091 23866623

[B39] Laffan SW Crisp MD (2003) Assessing endemism at multiple spatial scales, with an example from the Australian vascular flora. Journal of Biogeography 30(4): 511–520. 10.1046/j.1365-2699.2003.00875.x

[B40] Laffan SW Lubarsky E Rosauer DF (2010) Biodiverse, a tool for the spatial analysis of biological and related diversity. Ecography 33(4): 643–647. 10.1111/j.1600-0587.2010.06237.x

[B41] Levichev IG (1997) The review of the genus *Gagea* (Liliaceae) in the flora of the Far East. Botanicheskii Zhurnal 82: 77–92. [In Russian]

[B42] Li J Price M Su DM Zhang Z Yu Y Xie DF Zhou SD He XJ Gao XF (2021) Phylogeny and comparative analysis for the plastid genomes of five *Tulipa* (Liliaceae). BioMed Research International 2021(3): 1–10. 10.1155/2021/6648429 34239930 PMC8235973

[B43] Mordak EV (1990) Quid est *Tulipaschrenkii* Regel et *T.heteropeta* Ledeb. (Liliaceae)? Novosti Systematiki Vysshikh Rastenii 27: 27–32. [In Russian].

[B44] Mordak EV (1992) *Tulipauniflora* (L.) Besser ex Baker. N° 7197–7197a. In: Schedae ad herbarium florae Rossicae et civitatum collimitanearum ab Instituto Botanico Academie Scientiarum Rossicae editum, BIN RAN, St. Petersburg. [In Russian]

[B45] Pavlov NV [Ed.] (1956) Flora of Kazakhstan, Vol. 1. Academy of Sciences of the Kazakh SSR, Alma-Ata, 354 pp. [In Russian]

[B46] Pavord A (1999) The Tulip: the Story of a Flower That Has Made Men Mad. St. Martin’s Press, London, 439 pp.

[B47] Perezhogin YuV (2013) New species of tulips from Northern Kazakhstan. Botanicheskii Zhurnal 98: 1558–1563. [In Russian]

[B48] Perezhogin YuV Kulikov PV Kurlov SI (2015) Addition to the flora of Kazakhstan. Botanicheskii Zhurnal 100(5): 501–503. [In Russian]

[B49] Polyakov PP [Eds] (1958) Flora of Kazakhstan, Vol. 2. Academy of Sciences of the Kazakh SSR, Alma-Ata, 353 pp. [In Russian]

[B50] POWO (2024) Plants of the World Online. Facilitated by the Royal Botanic Gardens, Kew. http://www.plantsoftheworldonline.org/ [accessed 01 January 2024]

[B51] Pratov UP Sharipov AK Ashurmetov OA Tojibaev KS (2006) Tulips of the Western Tien Shan. Chinor ENK Press, Tashkent, 126 pp.

[B52] Qin D Liu W Tian J Ju X (2024) Potentially suitable area and change trends of *Tulipailiensis* under climate change. Phyton 93(5): 981–1005. 10.32604/phyton.2024.049668

[B53] Rambaut A (2012) FigTree v1. 4. Molecular evolution, phylogenetics and epidemiology. Edinburgh Univ Edinburgh, IInstitute of Evolutionary Biology.

[B54] Regel EA (1879) *Tulipadasystemon*. Trudy Imperatorskago Sainkt Petersburgkago Botaniskogo Sada 6: 507.

[B55] Rukšāns J (2019) Some geophytes from Berkara Gorge in Karatau Mountain Ridge (Kazakhstan) and *Tulipaberkariensis* Rukšāns species nova – an “old” new species of *Tulipa* (Liliaceae). International Rock Gardener 109: 26–39.

[B56] Rukšāns J Zubov D (2022) Two new Tulipa species from sect. Biflores (subgen. Eriostemones, Liliaceae) described from Iran, Zagros Mountains and Kazakhstan, Zhetysu. International Rock Gardener 148: 2–51.

[B57] Samartza I Tsaball A Sakellariou M Mahnev P Tsiripidis I Krigas N Tsoktouridis G (2024) Taxonomic and molecular characterization of 15 wild-growing tulip species of Greece using the internal transcribed spacer (ITS) nuclear marker in combination with the psbA-trnH and trnL/trnF plastid markers. Biotechnology, Biotechnological Equipment 38(1): 2337694. 10.1080/13102818.2024.2337694

[B58] Sennikov AN Tojibaev KSh [Eds] (2021) Checklist of vascular plants of the Tian-Shan Mountain System. Korea National Arboretum, Pocheon, 607 pp. [In Russian]

[B59] Sievers F Barton GJ Higgins DG (2020) Multiple Sequence Alignment. Bioinformatics 227: 227–250.

[B60] Stamatakis A (2006) RAcML-VL-HPC: Maximum likelihood-based phylogenetic analysis with thousands of taxa and mixed models. Bioinformatics 22(21): 2688–2690. 10.1093/bioinformatics/btl446 16928733

[B61] Sutula M Kakanay A Tussipkan D Dzhumanov S Manabayeva S (2024) Phylogenetic analysis of rare and endangered *Tulipa* species (Liliaceae) of Kazakhstan based on universal barcoding markers. Biology 13(6): 365. 10.3390/biology13060365 38927245 PMC11200791

[B62] Thiers B (2023) Index Herbariorum: A Global Directory of Public Herbaria and Associated Staff. New York Botanical Garden’s Virtual Herbarium. http://sweetgum.nybg.org/science/ih/ [Accessed 25 March 2023]

[B63] Tojibaev KS (2010) What is *Tulipatschimganica Z.* Botsch. and *T.butkovii* Z. Botsch. Uzbek Biological Journal 3: 42–46.

[B64] Tojibaev KS Beshko N (2014) Reassessment of diversity and analysis of distribution in *Tulipa* (Liliaceae) in Uzbekistan. Nordic Journal of Botany 33(3): 324–334. 10.1111/njb.00616

[B65] Tojibaev K Dekhkonov D Ergashov I Sun H Deng T Yusupov Z (2022) The synopsis of the genus *Tulipa* (Liliaceae) in Uzbekistan. Phytotaxa 573(2): 163–214. 10.11646/phytotaxa.573.2.2

[B66] Tolenova AD Ivashchenko AA Moysiyenko II (2021) Plant communities with the participation of *Tulipatarda* Stapf. in Kazakhstan: Floristic composition and analysis. Eurasian Journal of Ecology 66: 61–74. 10.26577/EJE.2021.v66.i1.06

[B67] Tussipkan D Shevtsov V Ramazanova M Rakhimzhanova A Shevtsov A Manabayeva S (2024) Kazakhstan tulips: Comparative analysis of complete chloroplast genomes of four local and endangered species of the genus *Tulipa* L. Frontiers in Plant Science 15: 1433253. 10.3389/fpls.2024.1433253 PMC11588485 39600902

[B68] Van Raamsdonk LW De Vries T (1995) Species relationships and taxonomy in Tulipasubg.Tulipa (Liliaceae). Plant Systematics and Evolution 195(1–2): 13–44. 10.1007/BF00982313

[B69] Van Raamsdonk LW Eikelboom W De Vries T Straathof TP (1997) The systematics of the genus *Tulipa* L. Acta Horticulturae (430): 821–828. 10.17660/ActaHortic.1997.430.131 [ISHS]

[B70] Veldkamp JF Zonneveld BJ (2012) The infrageneric nomenclature of *Tulipa* L. Plant Systematics and Evolution 298(1): 87–92. 10.1007/s00606-011-0525-0

[B71] Vvedensky AI [Ed.] (1935) Liliaceae – genus *Tulipa* L. Flora of the USSR, Vol. 4. Izsatel’stvo Akademii Nauk SSSR, Leningrad, 320–364.

[B72] Vvedensky AI [Ed.] (1941) The genus *Tulipa* L. Flora Uzbekistanica, Vol. 1. The Publishing house of the Academy of Sciences UzSSR, Tashkent, 502–520. [In Russian]

[B73] Vvedensky AI [Ed.] (1963) *Tulipa* L. Flora of Tajikistan, Vol. 2. Izsatel’stvo Akademii Nauk SSSR, Moscow & Leningrad, 249–269. [In Russian]

[B74] Vvedensky AI Kovalevskaya SS [Ed.] (1971) *Tulipa* L. Conspectus Florae Asiae Mediae, Vol. 2. The Publishing house of the Academy of Sciences UzSSR, Tashkent, 94–109. [In Russian]

[B75] WCSP (2024) World Checklist of Selected Plant Families. Facilitated by the Royal Botanic Gardens, Kew. http://wcsp.science.kew.org/ [Accessed 01 January 2024]

[B76] Wilson B (2023) *Tulipa*: the taxonomy and evolutionary history of the genus and its impact on conservation priorities in Central Asia. PhD Thesis, University of Cambridge. 10.17863/CAM.94432

[B77] Wilson B Dolotbakov A Burgess BJ Clubbe C Lazkov G Shalpykov K Ganybaeva M Sultangaziev O Brockington SF (2021) Central Asian wild tulip conservation requires a regional approach, especially in the face of climate change. Biodiversity and Conservation 30(6): 1705–1730. 10.1007/s10531-021-02165-z

[B78] Xing G Zhang H Zhang Y Lu J Wu T Tian Z Qu L (2023) The complete chloroplast genome of *Tulipasinkiangensis* Z.M. Mao (Liliaceae) with multi-flower. Mitochondrial DNA. Part B, Resources 8(1): 45–47. 10.1080/23802359.2022.2160217 PMC9828695 36632081

[B79] Yermagambetova M Almerekova S Ivashchenko A Turuspekov Y Abugalieva S (2024) Genetic diversity of *Tulipaalberti* and *T.greigii* populations from Kazakhstan based on application of expressed sequence tag simple sequence repeat markers. Plants 13(18): 2667. 10.3390/plants13182667 39339642 PMC11435150

[B80] Yuan L Yan X Chen X Zhu X (2022) The complete chloroplast genome of *Tulipagesneriana* (Liliaceae) and its phylogenetic analysis. Mitochondrial DNA. Part B, Resources 7(7): 1255–1256. 10.1080/23802359.2022.2093676 PMC9275478 35837493

[B81] Zonneveld BJ (2009) The systematic value of nuclear genome size for “all” species of *Tulipa* L. (Liliaceae). Plant Systematics and Evolution 281(1–4): 217–245. 10.1007/s00606-009-0203-7

[B82] Zonneveld BJ de Groot JJ (2012) *Tulipakolbintsevii* Zonn., a new species from Eastern Kazakhstan. Plant Systematics and Evolution 298(7): 1293–1296. 10.1007/s00606-012-0635-3

