# Supplementary material for: ﻿Revisiting the genus Tulipa (Liliaceae) in Kazakhstan, the country with the richest tulip diversity worldwide
Source: PhytoKeys. 2024 Dec 23;250:95–163. doi: 10.3897/phytokeys.250.136736 (PMC11686166; doi:10.3897/phytokeys.250.136736)
Supplement: Supplementary material 1 — Supplementary Information [file phytokeys-250-095_article-136736__-s001.doc]

**Table S1.** Detailed information on taxon, NCBI GenBank accession numbers, and references for the samples used in this study.

| **Species name** | **Subgenus** | **Accession no.** | **Reference** |
| --- | --- | --- | --- |
| *Tulipa alberti* | *Tulipa* | OP279728.1 | Sutula et al. 2024 |
| *Tulipa alberti* | *Tulipa* | OR634827.1 | unpublished |
| *Tulipa altaica* | *Tulipa* | OR634828.1 | unpublished |
| *Tulipa altaica* | *Tulipa* | MW938051.1 | unpublished |
| *Tulipa auliekolica* | *Eriostemones* | PP087901.1 | unpublished |
| *Tulipa auliekolica* | *Eriostemones* | OR687566.1 | unpublished |
| *Tulipa biebersteiniana* | *Eriostemones* | OR699357.1 | unpublished |
| *Tulipa biflora* | *Eriostemones* | HF952957.1 | unpublished |
| *Tulipa biflora* | *Eriostemones* | OR699318.1 | unpublished |
| *Tulipa bifloriformis* | *Eriostemones* | OQ733258.1 | unpublished |
| *Tulipa borszczowii* | *Tulipa* | HF952959.1 | unpublished |
| *Tulipa brachystemon* | *Tulipa* | MG525520.1 | unpublished |
| *Tulipa buhseana* | *Eriostemones* | MG525521.1 | unpublished |
| *Tulipa dasystemon* | *Eriostemones* | MG525522.1 | unpublished |
| *Tulipa dubia* | *Tulipa* | OQ733267.1 | unpublished |
| *Tulipa greigii* | *Tulipa* | OP279724.1 | Sutula et al. 2024 |
| *Tulipa heteropetala* | *Orithyia* | OR678372.1 | Berdimuratova et al.2023 |
| *Tulipa heterophylla* | *Tulipa* | MT923865.1 | unpublished |
| *Tulipa iliensis* | *Tulipa* | MT923867.1 | unpublished |
| *Tulipa ivasczenkoae* | *Tulipa* | OR739613.1 | unpublished |
| *Tulipa kaufmanniana* | *Tulipa* | OP279725.1 | Sutula et al. 2024 |
| *Tulipa kolpakowskiana* | *Tulipa* | MG525525.1 | unpublished |
| *Tulipa korolkowii* | *Tulipa* | HF952966.1 | Christenhusz unp. 2013 |
| *Tulipa lehmanniana* | *Tulipa* | EU912094.1 | Zarrei et al. 2009 |
| *Tulipa lemmersii* | *Tulipa* | MG525526.1 | unpublished |
| *Tulipa orthopoda* | *Eriostemones* | MG525527.1 | unpublished |
| *Tulipa ostrowskiana* | *Tulipa* | OP482247.1 | Sutula et al. 2024 |
| *Tulipa patens* | *Tulipa* | OR634825.1 | Kubentayev unp. 2023 |
| *Tulipa regelii* | *Eriostemones* | MG525530.1 | unpublished |
| *Tulipa sogdiana* | *Eriostemones* | MH555167.1 | unpublished |
| *Tulipa suaveolens* | *Tulipa* | OR699307.1 | unpublished |
| *Tulipa sylvestris* | *Eriostemones* | HF952974.1 | unpublished |
| *Tulipa tarda* | *Eriostemones* | AF485305.1 | Allen et al. 2003 |
| *Tulipa tarda* | *Eriostemones* | MG525532.1 | unpublished |
| *Tulipa turgaica* | *Eriostemones* | PP087900.1 | unpublished |
| *Tulipa turkestanica* | *Eriostemones* | OP279723.1 | Sutula et al. 2024 |
| *Tulipa uniflora* | *Orithyia* | OP630729.1 | Sutula et al. 2024 |
| *Amana edulis* |  | PP708557.1 | unpublished |
| *Erythronium sibiricum* |  | OQ423190.1 | unpublished |

**Table S2.** Summary of *Tulipa* distribution in floristic regions of Kazakhstan and conservation status.

| **No.** | **Species** | **Floristic Regions** | **IUCN RL category** | **Category according to the Red Data Book** **of Kazakhstan** |
| --- | --- | --- | --- | --- |
|  | *Tulipa alberti* Regel | Western Upland, Eastern Upland, Betpak-Dala, Balkhash-Alakol, Dzungarian Alatau, Trans-Ili Kungey Alatau, Chu-Ili range, Karatau, Moiynkum | Near threatened | II |
|  | *Tulipa altaica* Pall. ex Spreng. | Western Upland, Eastern Upland, Zaysan, Betpak-Dala, Altai, Tarbagatai | Least concern | *–* |
|  | *Tulipa annae* J.de Groot & Zonn. | Dzungarian Alatau, Altai | – | *–* |
|  | *Tulipa auliekolica* Perezhogin | Tobol-Ishim, Turgay | – | *–* |
|  | *Tulipa biebersteiniana* Schult.f. | Syrt, Tobol-Ishim, Caspian region, Bukeev, Aktobe, Mugojary, Emba, Turgay, Western Upland, Ulytau, Aral region | *–* | III |
|  | *Tulipa biflora* Pall. | Caspian region, Bukeev, Aktobe, Mugojary, Turgay, Western Upland, Eastern Upland, Karkaraly, Zaysan, Northern Ustyrt, Aral region, Betpak-Dala | – | I |
|  | *Tulipa bifloriformis* Vved. | Kyzylorda, Moiynkum, Turkestan, Kyrgyz Alatau, Karatau, Western Tian Shan | Least concern | *–* |
|  | *Tulipa borszczowii* Regel | Aral region, Kyzylorda, Betpak-Dala, Moiynkum, Kyzylkum, Turkestan | Near threatened | II |
|  | *Tulipa brachystemon* Regel | Dzungarian Alatau | Least concern | II |
|  | *Tulipa buhseana* Boiss. | Turgay, Aral region, Kyzylorda, Betpak-Dala, Moiynkum, Balkhash-Alakol, Turkestan, Trans-Ili Kungey Alatau, Chu-Ili range, Karatau, Western Tian Shan | – | *–* |
|  | *Tulipa dasystemon* (Regel) Regel | Trans-Ili Kungey Alatau, Ketmen Terskey Alatau,  Kyrgyz Alatau | Least concern | *–* |
|  | *Tulipa dasystemonoides* Vved. | Kyrgyz Alatau, Western Tian Shan | – | *–* |
|  | *Tulipa dianae-verettiae* J.de Groot & Zonn. | Altai | Critically Endangered | *–* |
|  | *Tulipa dubia* Vved. | Western Tian Shan | Near threatened | *–* |
|  | *Tulipa greigii* Regel | Moiynkum, Turkestan, Trans-Ili Kungey Alatau, Chu-Ili range, Kyrgyz Alatau, Karatau, Western Tian Shan | Least concern | III |
|  | *Tulipa heteropetala* Ledeb. | Zaysan, Altai, Tarbagatai | Least concern | II |
|  | *Tulipa heterophylla* Regel | Trans-Ili Kungey Alatau, Ketmen Terskey Alatau | Least concern | *–* |
|  | *Tulipa iliensis* Regel | Ketmen Terskey Alatau | Near threatened | *–* |
|  | *Tulipa ivasczenkoae* Epiktetov & Belyalov | Dzungarian Alatau | Critically Endangered | *–* |
|  | *Tulipa jansii* J.J. de Groot & Zonn. | Balkhash-Alakol, Dzungarian Alatau | – | *–* |
|  | *Tulipa kaufmanniana* Regel | Kyrgyz Alatau,  Karatau, Western Tian Shan | Near threatened | III |
|  | *Tulipa kolbintsevii* Zonn. | Dzungarian Alatau | Endangered | *–* |
|  | *Tulipa kolpakowskiana* Regel | Moiynkum, Dzungarian Alatau, Trans-Ili Kungey Alatau,Ketmen Terskey Alatau, Chu-Ili range, Kyrgyz Alatau | Near threatened | III |
|  | *Tulipa korolkowii* Regel | Turkestan, Western Tian Shan | Near threatened | II |
|  | *Tulipa kujukense* J.J. de Groot & Zonn. | Karatau | – | *–* |
|  | *Tulipa lehmanniana* Mercklin | Betpak-Dala, Moiynkum, Balkhash-Alakol, Kyzylkum, Turkestan, Chu-Ili range | Near threatened | I |
|  | *Tulipa lemmersii* Zonn., Peterse & J.de Groot | Western Tian Shan | Vulnerable | *–* |
|  | *Tulipa orthopoda* Vved. | Karatau,Western Tian Shan | Vulnerable | *–* |
|  | *Tulipa ostrowskiana* Regel | Kyrgyz Alatau, Trans-Ili Kungey Alatau | Near threatened | III |
|  | *Tulipa patens* C. Agardh ex Schult. & Schult. f. | Syrt, Tobol-Ishim, Irtysh, Semipalatinsk pine forest, Kokchetav, Mugojary, Turgay, Western Upland, Ulytau, Zaysan, Eastern Upland, Karkaraly, Altai, Tarbagatai | *–* | III |
|  | *Tulipa regelii* Krasn. | Chu-Ili range, Balkhash-Alakol | Endangered | II |
|  | *Tulipa salsola* Rukšāns & Zubov | Dzungarian Alatau | – | *–* |
|  | *Tulipa sogdiana* Bunge | Northern Ustyrt, Mangyshlak, Southern Ustyrt, Kyzylkum | **–** | *–* |
|  | *Tulipa suaveolens* Roth | Syrt, Tobol-Ishim, Caspian region, Bukeev, Aktobe, Mugojary, Emba, Turgay, Western Upland, Ulytau, Aral region | – | III |
|  | *Tulipa tarda* Stapf | Trans-Ili Kungey Alatau | Least concern | II |
|  | *Tulipa tetraphylla* Regel | Trans-Ili Kungey Alatau, Ketmen Terskey Alatau | Least concern | *–* |
|  | *Tulipa turgaica* Perezhogin | Aktobe, Turgay | – | *–* |
|  | *Tulipa turkestanica* Regel | Karatau, Western Tian Shan | Least concern | *–* |
|  | *Tulipa × tschimganica* Botschantz. | Western Tian Shan | **–** | *–* |
|  | *Tulipa uniflora* (L.) Besser ex Baker | Zaysan, Altai, Tarbagatai | Near threatened | III |
|  | *Tulipa zenaidae* Vved. | Kyrgyz Alatau | Vulnerable | II |

**S1.** Species examined in the study of the distribution of tulips in Kazakhstan.

***Tulipa alberti* Regel**

**Specimens examined:—**WESTERN UPLAND. Near Zhezkazgan, along the Atasu-Zhezkazgan motorway, 23 Apr 2021, *Kubentayev and Alibekov s.n.* (NUR!); near Zhezkazgan, along the Zhezkazgan-Kyzylorda motorway, 25 Apr 2021, *Kubentayev and Alibekov s.n.* (NUR!); near Zhezkazgan airport, Zhezkazgan-Pavlodar motorway, 23 Apr 2023, *Kubentayev et al. s.n.* (NUR!); near dam Darat, 17 Sept 2024, *Satekov s.n.* (personal observation). BETPAK-DALA. Near Gulshat, Targyl mountain, 23 Apr 2021, *Kubentayev and Alibekov s.n.* (NUR!); Saryshagan-Khantau road, 15 Apr 2022, *Kubentayev and Alibekov s.n.* (NUR!); Sarysu river, near the as-bulak tract, 29 Sep 1934, *Krasheninnikov and Vitovt s.n.* (LE!); 120 km south of Karsakpay, near the Ak-mulla river, 16 Apr 1940, *Leont’ev s.n.* (MW 0815379!); Betpak-dala, Kogashyk tract, a hillside 3 km north-west of the meteorological station, 6 May 1960, *Ospanova s.n.* (LE!); Betpak-dala, Kogashyk tract, plain 10 km west of the meteorological station, 13 Jun 1960 *Grubov s.n.* (LE!); near the Kyzyl-Zhingil tract on the Sarysu river, 17 Jul 1914, *Semenov* *s.n.* (TK!); Karazhar tract, at the confluence of the Sarysu and Kara-kengir rivers, 11 Jul 1914, *Semenov s.n.* (TK!); Kendyrlik cemetery, 29 May 1936, *Mironov and Pazij s.n*. (TASH!); near the branching point of the Sarysu and Kara-Kengir rivers, 23 Apr 2023, *Kubentayev et al. s.n.* (NUR!). EASTERN UPLAND. On the Balkhash-Karaganda motorway, Bektau-ata mountains, 300 km south of Karaganda, 30 Apr 2008, *Ivashchenko s.n.*(personal observation). TRANS-ILI KUNGEY ALATAU. Kurty river basin, Zhalpak-tau tract, 2 Apr 1909, (LE!); Chambaytal Gorge, 9 May 1957*, Alukina s.n.,*(TK). CHU-ILI RANGE. Mount Anrakhay, Kosharsay, 25 Jun 1968, *Puchkova s.n*. (TASH!); Almaly-sai tract, 5 May 1951, *Pavlov s.n.* (MW 0815410!);(TK); same loc., 4 Apr 1951, *Pavlov s.n*. (MW 0815409!); Chu-Ili mountains, near Khantau, 13 Jun 1914, *Titov s.n*. (LE!); Kopala river basin, Anrakhay tract, 10 May 1909, *Nedzvedskij s.n.* (LE!); slopes of Chu-Ili mountains, Aynabulak station, 22 Apr 1939, *Pavlov s.n.* (MW 0815408!); (MW 0815407!); (KNU!); near the village of Mirnyy, the Mirnyy-Akbakai road, 12 Apr 2024, *Kubentayev et al.s.n.* (NUR!). KARATAU. Karatau mountains, north-western slope, Kara-say tract, 12 May 1936, *Pjataeva s.n.* (TASH!); same loc., 20 May 1935, *Pjataeva s.n.* (TASH!); same loc., 23 May 1936, *Pjataeva s.n*. (TASH!); south-western slope, Kara-say tract, 14 May 1934, *Pjataeva s.n.* (TASH!); (MW 0815403!); the summit of Su-Alma, 14 May 1930, *Lipschitz s.n.* (TASH!); (MW 0815400!); (MW 0815397!); same loc., 8 Aug 1934, *Lipschitz s.n.* (LE!); Ush-ozen tract, 22 Apr 1930, *Lipschitz s.n.* (TASH!); (MW 0815393!); (MW 0815394!); same loc., 8 Apr 1930,  *Lipschitz s.n.* (LE!); (MW 0815402!); (MW 0815395!); Say Boyaldyr, Zhaman-terek mountain, 19 Aug 1930, *Gomolitsky s.n.* (TASH!); Sozak Biresek river basin, 11 Jul 1930, *Gomolitsky s.n.* (TASH!); Orta-tau tract, 13 May 1934, *Pjataeva s.n*. (TASH!); Zhylagan Ata cave, 17 May 1934, *Pjataeva s.n*. (TASH!); Kondely-say tract, 13 May 1934, *Pjataeva s.n*. (TASH!); Tekshe-tau plateau, May 1934, *Pjataeva s.n*. (TASH!); Mynzhylky massif, Almaly-say gorge, 16 May 1977, *Kamelin et al. s.n*. (LE!); Mynzhylky mountains, 5 Jul 1939, *Kuznetsov s.n.* (KNU); same loc., 8 May 1939, *Pavlov s.n.* (MW 0815406!); (MW 0815396!); Bala-Sauskandyk mountain, in the area of Koskul (Zhamankol) lake, 13 May 1977, *Kamelin et al. s.n.* (LE!); Manzhytas Pass [Mangytai], 1 Jun 1958, *Neustrueva et al.* s.n. (LE!); Bol’shoy Ak-tau ridge, near Chul-say gorge, 22 May 1936, *Chilikina s.n.* (MW 0815405!); (MW 0815399!); Biresek river valley, 20 May 1952, *Parfent’eva s.n.* (MW 0815404!); Karatau mountains, dzhon Bayzhansay, 16 Jul 1949, *Pavlov s.n.* (MW 0815401!); Ush-Ozek [Ush-Uzjuk] tract, 29 Apr 1930, *Lipschitz s.n.* (MW 0815392!); Karatau mountains, 5 May 1931, *Tojibaev s.n.* (MW 0815376!); Eastern part of the Karatau mountains, near the Kazdegeres hillock, 11 May 1936, *Chilikina s.n.* (MW 0815398!).

***Tulipa altaica* Pall. ex Spreng.**

**Specimens examined:—**EASTERN UPLAND. Bektauata mountains, 22 Apr 2021, *Kubentayev and Alibekov s.n.* (NUR!); between Semey and Ulyguz station, 30 Apr 1912, *Shishkin s.n.* (LE!); (TK!); northern slope of mount Ushkara, 16 May 1914*, Schipczinsky s.n.* (LE!); the road between Sergiopol’ to Zhuz-Agach, 17 Apr 1902, *Saposhnikov s.n.* (LE!). WESTERN UPLAND. Near Atasu, 25 Apr 2021, *Kubentayev and Alibekov s.n.* (NUR!); Bol’shoy Koksengir mountains, 1 May 1959, *Rachkovskaya s.n*. (LE!); Kokshetau mountains, 14 May 1957, *Karamysheva s.n.* (LE!); Ortau mountains, near the Sarybulak wintering ground, 22 Apr 2023, *Kubentayev et al. s.n.* (NUR!). BETPAK-DALA. Northern shore of Balkhash, Tar-Tubek peninsula, 23 Apr 1885, *Nikolsky s.n.* (LE!). ALTAI. Delbegetey mountains, 17 Sep 1928, *Iljin and Heinrichson s.n.* (LE!); Narym ridge, near the Kaznakovskaya crossing on the Bukhtarma reservoir, 5 May 2001, *Dyachenko s.n.* (ALTB 1100006790!); Kurchum ridge, Kukumbay, 42 km from Kurchum, 6 May 2001, *Dyachenko s.n.* (ALTB 1100006798!), Narym ridge, near Kainar, 13 May 2014, *Kubentayev s.n.* (NUR!); Kalzhir valley, Kalzhir river, Alekseyevsky village, 13 Apr 1908, *Keller s.n.* (LE!). TARBAGATAI. Bol’shoy Zhemeney river, 1 Jul 1914, *Shishkin s.n.* (LE!); the watershed of the Bol’shoy and Malyy Zhemeney rivers, 6 Jul 1930, *Goncharov and Borisova s.n.* (LE!); In campestribus montium Aktschauly et Tarbagatai ad torrentem Terekty, 1840, *Karelin and Kirilov s.n.* (LE!); along the left bank of the Terekty river, 2 Jun 1914, *Shishkin* *sn.* (TK!). ZAYSAN. Between the villages of Ivanovskoye-Kokpekty, 16 Jun 1914, *Saposhnikov and Genina s.n*. (LE!); Kalzhir valley, Kalzhir river, Ayna-bulak, 17 Jul 1908, *Keller s.n*. (LE!); the town of Zaysan, 17 Jun 1908, *Keller s.n*. (LE!); between the sandy headland on lake Zaysan and Tolagay, 22 May 1914, *Shishkin s.n.* (LE!); Zaysan Basin, Kein-Kerish mountain, 26 May 2015, *Kubentayev s.n.* (NUR!); in the hills of Araltobe, near Kokpekty, 22 Apr 1863, *Potanin s.n.* (TK!);(LE!); Chakylmys [Shakelmes] mountain, 22 Apr 1863, *Potanin s.n.* (TK!).

***Tulipa annae* J. de Groot & Zonn.**

**Specimens examined:—**ALTAI. Altai. Marble Pass 2008, *J.J. de Groot s.n.* (L 3986814).

***Tulipa auliekolica* Perezhogin**

**Specimens examined:—**TOBOL-ISHIM. The track is 15 km from the Karamendy turn-off, 25 Apr 2009, *Perezhogin et al. s.n.* (KSPI!); 10 km from the Karamendy turn-off, on the Karamendy-Auliyekol road, 27 Apr 2023, *Kubentayev et al. s.n.* (NUR!); Kokterek tract, 2 May 2010, *Perezhogin et al. s.n.* (KSPI!); Naurzum Nature Reserve, 24 Apr 2011, *Perezhogin et al. s.n.* (LE!). TURGAY. The sands of Akkum, 16 Jul 2009, *Perezhogin et al. s.n.* (KSPI!); near Kumkeshu, 2 May 2010, *Perezhogin et al. s.n.* (KSPI!); 20 km north of the Akkum sands, 3 May 2014, *Perezhogin and Kurlov s.n.* (LE!); Damdy river, 24 Apr 2011, *Perezhogin and Borodulina s.n.* (KSPI!).

***Tulipa biebersteiniana* Schult. & Schult. f.**

**Specimens examined:—**SYRT. Near Uralsk, in the valley of the Krutoy river, 19 Apr 1913, *Borodin s.n.* (LE!); near Uralsk, on the slope of the chalk hills, 25 Apr 1923, *Verushkin s.n*. (LE!); slopes along the road from Sholaksay to lake Zharkol, 17 Jun 1908, *Krasheninnikov s.n.* (LE!); Sokolovka village, chalk mountain, 23 May 1924, *s.n.* (LE!); near Uralsk, in a government garden, 1 May 1922, *Larin s.n.* (LE!); near Uralsk, behind Aniskino lake, 22 May 1925, *Pojarkova s.n.* (LE!); Uralsk, transshipment grove, 30 Apr 1924, *Musatova s.n.* (LE!). TOBOL-ISHIM. Arakaragay forest, 8 May 1921, *Pavlov s.n.* (US 2090752!); (BRNU 221979!); (LE!); Tobol river valley near Kostanay, 5 Jul 1921, *Pavlov and Pavlova s.n.* (LE!); near Nadezhdinka station, Toguzak river, 9 Jun 1913, *Korotky and Lebedeva s.n.* (LE!); near Valer’yanovka, the Tobol river, 20 Jul 1913, *Korotky and Lebedeva s.n.* (LE!); 2nd Naurzum parish, plateau on the right side of the Karateke river, 27 Jul 1908, *Krasheninnikov s.n.* (LE!); 2nd Naurzum parish, steppe on the edge of the Naurzum bog, 26 May 1909, *Savich and Kutscherovskaya s.n.* (LE!); steppe near lake Medet, 25 km from Kostanay, 8 Jun 1908, *Krasheninnikov s.n.* (LE!); 1st Naurzum parish, steppe on the edge of the Naurzum boron, 30 May 1909, *Kutscherovskaya s.n.* (LE!); 2nd Naurzum parish, eastern shore of lake Alakol, 19 Jun 1908, *Krasheninnikov s.n.* (LE!); 2nd Naurzum parish, Chulakskaya farm, to Zharkol lake, 17 Jun 1908, *Krasheninnikov s.n.* (LE!); 2nd Naurzum parish, Karagaily mountains, 22 Jun 1908, *Krasheninnikov s.n.* (LE!); Naurzum Nature reserve, near Kotantal lake, 19 May 1936, *Voronov s.n*. (MW 0815428!); near lake Karakol, 6 May 1929, *Kalabukh s.n.* (MW 0815427!); same loc., 30 Apr 1929, *Kalabukh s.n.* (MW 0815425!); Naurzum Nature reserve, south of Yeginbulak, 4 May 1935, *Levitsky s.n*. (MW 0815426!); in the Tobol river valley near Kostanay, 7 May 1921, *Kulikova s.n.* (MW 0815424!). CASPIAN REGION. Near lake Inder, 16 Apr 1870, *Smirnov s.n*. (LE!); along the Sagyz river, 7 May 1924, *Serova s.n.* (LE!); Mount Zhirentau, 1 Aug 1927, *Rozhevitz and Zhezhel s.n.* (LE!); near Zhanibek, 25 May 1996, *Bykov s.n.* (MHA 0010125!). AKTOBE. Izembet station, 18 Apr 1908, *Noskov s.n.* (LE!); (MW 0291250!); near Temir, 22 Apr 1908, *Borodin et al.s.n.* (LE!), Kandyagash station, 22 Apr 1913, *Popov s.n.* (TASH!). BUKEEV. Near Safonovka, near the Zormutinsky society. 26 Apr 1927, *Dmitriev s.n.* (MW 0291254!). MUGOJARY. Mugodzhary, Kara-bulak tract, near railway station No. 59, 19 May 1927, *Rusanov* *s.n.* (LE!); Mugodzhary, Birshoghyr station, 5 Apr 1910, *Androssov s.n.* (LE!); same loc., 30 Apr 1930, *Dombrovsky s.n.* (MW 0815419!); (TASH!); Temir zonal station, near Zhuryn station, 11 May 1933, *Voronov s.n.* (MW 0815418!); Mugodzhary, near the Birshoghyr coal mines, 29 May 1921, *Korovin s.n.* (TASH!); Junction No. 57, Kyrgyzstan-Mugodzhary, 21 Apr 1929, *Vvedensky s.n.* (TASH!). TURGAY. Steppe between the lakes Tomalakkol and Shubarkol, 29 Apr 1914, *Desyatova s.n.* (LE!); 20 km west of Aksuat, 18 May 1945, *Voronov s.n.* (MW 0815423!); Arkaly-sai river, 11 May 1909, *Savich and Kutscherovskaya s.n.* (LE!); near lake Malyy Zhalanash, 28 Apr 1898, *Krjukov s.n.* (MW 0815421!); near Amangeldy, near the bridge over the Karynsaldy river, 24 Apr 2023, *Kubentayev et al. s.n.* (NUR!). WESTERN UPLAND. Zharlykol lake, 3 Jun 1914, *Semenov s.n.* (LE!); near Atbasar, 18 May 1886, *Shadrin s.n.* (TK!). ULUTAU. Near Ulytau, Ulytau-Arkalyk road, along the bank of the Zhetykyz river, 23 Apr 2023, *Kubentayev et al. s.n.* (NUR!). ARAL REGION. Malyye Barsuki, along the ravine bed, 11 May 1931, *Serova s.n.* (MW 0815417!); 15 km north-east of Shalkar station, Kalzhyr river, 30 May 1927, *Spiridonov s.n.* (LE!); Bol’shiye Barsuki, 20 Apr 1904, *Dubjansky s.n.* (LE!); beginning of the Bol’shiye Barsuki sands from Shalkar station, 17 Apr 1907, *Androssov s.n.* (LE!); Bol’shiye Barsuki sands, 12 km north-east of Karashokat, 24 Apr 1930, *Gozhev et al. s.n.* (LE!); between Bolshiye and Malyye Barsuki, the shore of Lake Karaguz, 29 Apr 1914, *Desyatova s.n.* (LE!).

***Tulipa biflora* Pall*.***

**Specimens examined:—**BUKEEV. Mayachnyy hillock, near Safonovka, 26 Apr 1927, *Dmitriev s.n.* (MW 0291292!). CASPIAN REGION. Near the near Karaabinskaja [Karaobinskaja] russian-kyrghyz [russian-kazakh] school, 23 Apr 1895, *Kulyasov s.n.* (MW 0291294!); near Dossor, 28 Apr 2022, *Alibekov s.n.* (NUR!). ARAL REGION. “Aral sea” station, 10 Apr 1940, *Leont’ev s.n.* (MW 0815374!); Northern Priaral’e, 20 km north-west of Aral’sk, 30 May 1990, *Ishkov s.n.* (personal observation); Raimskaya mountain, Kamyshly-bash station, 28 Apr 1909, *Stuckenberg s.n.* (LE!). AKTOBE. Izembet station, 18 Apr 1908, *Noskov s.n.* (MW 0291303!); same loc., 18 May 1908, *Noskov s.n.*(TK!); north-east of the village of Oiyl, Akshatau, 7 May 1969, *Tscherkassova s.n.* (MW0815432!). MUGOJARY. Kirgizskaya station of the Tashkent railway, 30 Apr 1918, *Sovetkina s.n.* (TASH!). TURGAY. 3 km south-east of Aksuat, 27 Apr 1945, *Voronov s.n.* (MW 0815433!); Altyn Dala reserve, a section of the Tasynkuma, 16 Jul 2007, *Ivashchenko s.n.* (personal observation); Altyn Dala reserve, Uly-Zhylanshyk site, 12 Jun 2007, *Ivashchenko s.n.* (personal observation); same loc., 8 Jul 2007, *Ivashchenko s.n.* (personal observation); Altyn Dala reserve, a section of Sarykopa, near lake Lysoye, 16 Jul 2007, *Ivashchenko s.n.* (personal observation); Altyn Dala reserve, Uly-Zhylanshyk site, 4 km north-west of Rakhmet, 8 Jul 2007 *Ivashchenko s.n.* (personal observation). WESTERN UPLAND. Foothill plain near the western part of Kokshetau, 25 Apr 1957, *Rachkovskaya s.n.* (LE!); 28 km south-east of Zhanaarka, 2 May 1958, *Bespalova s.n.* (LE!); steppe south of the Zhylandy river, 6 Jul 1908, *Kapelkin s.n.* (LE!); Kokshetau, near the Tersakkan river, 22 May 1842, *Schrenk s.n.* (LE!); near Shubarkol, 25 Apr 1988 *Mikhailov s n.* (KG!). EASTERN UPLAND. Bektauata mountains, 8 Jun 1988, *Mikhailov s n.* (KG!); same loc., 15 Apr 2022, *Kubentayev and Alibekov s.n.* (NUR!); same loc., 22 Apr 2021, *Kubentayev and Alibekov s.n.* (NUR!); same loc. 30 Apr 2008, *Ivashchenko s.n.* (personal observation); same loc., 20 Apr 2023, *Kubentayev et al. s.n.* (NUR!); Konyrkulzha mountains, 13 Apr 2022, *Kubentayev and Alibekov s.n.* (NUR!); Sergiopol’, 17 Apr 1932, *Karelin s.n.* (LE!); from Sergiopol’ to Zhuz-Agach, 17 Apr 1902, *Saposhnikov s.n.* (TK!). KARKARALY. Karkaraly, 25 May 1890, *Korzhinsky s.n*. (LE!). ZAYSAN. Zaysan basin, Kein-Kerish mountain, 26 May 2015, *Kubentayev s.n.* (NUR!); same loc., 13 May 2001, *Dyachenko s.n.* (ALTB 1100007599!); Black Irtysh river valley, Ashutas massif, 10 May 2001, *Dyachenko s.n.* (ALTB 1100007573!). NORTHERN USTYURT. Northern shore of the Aral Sea, between Karatamak and Koshkar-ata, 15 Apr 1915, *Bukinich s.n*. (LE!); Mount Donyztau, 5 May 2018, *Kuanbai s.n.* (NUR!). BETPAK-DALA. Sarysu sands, near the bridge, Sarysu tributary, 4 Apr 2024,  *Kubentayev et al*. *s.n*. (NUR!).

***Tulipa bifloriformis* Vved.**

**Specimens examined:—**TURKESTAN. Kuzdy-Togay tract, 2 km from the Syrdarya river, Alka-kul-kum [Alkakolkum] sands, 16 Apr 1962, *Tajzhanov s.n.* (TASH!); Keles massif, Kuzdy-Togay tract, 3 km from the Syrdarya river floodplain, Alakul kum sands, 16 Apr 1962, *Pjataeva s.n.* (TASH!); Connector behind station No. 1 from Arys station, 1935, *Botschantzeva s.n.* (TASH!). KARATAU. Karatau mountains, Karasay tract, 10 May 1935, *Pjataeva s.n.* (TASH!); between the village of Sholak-Korgan and Sozak, 14 May 1936, *Mironov and Pazij s.n.* (TASH!); Karatau, 1981, *Lyashenko s.n.* (AA!); same loc., 29 Apr 1935, *Tekut*’*ev s.n.* (MW 0815434!); Karatau ridge, Mynzhylky massif, Sarymsakty gorge, 10 Jun 1981, *Bajtenov s.n.* (AA!); Shayanbi pass, 30 Apr 1930, *Lipschitz s.n.* (MW 0815798!); (MW 0815799!); chalk mountains Aktau, 10 May 1936, *Chilikina s.n.* (MW 0815806!); Mynzhylky mountains, 8 May 1939, *Pavlov s.n.* (MW 0815807!). WESTERN TIAN SHAN. Middle part of the Keles river basin, Kaplanbek tract, 2 Apr 1921, *Abolin s.n.* (TASH!); Darbaza, a hilly rise between mount Kzyl-Choka [Kyzylshoky] and Chemyr-bay-say [Shymyrbay say], May 1940, *Gromakov s.n.* (TASH!); 1 km south of Darbaza state farm, 28 May 1940, *Nazarenko s.n*. (TASH!); near Kaplanbek, 30 Mar 1956, *Korotkova s.n.* (TASH!); same loc., 26 Mar 1957, *Adylov s.n.* (TASH!); same loc., 28 Mar 1922, *Vvdensky s.n*. (TASH!); Shymkent, 27 Mar 1932, *Zhugina s.n.* (TASH!); middle part of the Keles river basin, Chernyayevka [Zhibek Zholy] village, 16 Apr 1921, *Abolin s.n.* (TASH!); Shymkent, Kabulsay station, 10 May 1920, *Popov s.n.* (TASH!); the slope of the Alymtau mountain, 10 Apr 1916, *Kultiassov s.n.* (TASH!); Mount Kazygurt, 15 Jul 1922, *Baranov s.n.* (TASH!); same loc., 18 May 1996, *Pratov s.n.* (TASH!); Kempir- Darbaza, 6 km from Kabulsay station, 3 May 1924, *Mokeeva s.n.* (TASH!); near Tashkent, Darbaza station, 11 Mar 1940, *Lepeshkin and Pazij s.n.* (TASH!); Montaytas station, 29 Mart 1911, *Dimo et al. s.n.* (TASH!); Zhabagly mountains, 2 Jun 1924, *Sovetkina s.n.* (TASH!); middle part of the Keles river basin, Kaplanbek tract, 9 Apr 1921, *Abolin s.n.* (TASH!); Stolovaya mountain, 14 Apr 1960, (TASH!); Ulken Kaindy gorge, 14 May 2021, *Manabaeva s.n.* (NUR!); Aksu-Zhabagly Nature Reserve, 1933, *Linczevsky s.n.* (AA!); 52 km south-east of Shymkent, Karzhantau ridge, 25 Apr 1979, *Pershina s.n.* (MW 0815435!); Kuyuk pass, 11 Apr 2024, *Kubentayev et al*. *s.n*. (NUR!); slope of Mashat mountains, 9 Apr 2024, *Kubentayev et al*. *s.n*. (NUR!). KYRGYZ ALATAU. Merke river gorge, 2 May 1976, *Harlamova s.n.* (AA!); Kyrgyz ridge, 12 km south-east of Dzhambul, 6 May 1918, *Fissyun s.n.* (AA!); near Aulie-ata [Taraz], the Tekturmas hill, 3 May 1916, *Rajkova s.n.* (TASH!).

***Tulipa borszczowii* Regel**

**Specimens examined:—**KYZYLORDA. Near Tyuratam station, 25 Apr 1930, *Dombrovsky s.n.* (US 3006780!); (BRNU 472452!); (AA!); (MW 0815438!); (LE!); (TK!); (TASH!); Turgai Tertiary plateau, 90 km north of Kyzylorda, 9 Apr 1948, *Gamayunova s.n.* (AA!); Turgay Tertiary plateau, 150 km north of Kyzylorda, 28 Apr 1948, *Gamayunova s.n.* (AA!); Tertiary plateau, sands 18 km south of lake Aryskol, 28 Apr 1948, *Gamayunova s.n.* (AA!); Zhosaly station, 3 Apr 1909, *Stuckenberg s.n.* (LE!); lower reaches of the Sarysu river, Taylakkol lake, 18 May 1914, *Krasheninnikov s.n.* (LE!); 25 km north of Kyzylorda, 18 May 1990, *Yashchenko s.n.,* between Kyzylorda and Telikol lake and Saray tract, 15 May 1914, *Krasheninnikov s.n.* (LE!); sands between Tyuratam and Baykozha, 14 Jun 1921, *Sprygin s.n.* (LE!); Kyzylorda-Zhezkazgan road, 18 Apr 2022, *Kubentaev and Alibekov s.n.* (NUR!); between Telikol and Arys, 14 Apr 1908, *Skalov s.n.* (LE!). ARAL REGION. Sands near Aral’sk station, 22 Apr 1910, *Androssov s.n.* (BRNU 092133!); (BRNU 681634!); (AA!); (MW 0815441!); (MW 0815439!); (LE!); (TK!); (TASH!); Priaralie Karakum, Maymak tract, 24 May 1957, *Lushpa s.n.* (AA!); road 110 km south-east of Aralsk, 4 Jun 1974, *Baranova s.n.* (MW0815442!); Priaral’skiye Karakumy, Koskempir tract, 26 Apr 1958, Rothschild s.n. (LE!); Priaral’e, Zhuankum desert, 30 km east of Kaukey, 20 May 1990, *Yashchenko* s.n. (personal observation); Kyzylzhar, 40 km from the mouth of the Syrdarya, 20 Apr 1921, *Lorenz s.n.* (MW 0815440!); Mount Raimskaya (old fortification), 28 Apr 1909, *Stuckenberg s.n.,* (LE!); Bek Bauly [Bekbauyl] station, 27 Mar 1909, *Stuckenberg s.n.* (LE!); Kamystybas station, 19 May 1914 *Titov s.n.* (LE!); same loc., 2 May 1931, *Linczevsky s.n.* (TASH!); Priaral’e, 20 km east of Kulan, near the Mausoleum, 18 Sep 1999, *Ivashchenko s.n.* (personal observation); between Sapak and Kamyshlybash [Kamystybas] stations, 16 Jun 1904, *Fedtschenko s.n.* (LE!); between Sappak [Sapak] and Bekbauli [Bekbauyl], 16 Jun 1904, *Fedtschenko s.n.* (LE!); in the ravines near Raim, 26 Apr 1849, *Butakov s.n.* (LE!); Kazaly, Syrdarya, 1885, *Regel s.n.* (LE!); Priaral’e, from Novyy Karateren to Kazalinsk, between Bugun and Amanotkel, 23 Sep 1999, *Ivashchenko s.n.* (personal observation); Bolshoe Saryshyganak bay, 30 May 1921, *Rajkova s.n.* (TASH!). BETPAK-DALA. Kendyrlik cemetery, 27 May 1936, *Mironov and Pazij s.n.* (TASH!); MOIYNKUM. Togusken upland, 12 Jun 1947, *Yakimova s.n.* (TASH!); north of the Cholak-espe well, 21 May 1936, *Mironov and Pazij s.n.* (TASH!). KYZYLKUM. South-eastern Kyzylkum, 6 km south-west of the Shardara dam, 19 Apr 1965, *Makarchuk s.n.* (TASH!). TURKESTAN. On the road between the villages of Chinaz and Shardara, 7 Apr 1961, *Adylov s.n.* (TASH!); Alka-kol sands, 28 Mar 1947, *Vernik s.n.* (TASH!); Right bank of Syrdarya river, 96 km north of Ospan Kopyr bridge, 25 Apr 1948, *Gamayunova s.n.* (AA!).

***Tulipa brachystemon* Regel**

**Specimens examined:—**DZUNGARIAN ALATAU. Altyn-Emel experimental field, near Altyn-Emel pass, 1915, *Agronom s.n.* (TASH!); Altyn-Emel ridge, Tulkuli mountains, Tulkuli river gorge, 30 Jun 1956, *Goloskokov s.n*. (AA!); (LE!); Koturkain mountains, near the Chagan peaks, 27 May 1959, *Goloskokov s.n.* (AA!); foothills near Kapal, 8 Apr 1908, *Schipczinsky s.n.* (LE!); Shengeldy, 18 Apr 1877, *Regel s.n.* (LE!).

***Tulipa buhseana* Boiss.**

**Specimens examined:—**TURGAY. Near Malyy Zhalanash lake, 26 Apr 1939, *Vvedensky s.n.* (MW 0815454!). ARAL REGION. Aral sea, Barsakelmes island, 31 Jul 1921, *Rajkova s.n.* (TASH!); Contu station, 13 Apr 1914, *Sprygin s.n.* (TASH!). KYZYLORDA. Near Birkazan, 18 Apr 2022, *Kubentayev and Alibekov s.n.* (NUR!); Tyuratam station, 26 Apr 1930, *Dombrovsky s.n.* (MW 0815453!); (TASH!). BETPAK-DALA. Saksauldala, 21 Apr 1976, *Orazova s.n.* (AA!); Central Betpak-Dala, Kogashyk tract, 9 Apr 1931, *Rubtzov s.n*. (AA!); Balkhash lake, Saryshagan, 23 May 1945, *Bajtenov s.n*. (AA!); near Gulshat, close to lake Balkhash, 22 Apr 2021, *Kubentayev and Alibekov s.n.* (NUR!); near Gulshat, Targyl mountain, 23 Apr 2021, *Kubentayev and Alibekov s.n.* (NUR!); same loc., 14 Apr 2022, *Kubentayev and Alibekov s.n.* (NUR!); Saryshagan-Khantau road, 15 Apr 2022, *Kubentayev and Alibekov s.n.* (NUR!); same loc., 15 Apr 2021, *Kubentayev and Alibekov s.n.* (NUR!); near “Pribalkhashstroy”, lake Balkhash, Turangylyk bay, 6 May 1936, *Skabunov s.n.* (LE!); Kendyrlik cemetery, 10 Sep 1934, *Mironov s.n.* (TASH!); Saryarka, 44 km north of the Shiely-bulak well, 1935,  *Vvedensky s.n.* (TASH!); Sarysu sands, near the bridge, Sarysu tributary, 4 Apr 2024,  *Kubentayev et al*. *s.n*. (NUR!); Targyl mountain, 13 Apr 2024, *Kubentayev et al*. *s.n*. (NUR!). MOIYNKUM. Moyinkum sands, Yukalgan well, Chu river, 28 May 1926, *Drobov and Gomolitsky s.n.* (TASH!); south-eastern Betpak-Dala, Saksauldala, between Zhambyl and Khantau mountains, 22 Apr 1976, *Orazova and Fissjun s.n.* (US 3008062!); (BRNU 489554!); (TK!); (MW 0815450!). BALKHASH-ALAKOL. Right bank of the Ili river, 5-6 km from Bakanas, 12 Apr 1946, *Gvozdeva s.n.* (AA!); Pribalkhash’e, right bank of the Ili river, south of Bakanas, 19 May 1969, *Lushpa s.n.* (AA!); right bank of the Ili river, near Bakanas, 6 Apr 1913, *Gvozdeva and Kolesnikov s.n.* (AA!). TURKESTAN. Near Takyrkol, 5 Apr 2024, *Kubentayev et al. s.n.* (NUR!) near Tomenaryk, Zhanakorgan-Shiely road, 5 Apr 2024, *Kubentayev et al*. *s.n*. (NUR!). TRANS-ILI KUNGEY ALATAU. 2 km from the village of Degeres, 8 Apr 2019, *Veselova s.n.* (NUR!); Toraygyr ridge, 19 Apr 1942, *Lazarenko s.n.* (AA!). CHU-ILI RANGE. Near Chokpar station, 23 Apr 1949, *Botschantzev s.n.* (TASH!); Almaly-say tract, 5 May 1951, *Pavlov s.n.* (TK!); same loc., 5 May 1951, *Pavlov s.n.* (AA!); same loc., 5 May 1951, *Pavlov s.n.* (MW 0815817!); slopes of the Chu-Ili mountains, beyond Otar, 22 Apr 1939, *Pavlov s.n.* (AA!);(MW 0815451!); foothills of the Chu-Ili mountains, 16 Apr 1976, *Orazova s.n.* (AA!); near Kiyakhty, 12 Apr 2024, *Kubentayev et al*. *s.n*. (NUR!); Chu-Ili mountains, stony slopes between Ala-Aigyr and Kulakshyn stations,7 Jun 1931, *Botschantzev s.n.* (TASH!); near the village of Mirnyy, the Mirnyy-Akbakay road, 12 Apr 2024, *Kubentayev et al. s.n.* (NUR!). KARATAU. Karatau mountains, 8 Apr 1930, *Lipschitz s.n.* (MW 0815447!). WESTERN TIAN SHAN. Alymtau mountain, 8 Apr 2024, *Kubentayev et al*. *s.n*. (NUR!); Kuyuk pass, Kuyuk mountains, 1 May 1939, *Pavlov s.n.* (AA!); Zhilga station, 24 Apr 1914, *Minkwitz s.n.* (LE!).

***Tulipa dasystemon* (Regel) Regel**

**Specimens examined:—** TRANS-ILI KUNGEY ALATAU. Korday district, Argayty [Yrghayty] river, 18 Jun 1926, *Abolin s.n.* (TASH!); north-east of Krasnogorskoye, Ak-tas ridge, 25 May 1942, *Goloskokov s.n*. (AA!); river Malaya Almatynka, 13 May 1916*, Abolin s.n.* (TASH!); same loc., 13 May 1915, *Abolin s.n.* (TASH!); lake Issyk, 1 Jun 1931, *Botschantzev s.n.* (TASH!); Kumbel tract, between the Bol’shaya and Malaya Almatinka gorges, Aug 1924, *Evstifeev s.n*. (TASH!); near Vernoy, May 1917, *Titov s.n*. (TASH!); Suuktobe mountains, the gorge of the Zhiren-Aigyr river, 20 May 1976, *Orazova and Fissjun s.n.* (BRNU 489552!); (AA!); gorge of the Malaya Almatinka river, Medeo, 6 May 2019, *Skvortsov s.n.* (NUR!); north Almaty, 20 May 1980, *Matthews and Victoria s.n.* (E 19802362!); Suuktobe mountain, the gorge of the Zhiren-Aigyr river, 20 Apr 1976, *Orazova and Fissjun s.n.* (TK!); (BRNU 489552!);(US 3008063!); gorge of the Malaya Almatinka river, Aman-Zhailau, 11 Jun 1936, *Goloskokov s.n.* (AA!); Alatau mountains, near the Battery Gap, 29 Apr 1917, *Harin s.n.* (AA!); gorge of the Malaya Almatinka river, 2 Jun 1936, *Goloskokov s.n.* (AA!); valley of the Bol’shaya Almatinka river, 21 May 1953, *Pavlov s.n.* (AA!); near Almaty Lake, valley of the Bol’shaya Almatinka river, 6 Jun 1935, *Kubanskaya s.n*. (AA!); near Almaty, near the “Vorota”, 8 Jun 1936, *Rubtzov s.n.* (AA!); Chin-Turgen gorge, 16 May 1967, *Tzagolova s.n.* (AA!); Aksay gorge, 28 May 1941, *Polyakov s.n.* (AA!); Kurmekty, 24 Apr 1942, *Lazarenko s.n.* (AA!); Kastek river gorge, Suyk tau peak, 27 May 1936, *Linczevsky s.n.* (AA!); Suyk tau mountain, near Kastek, 6 Jun 1936, *Popov s.n.* (AA!). KYRGYZ ALATAU. Kyrgyz Alatau range, Taldybulak gorge, 7 Jun 1936, *Mihajlova and Popova* s.n. (TASH!); Kumbel pass, 5 Jul 1984, *Nelina and Chubarova s.n.* (AA!); western extremity of the Kyrgyz Alatau, 23 May 1984, *Nelina and Chubarova s.n.* (AA!); Kaindy river valley, 21 May 1984*, Chubarova s.n.* (AA!); Sogety river valley, 23 May 1984, *Nelina and Chubarova s.n.* (AA!).

***Tulipa dasystemonoides* Vved.**

**Specimens examined:—**KYRGYZ ALATAU. Aspara river valley, 4 May 1976, *Akhmetova s.n*. (AA!). WESTERN TIAN SHAN. Aksu-Zhabagly Nature Reserve, along the Zhabagly river, 20 Jul 1968, *Orazova s.n.* (AA!); Aksu-Zhabagly Nature Reserve, 1955, *Tursunmetova and Frolova s.n.* (TASH!); same loc., 14 May 1935, *Dmitrieva s.n.* (AA!); Zhabagly river basin, Kishi-Koyandy tract, 9 Jul 1948, *Vaganova s.n.* (TASH!); same loc., 21 Jun 1948, *Gubajdullin s.n.* (TASH!); Zhabagly river valley, watershed between Chushka-Bulak [Shoshkabulak] and Aynakol, 26 Jun 1955, *Tursunmetova and Frolova s.n.* (TASH!); Zhabaglysu river, 18 Aug 1921, *Abolin and Popov s.n.* (TASH!); Kishi Kaindy pass, 2 Jul 1948, *Korovin s.n.* (TASH!); same loc., 5 Jun 2001, *Mal’tzev s.n.* (TASH!); same loc., 6 Jul 1948, *Kultiassov s.n.* (MW 0815459!); (MW 0815460!); same loc., 19 Jul 1951, *Golubev s.n.* (MW 0815461!); Zhabaglytau, near the glaciers of the Zhabagly-su river, 31 Jul 1928, *Granitov s.n.* (TASH!); Maydantal river, 5 Sep 1921, *Abolin and Popov s.n.* (TASH!); 12-15 km from Dorofeyevka [Pervomayevka] village, to the Uluchur pass, 11 Jun 1923, *Simonova s.n.* (TASH!); Kergely gorge, 7 May 2019, *Sitpaeva s.n.* (NUR!); Karzhantau ridge, Mynbulak, 9 Jun 1940, *Makarchuk s.n.* (AA!); Ugam ridge, Satyl-say gorge, 16 Jul 1954, *Pavlov s.n.* (AA!); Talas Alatau, upper reaches of the Topchak-su river, 29 Jul 1931, *Pavlov s.n.* (MW 0815458!); Talas Alatau, lake Aynakul [Aynakol], 10 Jul 1958, *Golubev s.n.* (MW 0815462!); Zhabagly tau, slope of the Toylek river, 2 Aug 1928, *Granitov s.n.* (TASH!); Aksay river gorge, 7-8 Jul 1922, *Popov s.n.* (TASH!); Ulken Kaindy pass, 13 km from Zhabagly and 85 km southwest of Dzhambul [Taraz], 4 Aug 1996, *Harper and Dzheffri s.n*. (E 19961683!); Ulken Kaindy pass, 6 Jul 1933, *Linczevsky s.n.* (AA!); upper part of the watershed between Yrgaily and Taldybulak, 22 Jul 1960, *Karmysheva s.n.* (AA!); Ugam ridge, Khazar-Teke gorge, 15 Jul 1954, *Pavlov s.n.* (AA!); Talas Alatau, 27 May 1933, *Karmysheva s.n.* (AA!).

***Tulipa dianae-verettiae* J.de Groot & Zonn.**

**Specimeaens examined:—**ALTAI. Altai Pass, 2019, *J.J. de Groot s.n.* (L 3986813).

***Tulipa dubia* Vved.**

**Specimens examined—**WESTERN TIAN SHAN. Aksu-Zhabagly Nature Reserve, near Kyzkol, 7 Jul 1939, *Fedorov s.n.* (LE!); near Kokuyrum lake, H-2500m, 1989, *Ivashchenko and Olontseva s.n.* (personal observation); upper reaches of the Ulken-Aksu river, H-2700m, 1989, *Ivashchenko and Olontseva s.n.* (personal observation); headwaters of the Kokseki river, H-2820m, 1989, *Ivashchenko s.n.*(personal observation).

***Tulipa greigii* Regel**

**Specimens examined:—**MOIYNKUM. Moiynkum, Maykuduk, 30 May 1931, *Astapova s.n.* (MW 0815549!); Chu, 23 Apr 1939, *Pavlov s.n.* (MW 0815548!); Zhambyl mountains, Chagyrly [Shagyrly] ridge, 1 Jun 1926 *Drobov s.n.* (TASH!). TURKESTAN. Keles river basin, Chernyayevka [Zhibek Zholy] village, hillsides along Khan aryk, 16 Apr 1921, *Abolin s.n.* (TASH!). WESTERN TIAN SHAN. Alymtau mountain, 3 Jun 1960 *Pryakhin et al. s.n.* (TASH!); mount Alymtau, 8 Apr 2024, Kubentayev et al. s.n. (NUR!); near Chanak station (Chanak-Arys), 6 May 1941, *Butkov s.n.* (TASH!); near Shengeldy station, Arys-Tashkent road, 27 Apr 1957, *Soskov s.n.* (TASH!); 15 km south of the Alymtau mountains, 21 Jun 1947, *Botschantzev s.n.* (TASH!); near Zhilga station, 19 Apr 1925, *Linczevsky s.n.* (TASH!); Kazygurt mountains, 7 Jul 1922, *Baranov s.n.* (TASH!); Kaplanbek tract, 11 Apr 1921, *Vvedensky s.n.* (TASH!); Darbaza, May 1922, *Korovin s.n.* (TASH!); Shymkent, 12 Apr 1932, *Zhugina s.n.* (TASH!); Alatau mountains, stony slope along the Zhabagly river, 8 Jun 1924, *Mokeeva s.n.* (TASH!); Stolovaya mountain, 22 Apr 1960, *Pratov s.n.* (TASH!); Boraldaytau, Darbaza 1908, *Minkwitz s.n.* (LE!); near Saryagash station, 15 Apr 1924, *Vvedensky s.n.* (TASH!); Aksu river, Maybulak gorge, 9 Aug 1922, *Baranov s.n.* (TASH!); Karatobe, under Maybulak settlement, 5 Aug 1922, *Baranov s.n.* (TASH!); Kuyuk pass, 6 Apr 1916, *Rajkova s.n.* (TASH!); Tulkubas station, 26 Apr 1933, *Botschantzeva s.n.* (TASH!); Burnoye station, 3 May 1933, *Botschantzeva s.n.* (TASH!); Karzhantau mountains, 28 May 1922, *Drobov s.n.* (TASH!); Karabastau tract, 22 Jul 1908, *Knorring s.n.* (TASH!); the foothills, near Abail station, 7 Jun 1924, *Gomolitsky s.n.* (TASH!); Shakpak passage, (Burnoye-Abail), 3 May 1933, *Botschantzeva* (TASH!); near Saryagash station, 15 Apr 1924, *Korovin s.n.* (BRNU 472409!); (US 3006783!); (AA!); Aksu canyon plateau, 14 May 2021, *Manabaeva s.n.* (NUR!); Aksu-Zhabagly Nature Reserve, 1998, *Ivashchenko s.n.* (E 20000894!); Kishi Kaindy valley, 10 km south-east of Zhabagly and 85 km south-west of Zhambul, 8 Aug 1996, *Harper and Dzheffri s.n.* (E 19961692!); Kuyuk mountains, upper reaches of the Saya-su river, 4 Jun 1936, *Chilikina s.n.* (MW 0815530!); Saya-su tract, 5 Jun 1935, *Nikolaev s.n.* (MW 0815528!); the upper reaches of the Bol’shoy Baldyberek river, 28 Jul 1922, Baranov s.n. (TASH!). CHU-ILI RANGE. near Shokpar station, 23 Apr 1949, *Botschantzev s.n.* (TASH!); same loc., 27 Apr 1937, *Dmitrieva and Gorbunova s.n.* (AA!); same loc., 8 May 1936, *Dmitrieva s.n.* (AA!); Korday pass, 14 May 1920, *Titov s.n.* (TASH!); same loc., 18 Apr 1880*, Fetissov s.n.* (LE!); same loc., 25 May 2012, *Vladykina and Alibekov* (KG!); Aksuek tract, Passage No. 73, 29 Mar 1975, *Lyashenko s.n.* (KG!); (AA!); Chu-Ili mountains, 12 May 1951, *Pavlov s.n.* (MW 0815550!); Aksuek road, junction No. 73, 112 km from Aksuek, 26 Apr 1975, *Lyashenko s.n.* (AA!); Khantau mountains, 25 May 1937, *Dmitrieva s.n.* (AA!); in the steppe between 2-3 peaks on the Ospe-Moyynty tract, 12 May 1951, *Pavlov s.n.* (AA!); Anrakhay station, 18 May 1934, *Popov s.n.* (AA!); at the Ala-Aigyr turnout, 10 May 1936, *Dmitrieva s.n.* (AA!); near Otar, Apr 1968, *Lapshina s.n.* (KNU!, TK!). TRANS-ILI KUNGEY ALATAU. Karasuly mountains, Argayty [Yrghayty] river, 12 Jun 1926, *Abolin s.n.* (TASH!); Kalguty river, 24 Jun 1926, *Abolin s.n.* (TASH!). KYRGYZ ALATAU. Aulie-ata city, Tekturmas, 27 Apr 1934, *Ponomareva s.n*. (TASH!); Kyrgyz Alatau range, Kyzyl-Kaynar sai, 23 Jul 1931, *Kudryashev s.n.* (TASH!); near Podgornoe [Kumaryk], 31 Mar 1916, *Sovetkina s.n.* (TASH!); near the Granitogorsk village, 2 May 1985, *Loginova s.n.* (TK!). KARATAU. Karatau mountains, Karasay plateau, 14 May 1934, *Pjataeva s.n.* (TASH!); same loc., 12 May 1936, *Pjataeva s.n.* (TASH!); Karatau mountains, 10 May 1936, *Mironov and Pazij s.n.* (TASH!); same loc., 28 Jun 1923, *Mokeeva s.n.* (TASH!); same loc., 24 Apr 2013, *Gabdullin s.n.* (KG!); same loc., 5 Apr 1930, *Lipschitz s.n.* (MW 0815516!); same loc., 12 May 1934, *Tekut*’*ev s.n.* (MW 0815522!); Zhylagan-ata cave, 17 May 1934, *Pjataeva s.n.* (TASH!); same loc., May 1936, *Pjataeva s.n.* (TASH!); Asy river basin, Berkara tract, 20 Jun 1925, *Sovetkina s.n.* (TASH!); Berkara valley, 70 km west of Jambul, 1 Aug 1996, *Harper and Dzheffri s.n.* (E 19961674!); Berkara gorge, 15 Apr 2024, *Satekov s.n.* (personal observation); Kulan mountains, 5 May 1986, *Kuprijanov s.n.* (KG!); mount Kulantau, slope to the Kulan river, 28 May 1922, *Drobov s.n.* (TASH!); Yrgayly say, 15 May 1934, *Pjataeva s.n.* (TASH!); Mynzhylky massif, Almaly-say gorge, 17 May 1977, *Kamelin et al. s.n.* (LE!); in the foothills of the Karatau mountains, near the village of Babay-Kurgan, 22 Apr 1930, *Lipschitz s.n.* (BRNU 472408!); (S15-24388!); (US 3006785!); Bayaldyr gorge, 11 Jul 1972*, Samojlova s.n.* (NUR!); near Terekty, Terekty cordon, Borolday mountain, 17 Apr 2022, *Kubentaev and Alibekov s.n*. (NUR!); near Zhanatas, near the Aktogay river, 1 May 1984*, Russanovich and Schvetsov* *s.n.* (MA 700748!); Shayanbi pass, 5 Apr 1930, *Lipschitz s.n.* (MW 0815517!); dzhon Degeres tract, 4 Jun 1935, *Hlebnikova s.n.* (MW 0815519!); (MW 0815520!); (MW 0815521!); slope of the Kyzylbulak river, 10 May 1934, *Tekut'ev s.n.* (MW 0815523!); (MW 0815529!); Arkharly-tau mountain, 12 May 1934, *Chilikina s.n.* (MW 0815524!); Zhylandy say gorge, 21 May 1934*, Tekut’ev s.n.* (MW 0815525!); gorge on the road from the village of Kurish to the Kyzylbulak river, 10 May 1934, *Tekut'ev s.n.* (MW 0815526!); near Khantagi cordon No. 2, 6 Apr 2024, *Kubentayev et al*. *s.n*. (NUR!); Boraldaytau, 4 km northwest of Boralday spring, Jul 1960, *Khassanov s.n.* (TASH!); .

***Tulipa heteropetala* Ledeb**

**Specimens examined:—**ZAYSAN. Near the village of Boran, 21 Apr 2023, *Kotuhov s.n.* (ABG 00060!); ALTAI. Narymsky ridge, near Kaynar village, *Kubentaev s.n.* (NUR!); between villages Altay and Maraldy, 20 May 2023, *Satekov s.n.* (personal observation). TARBAGATAI. Near Akzhar village, 26 Apr 2023, *Kotuhov s.n.* (ABG 00040!).

***Tulipa heterophylla* (Regel) Baker**

**Specimens examined:—**TRANS-ILI KUNGEY ALATAU. Lake Issyk, 2 Jun 1931, *Botschantzev s.n.* (TASH!); Malaya Almatinka river, 1 Jun 1917, *Abolin s.n.* (TASH!); Shelek river, Amanzhol tract, 14 Jul 1916, *Abolin s.n.* (TASH!); river Talgar, 4 Jul 1916, *Abolin s.n.* (TASH!); Turgen river, Kokpak-tas tract, 10 Jul 1916, *Abolin s.n.* (TASH!); Kokashyk pass from Malaya Almatinka to Talgar, 10-23 Jun 1918, *Titov s.n.* (TASH!); Aksay gorge, Sataldy river valley, 23 Jul 1927, *Dingelshtedt and Sovetkina s.n*. (TASH!); Bolshoe Almaty gorge, 25 Jun 2018, *Veselova et al. s.n.* (NUR!); near Almaty, Kaskelen river valley, 9 Jul 2014, *Danihelka s.n.* (BRNU 653845!); Almaty reserve, upper reaches of the Kazachka river, 31 May 1933, *Popov s.n.* (BRNU 472411!); (US 3006786!); (MW0815552!); same loc., 19 Jul 1940, *Bocharova and Serebrjakov s.n.* (MW0815559!); right bank of the Kaskelen river, 9 Jun 2014, *Danihelka s.n.* (BRNU 653845!); near the bank of the Malaya Almatynka river, H-2500 m, 17 Jun 1936, *Nikolaev s.n.* (MW0815554!); near the bank of the Malaya Almatynka river, H-3400m, 28 Aug 1936, *Pavlov s.n.* (MW0815555!). KETMEN-TERSKEY ALATAU. Near Saryzhas village, 21 May 1932,*Lipschitz s.n.* (MW0815553!).

***Tulipa iliensis* Regel**

**Specimens examined:—**KETMEN-TERSKEY ALATAU. near the village of Podgornoye, 11 May 1932, *Lipschitz s.n.* (MW0815580!); (MW0815581!); Sumbe river valley, 21 May 1990, *Seisums s.n.* (K001767094!); near Kegen settlement, Chuladyr ridge, 8 May 1954, *Goloskokov s.n.* (TK!); Ketmen Ridge, upper reaches of the Sumbe river, 19 Jul 1963, (TK!).

***Tulipa ivasczenkoae* Epikt. & Belyalov**

**Specimens examined:—**DZUNGARIAN ALATAU. Mts. Chulak; Aiyrkezen, upper third of stony northern slope (1100 m. a. s. l.), 26 Apr 2013, Epiktetov and Belyalov» (ALTB, iso – LE).

***Tulipa jansii* J.J. de Groot & Zonn.**

**Specimens examined:—**BALKHASH-ALAKOL. Ily valley north of Kapchagay. *J.J. de Groot and B.J.M. Zonneveld* (holotype L4513065).

***Tulipa kaufmanniana* Regel**

**Specimens examined:—** KARATAU. Asy river basin, Berkara tract, 20 Jun 1925*, Sovetkina s.n.* (TASH!). WESTERN TIAN SHAN. Aksu-Zhabagly reserve, Aynakol tract, 30 Jul 1956, *Abdullaeva s.n.* (TASH!); same loc., 26 Jun 1955, *Tursunmetova and Frolova s.n.* (TASH!); Aksu-Zhabagly reserve, Taldy-Bulak river valley, 25 Jun 1948*, Korovin s.n.* (TASH!); same loc., 28 May 1927, *Mokeeva and Linczevsky s.n.* (TASH!); Kishi Kaindy river gorge, 21 Jul 1928, *Granitov s.n.* (TASH!); Zhabagly tau, Ulken Koyandy [Ulken Kaindy] pass, 27 Jul 1928, *Granitov s.n.* (TASH!); Zhabagly river basin, Kishi-Koyandy [Kishi Kaindy] tract, 21 Jun 1948, *Vaganova s.n.* (TASH!); same loc., 27 Jun 1952*, Cvelev s.n.* (LE!); Alatau mountains, Zhabagly-Su gorge, 9 Jun 1924, *Mokeeva s.n*. (TASH!); 12-15 km south of Dorofeyevka [Pervomayevka] village, to the Uluchur pass, 11 Jun 1923, *Simonova s.n.* (TASH!); Zhabaglytau, 1/2 km south of lake Kyzolgen kol, 28 Jul 1928, *Granitov s.n.* (TASH!); Ulken Kaindy pass, 6 Jul 1933,  *Linchevskij s.n.* (LE!); Koyandy tract, 3 Aug 1969,  *Khamitova s.n.* (KG!); Karzhantau mountains, 20 Apr 1933, *Vvedensky s.n.* (BRNU 472414!); (US 3006789!); (US 3006784!); Kergely gorge, 7 May 2019, *Sitpaeva s.n.* (NUR!); Aksu canyon, 14 May 2021, *Manabaeva s.n*. (NUR!); Kaska-Bulak valley, 20 km south-east of Zhabagly and 85 km south-west of Dzhambul [Taraz], 7 Aug 1996, *Harper and Dzheffri s.n*. (E 19961689!); near Mashat, 9 Apr 2024, *Kubentayev et al*. *s.n*. (NUR!); near Iirsu, 10 Apr 2024, *Kubentayev et al*. *s.n*. (NUR!); Sary-Aygyr gorge, Sairam-Ugam ridge, 13 May 1989, *Ivashchenko s.n.* (KNU!); bank of the Baldyberek river, 2 Aug 1922, *Baranov s.n.* (TASH!); on the Maydantal river, 4 Jun 1909, *Polovoj s.n.* (TASH!).

***Tulipa kolbintsevii* Zonn.**

**Specimens examined:—**DZUNGARIAN ALATAU. Taskora Valley, H-600 m, Mar 2010, *J.J. de Groot s.n.* (L 0821329!).

***Tulipa kolpakowskiana* Regel**

**Specimens examined:—**MOIYNKUM. Near Tasotkel, between Lenin Zholy and Tasotkel, 12 Apr 2024, *Kubentayev et al*. *s.n*. (NUR!); near Karakemer, 11 Apr 2024, *Kubentayev et al*. *s.n*. (NUR!); Left bank of the Chu river, 7 km south-west of Novo-Troitskoye [Tole Bi], 16 May 1916, *Sovetkina s.n.* (TASH!).. TRANS-ILI KUNGEY ALATAU. Between Chemolgan [Shamalghan] and Kargalinka, 26 Apr 1920, Titov s.n. (TASH!); Uzynagash, 26 Apr 1920, Titov s.n. (TASH!); Kastek river gorge, 24 May 1920, Sovetkina s.n. (TASH!); Near Talgar, slopes to Baybulak river, 13 May 1931, *Botschantzev s.n.* (TASH!); near Kaskelen, 7 May 1931, *Botschantzev s.n.* (TASH!); near Almaty, 9 May 1931, *Botschantzev s.n.* (TASH!); near “Werny” [Almaty], May 1917, *Titov s.n.* (TASH!); same loc 18 Apr 1917, *Titov s.n.* (TASH!); near Talgar, foothills of the right bank of the Talgar river, 12 May 1931, *Botschantzev s.n.* (TASH!); near Talgar village, in the foothills of the Zailiyskiy Alatau, 26 Apr 1930, *Granitova s.n.* (BRNU 472415!); (US 3006790!); Issyk river, 4 Apr 1916, *Abolin s.n.* (TASH!); Malaya Almatinka river, 15 May 1917, *Abolin s.n*. (TASH!); Bol’shaya Almatinka river, 7 Apr 1916, *Abolin s.n.* (TASH!); Zailiyskiy Alatau, 29 Apr 1932, *Lipschitz s.n.* (TASH!); foothills of the Zailiyskiy Alatau, near “Werny” [Almaty], 6 May 1920, *Sovetkina s.n.* (TASH!); Kaskelen river, 18 Apr 1916, *Abolin s.n.* (TASH!); Issyk lake, 2 Jun 1931, *Botschantzev s.n.* (TASH!); near Uzynagash, 2 May 1951, *Pavlov s.n.* (MW 0815643!); steppe behind Shamalghan station, 21 Apr 1939, *Pavlov s.n.* (MW 0815642!); (KNU!); in the foothills of the Zailiyskiy Alatau, Talgar, 26 Apr 1930, *Granitova s.n.* (NY 02681281!). DZUNGARIAN ALATAU. Near Araltobe, Koyandysay, Togay, 25 Jun 1972, *Tajzhanov s.n.* (TASH!). CHU-ILI RANGE. Near Shokpar station, 23 Apr 1949, *Botschantzev s.n.* (TASH!); Korday pass, 14 May 1920, *Titov s.n.* (TASH!); Chu, 23 Apr 1939, *Pavlov s.n.* (MW 0815641!). KYRGYZ ALATAU. City of Aulie-ata [Taraz], 27 Apr 1934, *Ponamareva s.n.* (TASH!); near Merke, 16 Apr 2022, *Kubentayev and Alibekov s.n.* (NUR!); near Merke, 11 Apr 2024, *Kubentayev et al*. *s.n*. (NUR!)

***Tulipa korolkowii* Regel**

**Specimens examined:—** TURKESTAN. Alka-kum [Alkakolkum] sands, sands on the road from Bes-kutan to Togay Ryndy tract, 9 May 1947, *Botschantzev and Butkov s.n.* (TASH!); between Akkala and Koksaray reservoir, 7 Apr 2024, *Kubentayev et al*. *s.n*. (NUR!). WESTERN TIAN SHAN. Chanak station, 10 Apr 1920, *Popov s.n.* (TASH!); near Darbaza station, hills near Kyz-kuduk well, 28 Mar 1937, *Gnezdillo s.n.* (TASH!); Darbaza station, 10 Apr 1920, *Rajkova s.n.* (TASH!); 30 km north-west of Tashkent, near the village of Kaplanbek, 13 Apr 1960, *Ubajdullaev s.n.* (TASH!); near Darbaza station, 6 Apr 1931, (TASH!); near Kaplanbeck, 7 Apr 1947, *Sumnevich and Dzhangurazov s.n.* (TASH!); same loc., 12 Apr 1956, *Nesmiyanova s.n.* (TASH!); same loc., 27 Mar 1922, *Vvedensky s.n.* (TASH!); same loc., 9 Apr 1957, *Adylov s.n.* (TASH!); same loc., 19 Mar 1963, *Adylov s.n.* (TASH!); same loc., 11 Apr 1921, *Vvedensky s.n.* (TASH!); same loc., 16 Apr 1921, *Popov s.n.* (TASH!); same loc., 11 Apr 1921, *Vvedensky s.n.* (MW 0815651!); middle part of the Keles river basin, Kaplanbek tract, 9 Apr 1921, *Abolin s.n.* (TASH!); Stolovaya mountain, 22 Apr 1960, *Pratov s.n.* (TASH!); near Kaplanbeck, 28 Mar 1922, *Korovin and Vvedensky s.n.* (BRNU 472428!); (US 3006721!); Mount Alymtau, 8 Apr 2024, *Kubentayev et al*. *s.n*. (NUR!).

***Tulipa kujukense* J.J. de Groot & Zonn.**

**Specimens examined:—**WESTERN TIAN SHAN. Kuyuk Pass. *J.J. de Groot s.n.* (L4513067).

***Tulipa lehmanniana* Merckl**

**Specimens examined:—** BETPAK-DALA. Near Burubaytal, 15 Apr 2022, *Kubentayev and Alibekov s.n.* (NUR!); Kashkanteniz, 15 Apr 2022, *Kubentayev and Alibekov s.n.* (NUR!). BALKHASH-ALAKOL. Near Iliyskaya station, right bank of the Ili river, 8 May 1921, *Titov s.n*. (TASH!); same loc., 15 Apr 1966, *Ageeva s.n.* (KNU!); same loc., 27 Apr 1954, *Burtseva s.n.* (KNU!); right bank of the Ili river, 6-7 km north of the railway bridge over the river, 15 Apr 1966, *Bajgulova s.n.* (KNU!); 2 km south of Iliysk [Konayev], 9 May 1930, *Granitova s.n.* (TASH!); Almaty - Iliyskaya station road, between Kapchagai and Iliysk stations, 4 May 1921, *Titov s.n*. (TASH!); Kurty river valley, between Sarykeliv and Karasu tracts, 10 Jun 1926, *Titov s.n.* (TASH!); south-eastern Pribalkhash’e, 30 km east of Lepsa, Kyspag mountain, 28 Apr 1958, *Rusyaeva s.n.* (LE!); sands 5-6 km north of Iliysk [Konayev] village, 27 Apr 1962, *Raevsky s.n*. (LE!); Kurty experimental station, Kurty, 29 Apr 1930, *Serova et al. s.n.* (LE!); closer along the right bank of the Ili river, 4 km north of the station, 23 Apr 1946*, Tarabaeva s.n.* (KNU!); (MW 0815413!); near Ili the sands, 21 May 1946, *Mamanova s.n.* (KNU!); same loc., 12 Apr 1936, *Bykov s.n.* (KNU!); right bank of the Ili, on the road to Bakanas, 15 Apr 1966, *Lapshina s.n*. (KNU!); Kapshagay tract, on the right bank of the Ili, 18 Apr 1908, *Sokolov s.n*. (TK!); same loc., 7 Aug 1934, *Sokolov s.n.* (LE!); same loc., 7 Apr 1908, *Sokolov s.n.* (LE!); right bank of the Ili, at the Usharal ford, 29 Apr 1913, *Shishkin and Genina s.n.* (TK!)*,* between Maylybas and Zhanabay tracts, 15 May 1913, *Shishkin and Genina s.n.* (TK!), between Iliysk [Konayev] and the Aue-Su tract, 28 Apr 1913, *Shishkin and Genina s.n.,*(LE!); (TK!); Iliyskaya station, 18 Apr 1934, *Geld s.n.* (LE!); (MW 0815412!); (TK!); (US 3006779!); (BRNU 472451!); sands 3-4 km north of Iliyskaya, 22 Apr 1962*, Raevsky s.n.*, (TK); Pribalkhash’e, Malaysary tract, 3 Mar 1909, *Korneev s.n* (LE!). MOIYNKUM. Muyun-Kum tract, 24 Apr 1904, (LE!); near Aulie-ata [Taraz], Burul sands, 5 Apr 1909, *Block s.n.* (LE!); same loc., 20 Apr 1908, *Block s.n.* (LE!); same loc., 7 Aug 1934, (LE!); Moiyun-kum sands, near Karsymbol well, 28 Mar 1914, (LE!); same loc., 11 Apr 1904, *Abramov et al. s.n.* (LE!); Moiyn-Kum, saksaul plots near Passage No. 51, 23 Apr 1948, *Fissjun s.n.* (AA!). sands in lower reaches of the Chu river, 14 May 1930, *Rubtzov s.n.* (AA!). TURKESTAN. Kyzdy-togay tract, 3 km from the Syrdarya river, Alka-kul-kum [Alkakolkum], 16 Apr 1962, *Pjataeva s.n.* (TASH!); Alka-kum [Alkakolkum], Togay-Ryndy tract, 9 May 1947, (TASH!); opposite Arys station, 25 May 1923, *Mokeeva s.n.* (TASH!); sands east of Ryndy tract, 15 May 1926, *Kultiassov s.n.* (TASH!). CHU-ILI RANGE. Chu-Ili mountains, behind Otar station, 22 Apr 1939, *Pavlov s.n.* (KNU!); in the foothills of the May-Zharalgan mountains, 21 Apr 1941, *Kuznetsov s.n.* (AA!).

***Tulipa lemmersii* Zonn., Peterse & J. de Groot**

**Specimens examined:—**WESTERN TIAN SHAN. Machad Pass, 2007, cult. *A. Peterse* (L 0822655); Keltemashat pass, 27 Mar 1983, *Sharipov s.n.* (TASH!); near Mashat, 9 Apr 2024, *Kubentayev et al*. *s.n*. (NUR!).

***Tulipa orthopoda* Vved.**

**Specimens examined:—**KARATAU. Near the tract of Ush-ozen, 4 Apr 1930, *Lipschitz s.n.* (TASH!); Bazhibil pass, 30 Apr 1930, *Lipschitz s.n.* (TASH!); Zhylagan-ata river, 13 May 1934 , *Pjataeva s.n.* (TASH!); Su-alma say, 25 May 1935, *Pjataeva s.n.* (TASH!); Ush-ozen valley, 28 Apr 1990, *Seisums s.n.* (E 19902872!); near Khantagi cordon No. 2, 6 Apr 2024, *Kubentayev et al*. *s.n*. (NUR!). WESTERN TIAN SHAN. Kuyuk pass, 11 Apr 2024, *Kubentayev et al*. *s.n*. (NUR!).

***Tulipa ostrowskiana* Regel**

**Specimens examined:—**TRANS-ILI KUNGEY ALATAU. Near Almaty, Talgar station, Bai-bulak river, 14 May 1930, *Granitova s.n.* (TASH!); near Talgar station, Shubar-agash mountains, 19 Apr 1921, *Titov s.n*. (TASH!); near “Werny” [Almaty], Apr 1917, *Titov s.n.* (TASH!); Issyk river, 4 Apr 1916, *Abolin s.n.* (TASH!); Kaskelen river, 18 Apr 1916, *Abolin s.n.* (TASH!); the foothills, near “Werny” [Almaty], 6 May 1916, *Abolin s.n.* (TASH!); Malyy Kebik river basin, 18 Apr 1934, *Botschantzeva s.n.* (TASH!); near Almaty, Talgar station, 14 May 1930, *Granitova s.n.* (TASH!); same loc., 29 Apr 1930, *Granitova s.n.* (TASH!); foothills of the Zailiyskiy Alatau, near Verny, 6 May 1920, *Sovetkina s.n.* (TASH!); near Talgar village, 4 May 1930, *Granitova s.n*. (BRNU 472417!); (US 3006714!); south-west of the Aksay farm, 4 May 1966, *Lapshina s.n.* (KNU!); Zhinishke, right bank, 22 May 1936, *Goloskokov s.n.* (KNU!); near Almaty, 4 May 1936,  *Goloskokov s.n.* (KNU!); foothills of the Zailiyskiy Alatau, behind the Stalin collective farm, Apr 1947, *Bajgulova s.n.* (KNU!); Kurday pass, 29 Apr 1920, *Titov s.n.* (TASH!); same loc., 14 May 1920, *Titov s.n.* (TASH!); Zhanbas gorge, 28 May 1975, *Lyashenko s.n.* (KG!).

***Tulipa patens* C.Agardh ex Schult. & Schult. f.**

**Specimens examined:—** TOBOL-ISHIM. Near Zheleznodorozhnyy, 1 May 2009, *Perezhogin s.n.* (KSPI!); Kokterek tract, 1 May 2010, *Perezhogin and Borodulina s.n.* (KSPI!); track is 15 km from the Karamendy turn-off, 23 Jun 2009, *Perezhogin s.n.* (KSPI!); near Borki, 1 May 2009, *Perezhogin s.n.* (KSPI!); near Sholaksay, 25 Apr 2009, *Perezhogin and Borodulina s.n.* (KSPI!); in solonetz steppe near lake Iralykol, Arakaragay forestry, 7 May 1921, *Pavlov s.n.* (MW 0815727!); Naurzum Nature Reserve, 4 May 1935, *Levitsky s.n.* (MW 0815724!); same loc., 29 Apr 1931, *Tarchevsky s.n.* (TK!); Arakaragay forest, 8 May 1921, *Pavlov s.n.* (MW 0815422!). SEMIPALATINSK PINE FOREST. Near Semey, Irtysh floodplain, Vasyukhin island, 25 May 1975, *Kovalenko et al. s.n.* (TK!); near Semey, left bank of the Irtysh, 29 Apr 1912, *Shishkin s.n.* (TK!). KOKCHETAV. Lake Bolshoye Chebachee, 13 Apr 2019, *Kubentayev s.n.* (NUR!). TURGAY. 2 km south-east of Aksuat, 4 May 1945, *Voronov s.n.* (MW 0815728!); (MW 0815725!); eastern shore of lake Aksuat, 31 May 1934, *Gorelov s.n.* (MW 0815726!); near Kokalat, 2 May 2009, *Perezhogin s.n.* (KSPI!); Altyn Dala reserve, Uly-Zhylanshyk massif, 8 Jul 2007, *Ivashchenko s.n.*(personal observation)*,* same loc., 10 Jul 2007, *Ivashchenko s.n.*(personal observation)*,* same loc., 12 Jun 2007, *Ivashchenko s.n.* (personal observation); Altyn Dala reserve, a section of the Tasynkuma, 16 Jul 2007, *Ivashchenko s.n.* (personal observation); Altyn Dala reserve, a section of Sarykopa, near lake Lysoe, 16 Jul 2007, *Ivashchenko s.n.* (personal observation); Altyn Dala reserve*,* Uly-Zhylanshyk site, 4 km north-west of Rakhmet, 8 Jul 2007, *Ivashchenko s.n.* (personal observation); Damdy river, 1 May 2010 *Perezhogin et al. s.n.* (KSPI!). WESTERN UPLAND. Upper reaches of the Ulken Kundyzdy river, 19 May 1959, *Denisova s.n*. (MW 0815722!); cordon at the entrance to the territory of the "Korgalzhyn" Nature Reserve, 5 May 2021, *Urazalina s.n.* (NUR!); near Togusken, 25 Apr 2021, *Kubentayev and Alibekov s.n.* (NUR!); same loc., 23 Apr 2023, *Kubentayev et al. s.n.* (NUR!); near Ortau, mount Ortau, 24 Apr 2021, *Kubentayev and Alibekov s.n.* (NUR!); same loc., 13 Apr 2022, *Kubentayev and Alibekov s.n.* (NUR!); near Atbasar, 28 Apr 2021, *Kubentayev and Alibekov s.n.* (NUR!); same loc., 19 Jun 1937, *Winkler s.n.* (TASH!); near Atbasar, Zhabai river, 20 Apr 2023, *Kubentayev et al. s.n.* (NUR!); near Korgalzhyn reserve, 15 km from Amangeldy village, 29 Apr 2019, *Kubentayev s.n.* (NUR!); Ortau-Kyzyltau road, 13 Apr 2022, *Kubentayev and Alibekov s.n.* (NUR!); near Aksu-Ayuly, 22 Apr 2021, *Kubentayev and Alibekov s.n.* (NUR!); along a temporary watercourse near the Keregetasbulak spring, 22 Apr 2021, *Kubentayev and Alibekov s.n.* (NUR!); Spasskiye sopki, 20 May 2005, *Kvach s.n*. (NUR!); outside the Shopa, 17 May 1999, *Mikhailov s.n.* (KG!); Shubarkol, 10 May 1989, *Kuprijanov s.n.* (KG!); same loc., *Mynbaeva s.n.* (personal observation); near Karaganda, 4 May 1988, *Kuprijanov s.n.* (KG!); Karaganda motorway, 10 km north of Aksu-Ayuly, 30 Apr 2007, *Ivashchenko s.n.*(personal observation); Karazhar, 130 km from Astana, 1 May 2008, *Ivashchenko s.n.*(personal observation); Astana-Korgalzhyn road 95 km, 1 May 2008, *Ivashchenko s.n.* (personal observation); Astana-Korgalzhyn road, turn to the dirt road at 151 km, 1 May 2008, *Ivashchenko s.n.*(personal observation); Kiikbay mountain, Akshatau-Agadyr road, 21 Apr 2023, *Kubentayev et al. s.n.* (NUR!); upper reaches of the Sarysu river, valley of the Zhaksy-Sarysu river, west of Zhanaarka station, 21 Jun 1937, *Pazij s.n.* (TASH!); Sarysu river basin, west of the Togusken tract, 15 Jul 1937, *Pazij s.n.* (TASH!); Sarysu river, between the rivers Zhaksy and Zhaman-Sarysu, between Zhanaarka-Saken stations, 12 Jun 1937, *Pazij s.n.* (TASH!); upper reaches of the Sarysu river, near the Buguly mountains, north-east of Saken station, 15 Jun 1937, *Pazij s.n.* (TASH!); upper Atasu river, 20 Jul 1938, *Koroleva s.n.* (TASH!); upper reaches of the Atasu river, 64 km south-southwest of Ktay sopka, 21 Jul 1938, *Koroleva s.n.* (TASH!). ULUTAU. Near Ungirly, 26 Apr 2021, *Kubentayev and Alibekov s.n.* (NUR!); Ulytau mountain, 15 km from Ulytau village, 26 Apr 2021, *Kubentayev and Alibekov s.n.* (NUR!); near Sarlyk, 3 Apr 2024, *Kubentayev et al*. *s.n*. (NUR!). EASTERN UPLAND. Bektauata mountains, 22 Apr 2021, *Kubentayev and Alibekov s.n.* (NUR!); same loc., 20 Apr 2023, *Kubentayev et al. s.n.* (NUR!); Konyrkulzha, Bektauata mountains, 13 Apr 2022, *Kubentayev and Alibekov s.n.* (NUR!); steppe from Sergiopol’ to Zhuz-Agach, 17 Apr 1902, *Saposhnikov s.n.* (TK!); between Semey and Uluguz, 12 Apr 1902, *Saposhnikov s.n.* (TK!); Terensai lake, near Elizavetinka, 3 Jul 1912, *Semenov s.n.* (TK!). KARKARALY. Karkaraly, 25 Apr 1981, *Kuprijanov s.n.* (KG!); Koyandy and Karasu river valley, 17 Apr 1914, *Kutscherovskaya s.n.* (TK!); Kyzylaray mountain, 15 Jun 1960, *Denisova s.n*. (MW 0815723!). ZAYSAN. Black Irtysh river valley, Ashutas massif, 10 May 2001, *Dyachenko s.n.* (1100007563!). ALTAI. Azutau ridge, Mramornaya mountain, 9 May 2001, *Dyachenko s.n.* (ALTB 1100006396!); spurs of the Kurchum range, Kukumbay, 42 km from Kurchum, 6 May 2001, *Dyachenko s.n.* (1100006404!); Narym ridge, near the Kaznakovskaya crossing on the Bukhtarma reservoir, 5 May 2001, *Dyachenko s.n.* (1100007585!); 5 km southeast of the ferry crossing, 11 Jun 1993, *Iwarsson and Nilsson s.n.* (E 19941809!); between villages Altay and Maraldy, 20 May 2023, *Satekov s.n.* (personal observation).

***Tulipa regelii* Krasn.**

**Specimens examined:—**BALKHASH-ALAKOL. 106 km of the old Karaganda highway, the bank of the Kurty river, 30 Mar 2017, *Veselova and Mukhtubaeva s.n.* (NUR!). CHU-ILI RANGE. Khantau mountains, 2 Jul 1968, *Puchkova s.n.* (TASH!); same loc., 12 Apr 1970, *Fissjun and Orazova s.n.* (BRNU 489553!); (US 3008064!).

***Tulipa salsola* Rukšāns & Zubov**

**Specimens examined:—**DZUNGARIAN ALATAU. West of the village of Koktal, 5 May 2012, *Rukšāns s.n.* (GB).

***Tulipa sogdiana* Bunge**

**Specimens examined:—**MANGYSHLAK. Mangyshlak plateau, Tuyesu sands, 12 Apr 2015, *Ishmuratova s.n.* (MANG08859!); near Zharma, 15 Apr 2018, *Ishmuratova s.n.* (MANG01132!); near Sazdytau , 15 Apr 2018, *Ishmuratova s.n.* (MANG01140!); Karakiya depression, 15 Apr 2012, *Imanbaeva s.n.* (MANG08861!). NORTHERN USTYRT Mangyshlak plateau, eastern part of Tuyesu sand, 3 km from Tanash well, 18 May 1980, *Safronova and Ufimtseva s.n.* (LE!); eastern part of the Mangyshlak plateau, 5 km south-east of Sandy, 22 May 1980, *Safronova and Ufimtseva s.n.* (LE!); Tuyesu sands, 19 Apr 2014, *Imanbaeva s.n.* (MANG08862!). SOUTHERN USTYRT. Karynzharyk sands, 20 Apr 2014, *Imanbaeva s.n.* (MANG08860!); Tuyesu sands, 5 May 2019, *Ishmuratova s.n.* (MANG01100!); same loc., 20 Apr 2014, *Imanbaeva s.n.* (MANG08868!); Kogesem, 30 Apr 2009, *Imanbaeva s.n.* (MANG08857!).

***Tulipa suaveolens* Roth**

**Specimens examined:—**SYRT. Near Uralsk, 23 Apr 1911, *Borodin s.n.* (LE!); same loc, 16 Apr 1911, *Borodin s.n.* (LE!); same loc., 1924, *Larin s.n.* (LE!). TOBOL-ISHIM. Kokterek tract, 1 May 2009, *Perezhogin s.n.* (KSPI!); same loc., 2 May 2010, *Perezhogin and Borodulina s.n.* (KSPI!); near Zheleznodorozhnyy, 2 May 2009, *Perezhogin s.n.* (KSPI!); Naurzum Nature Reserve, 13 May 1971, *Perezhogin s.n.* (KSPI!); same loc., 3 May 2010, *Perezhogin s.n.* (KSPI!); same loc., 7 May 1950, *Kazantseva s.n.*(TK!); Naurzum reserve, 1.5-2 km north of lake Kotantal, 9 May 1935, *Demidova s.n.* (MW 0815751!); along the motorway to Naurzum, 5 km to Karamendy, 1 May 2010, *Perezhogin et al. s.n.* (KSPI!); between the villages of Lavrent'evka and Novonezhinka, 8 Jun 1925, *Musanov s.n.* (LE!); Naurzum parish, Sarymoyin mountains, 28 May 1909, *Savich and Kutscherovskaya s.n.* (LE!); 10 km from the Karamendy turn-off, on the Karamendy-Auliyekol road, 26 Apr 2023, *Kubentayev et al. s.n.* (NUR!). CASPIAN REGION. Near Zhanibek, 22 Apr 1950, *Kamenetskaya s.n.* (MHA 0010226!); same loc., 24 Apr 1950, *Kamenetskaya s.n.* (MHA 0010222!); same loc., 21 Apr 1950, *Kamenetskaya s.n.* (MHA 0010229!); same loc., 23 Apr 1950, *Kamenetskaya s.n.* (MHA 0010227!); same loc., 25 Apr 1950, *Kamenetskaya s.n.* (MHA 0010223!); same loc.,1950, *Kamenetskaya s.n.* (MHA 0010224!); near Karaabinskaja [Karaobinskaja] russian-kyrghyz [russian-kazakh] school, 4 May 1895, *Kulyasov s.n.* (MW 0291344!); Caspian steppe *Vagner s.n.* (LE!); 4 versts east of Dossor, 29 Apr 1924, *Serova s.n.* (LE!); near lake Inder,1870, *Smirnov s.n.* (LE!); Makat fishery, 27 Apr 1937, *Nikitin s.n.* (LE!); same loc., 12 May 1923, *Dubjansky s.n.* (LE!); Inder, close to the shore, 2 Jun 1927, *Iljin and Grigorjev s.n.* (LE!); Zhambeyty district, between Khankol and the Ankata kopir, 8 Jun 1928, *Nikitin s.n.* (LE!); near the Sagyz river, 7 May 1924, *Serova* *s.n.* (LE!). BUKEEV. 1 km east of the Voroshilov [Zhanibek] garden, 13 May 1952, *Khramtsov and Tikhonova s.n.* (LE!). AKTOBE. North-east of the settlement of Oiyl, Akshatau, 5 May 1969, *Matyushenko s.n.* (MW 0815750!); near Temir, 29 Apr 1908, *Borodin et al. s.n.* (LE!); Zhuryn station, between sprinkler No. 2 and the water pump wire, 24 Apr 1908, *Koloskov s.n.* (LE!); Temirskoye experimental field, 29 May 1908, *Fedtschenko and Goloskokov s.n.* (LE!). MUGOJARY. Close to Birshogyr station, 30 Apr 1930, *Dombrovsky s.n.* (US 3006722!); (BRNU 472429!); (MW 0815747!); (LE!); (TK!); Mugodzhar mountains, near Birshogyr station, 22 Apr 1909, *Androssov s.n.* (LE!). EMBA. Konesovhoz Emba, 15 Jun 1934, *Samsel s.n.* (MHA 0010264!); between Emba and Ustyurt, the upper reaches of the Maylysay ravine, 1 May 1926, *Spiridonov* *s.n.* (LE!); northern shore of the Aral Sea, Ustyurt, near Koshkar-ata, 15 Apr 1915, *Bukinich s.n.* (LE!); Koshkar-ata, 12 May 1904, *Dubjansky s.n*. (LE!). TURGAY. Altyn Dala reserve, Uly-Zhylanshyk massif, 8 Jul 2007, *Ivashchenko s.n.*(personal observation); same loc., 12 Jul 2007, *Ivashchenko s.n.*(personal observation); near Torgay, 1 May 2009, *Perezhogin s.n.* (KSPI!); Altyn Dala reserve, a section of Sarykopa, near lake Lysoe, 16 Jul 2007, *Ivashchenko s.n.* (personal observation); a track near the Damdy river, 1 May 2010, *Perezhogin et al. s.n.* (KSPI!); near Ulytau, Ulytau-Arkalyk motorway, Arganaty mountains, 23 Apr 2023, *Kubentayev et al. s.n.* (NUR!). WESTERN UPLAND. Cordon at the entrance to Korgalzhyn reserve, 5 May 2021, *Urazalina s.n*. (NUR!); near Korgalzhyn reserve, 15 km from Amangeldy, 29 Apr 2019, *Kubentayev s.n.* (NUR!); near Abay, on the road to Karazhar, 2 May 2008, *Ivashchenko s.n.* (personal observation); Astana-Korgalzhyn road 95 km, 1 May 2008, *Ivashchenko s.n.* (personal observation); Astana-Korgalzhyn road, 1 May 2008, *Ivashchenko s.n.* (personal observation); near lake Kumkol, 3 May 2008, *Ivashchenko s.n.* (personal observation); Kokshetau mountains, 14 May 1958, *Karamysheva s.n.* (LE!); steppe in the north-east of the Kiyakty lake basin, 1914, *Semenov s.n.* (LE!); Atbasar district, Savenkovsky [Savinkovka] experimental plot, 20 Apr 1917, (LE!); Atbasar uyezd, Zhylandy river, 7 Jun 1908, *Kapel’kin s.n.* (LE!); near Atbasar, 3 Jun 1886, *Shadrin s.n.* (TK!); same loc., 30 May 1908, *Kapel’kin s.n.* (LE!); near Zhezkazgan mine, May 1914, *Semenov s.n* (TK!); near Urozhaynoye, 28 May 1999, *Mikhailov s.n.* (KG!). ULUTAU. Near Ulytau, Ulytau-Arkalyk motorway, Arganaty mountains, 23 Apr 2023, *Kubentayev et al. s.n.* (NUR!). ARAL REGION. Koylybay, the foot of Stolovaya mountain, 25 Apr 1930, *Astapova and Samsel s.n.* (MW 0815748!); Bol’shiye Barsuki sands, 4 May 1934, (MW 0815749!); the sands near Shalkar, 20 Apr 1907, *Androssov s.n.* (LE!); Malyye Barsuki sands, 6 km north-east of Karashokat station, 24 Apr 1930, *Gozhev et al. s.n.* (LE!); Tekeli postal station, 4 May 1909, *Savich and Kutscherovskaya s.n.* (LE!); Irgiz district, Bol’shiye Barsuki, 21 Apr 1904, *Dubjansky s.n.* (LE!); between the Bol’shiye and Malyye Barsuki near lake Shubarkol, 29 Apr 1914, *Desyatova s.n.* (LE!); 12 km south-west of Shalkar station, 29 Apr 1926, *Spiridonov s.n.* (LE!); 1.5 km north-west of lake Zhomart, 29 Apr 1926, *Spiridonov s.n.* (LE!); Mylysai tract, 1 May 1926, *Rusanov s.n.* (LE!); Aschelysay tract, 1 May 1926, *Rusanov s.n.* (LE!); between Emba and Ustyurt, 5 km south-west of lake Zhomart, 30 Apr 1926, *Spiridonov s.n.* (LE!); 2-3 km from Shalkar station, 27 Apr 1914, *Desyatova s.n.* (LE!); between Shalkar village and Stary Shalkar lake, 25 Apr 1914, *Desyatova s.n.* (LE!); Karashokat station, 22 Apr 1911, *Dimo et al. s.n.* (LE!); between Bol’shie and Malyye Barsuki, Kandyzhal tract, 30 Apr 1914, *Desyatova s.n.* (LE!).

***Tulipa tarda* Stapf**

**Specimens examined:—**TRANS-ILI KUNGEY ALATAU. Kastek pass, 17 Apr 19164, *Abolin s.n.* (TASH!); Kastek river gorge, 24 May 1920, *Sovetkina s.n.* (TASH!); Shamalgan gorge, Shamalganka river, 5 Apr 2018, *Veselova et al. s.n.* (NUR!).

***Tulipa tetraphylla* Regel**

**Specimens examined:—**TRANS-ILI KUNGEY ALATAU. Saty gorge, 18 Jun 2018, *Shormanova s.n.* (NUR!).KETMEN-TERSKEY ALATAU. In steppis argillosis prope Sarydschas, 17 May 1932, *Lipschitz s.n.* (TASH!); (MW0815777!); Ketmen ridge, Arlygolsay river valley, 2 Jul 1979, *Ikonnikov s.n.* (LE!); Prov. Heptopotamia (Semyretschje). Distr. Kegen. In steppis argillosis prope Sarydschas, 17 May 1932, *Lipschitz s.n.* (LE!); Ketmen ridge, Komirshi river valley, 17 Jul 1979, *Ladygin s.n.* (LE!); Ketmen ridge, Cholkudysu [Shalkudy] river valley, 19 Jul 1979, *Ladygin* *and* *Ikonnikov s.n.* (LE!).

***Tulipa turgaica* Perezhogin**

**Specimens examined:—** TURGAY. Near Amangeldy, 27 Apr 2021, *Kubentayev and Alibekov s.n.* (NUR!); 20 km north of the Accum Sands, 2015, *Perezhogin and Kulikov s.n.* (KSPI!); near Torgay, 1 May 2009, *Perezhogin s.n*. (KSPI!); near Turgay, the bank of the Kabyrga river, 2015, *Perezhogin and Kulikov s.n.* (KSPI!); near Torgay, near lake Altykol, 24 Apr 2023, *Kubentayev et al. s.n.* (NUR!); near Shubalan, 24 Apr 2023, *Kubentayev et al. s.n.* (NUR!); near Tasty, near Keiki-batyr's mausoleum, 24 Apr 2023, *Kubentayev et al. s.n.* (NUR!); near Tasty, 27 Apr 2021, *Kubentayev and Alibekov s.n.* (NUR!).

***Tulipa turkestanica* (Regel) Regel**

**Specimens examined:—**WESTERN TIAN SHAN. Near Darbaza station, hills near Kyz-kuduk well, 28 Mar 1937, *Gnezdillo s.n.* (TASH!); Kuyuk pass, 30 km south-west of Dzhambul, 1 Aug 1996, *Harper and Dzheffri s.n.* (E 19961673!); Сайрам-Угамский ГНПП, долина реки Наут, 22 May 2003, *Ivashchenko s.n.* (NUR!). KARATAU. Mynzhylky Mountain, 8 May 1939, *Pavlov s.n.* (AA!); near Khantagi cordon No. 2, 6 Apr 2024, *Kubentayev et al*. *s.n*. (NUR!); Ush-ozen tract, 8 Apr 1930, *Lipschitz s.n.* (TASH!).

***Tulipa × tschimganica* Botschantz.**

**Specimens examined:—**WESTERN TIAN SHAN. Хребет Каржантау, урочище Юлдыбай, 8 May 2003, *Ivashchenko s.n.* (NUR!).

***Tulipa uniflora* (L) Besser & Backer**

**Specimens examined:—**ALTAI. Ivanovskiy Belok, near Ridder, 5 May 1901, *Krylov s.n.* (MW 0043643!); near the village of Gornaya Ul’binka, spurs of the Ulba ridge, 2 May 2001, *Ebel and Adamov s.n.* (TK!); Kalbinsky ridge. Zhanasu mountains. Near Sadykbay wintering ground, 7 May 2023, *Kotuhov s.n.* (ABG!); Katon-Karagay SNNP, near Katon-Karagai, 26 Apr 2021, *Bolbotov s.n.* (personal observation).

***Tulipa zenaidae* Vved.**

**Specimens examined:—**KYRGYZ ALATAU. Near Merke, 16 Apr 2022, *Kubentayev and Alibekov s.n.* (NUR!); Merke river Gorge, Ungirly tract, 23 Apr 2017, *Veselova and Kudabaeva s.n.* (NUR!).
